# Deep learning–enhanced single-shot triorganelle STED-FLIM imaging of lipid dynamics in living cells

**DOI:** 10.1126/sciadv.aeh3416

**Published:** 2026-09-30

**Authors:** Lu Gao, Beibei Gao, Wei Ge, Tianze Sun, Wenshuang Liang, Li Jiang, Linyong Zhu, Fu Wang

**Affiliations:** ^1^ Med-X Research Institute and School of Biomedical Engineering, Shanghai Jiao Tong University, Shanghai 200240, China.; ^2^ Optogenetics & Synthetic Biology Interdisciplinary Research Center, Shanghai Frontiers Science Center of Optogenetic Techniques for Cell Metabolism, School of Pharmacy, East China University of Science and Technology, 130 Mei Long Road, Shanghai 200237, China.

## Abstract

Lipid homeostasis is orchestrated by rapid exchange and remodeling across the endoplasmic reticulum (ER), lipid droplets (LDs), and mitochondria. However, live-cell visualization of this triorganelle network remains limited by subdiffraction structures, multiplexed labeling burden, and the ambiguity of intensity-only readouts. Here, we introduce a single-shot stimulated emission depletion-fluorescence lifetime imaging (STED-FLIM) workflow that combines Nile Red analogs with deep learning–based demultiplexing to generate compartment-resolved maps of lipid-organelle organization and dynamics. By combining the STED-resolved nanoscale ultrastructure with lifetime-encoded microenvironmental contrast, our approach separates ER, LDs, and mitochondria from a single acquisition and enables automated tricompartment quantification using a lightweight VGG16-UNet segmentation model. This platform captures coordinated remodeling across the ER-LD-mitochondria axis during lipid stress, including ferroptosis- and apoptosis-associated transitions, while simultaneously reporting nanoscale organization and microenvironmental shifts. Together, this strategy provides a practical route to high-spatiotemporal-resolution, lifetime-encoded multiorganelle lipid imaging in living cells.

## INTRODUCTION

Lipid metabolism is the core of cellular physiology, sustaining energy homeostasis while supporting signaling, transport, and the biophysical organization of membranes (*1*–*4*). Crucially, lipid handling is executed through an interorganelle network rather than isolated pathways, with the endoplasmic reticulum (ER), lipid droplets (LDs), and mitochondria forming a central axis for lipid homeostasis (*3*, *5*, *6*). The ER serves as the dominant platform for lipid synthesis and remodeling, such as the biosynthesis of LDs from the ER bilayer (*7*, *8*); LDs buffer excess fatty acids by storing neutral lipids to mitigate lipotoxicity while supplying substrates on demand (*9*, *10*); and mitochondria convert fatty acids into bioenergetic outputs (for example, via β-oxidation) while coupling the membrane state to metabolic function (*11*–*13*). Together, these couplings define the ER-LD-mitochondria axis as an integrated lipid-handling system that supports dynamic lipid homeostasis.

Resolving lipid redistribution and remodeling demands simultaneous live-cell imaging of the ER, LDs, and mitochondria so that lipid flux can be interpreted as coordinated network behavior rather than organelle-specific snapshots (*1*, *14*). Although genetic and chemical labeling strategies have advanced the visualization of organelle morphology and lipid-associated remodeling, many workflows still fall short in practice: Multiplexing is often limited (*15*–*17*), coordinated dynamics are difficult to follow in real time, and resolving the fine structures that shape lipid exchange remains challenging (*6*, *18*, *19*). These constraints are most evident during acute perturbations that rapidly reprogram the membrane state and lipid flux (for example, apoptosis-associated remodeling) when organelle responses unfold on second-to-minute timescales (*20*–*24*). Meanwhile, many relevant structures are subdiffraction, including thin ER tubules, small LDs, and mitochondrial crista remodeling; moreover, the most informative transitions are inherently dynamic (*25*–*27*). Therefore, capturing these processes requires high spatial resolution together with robust time-lapse readouts.

Superresolution microscopy can bridge the spatial gap between molecular-scale processes and diffraction-limited observation. Among prevalent live-cell compatible techniques, structured illumination microscopy typically provides an ∼100-nm resolution at high imaging speeds, whereas stimulated emission depletion (STED) microscopy delivers a substantially higher lateral resolution (often below 50 nm) without computational reconstruction (*28*–*31*). However, both approaches are commonly implemented as intensity-based measurements, and quantitative interpretation can be confounded by staining heterogeneity, local probe concentration, and photobleaching. Fluorescence lifetime imaging microscopy (FLIM) provides an orthogonal contrast that is comparatively less sensitive to probe concentration and excitation fluctuations while remaining responsive to microenvironmental properties such as polarity, viscosity, and packing (*32*–*34*). These parameters differ across cellular membranes and lipid assemblies, enabling environmentally sensitive probes to report dynamically on compartment state in living cells, such as mitochondrial membrane fluidity, lipid order, and organelle identity (*35*). Lifetime-based superresolution imaging has been used to probe the relationship between mitochondrial membrane potential and ultrastructure and to visualize polarity- and order-sensitive lipid environments within mitochondria (*36*, *37*). In principle, STED-FLIM can therefore couple nanoscale morphology to microenvironment-sensitive information, offering a powerful approach to distinguish cellular compartments beyond intensity alone. Yet multiorganelle STED-FLIM is often constrained by labeling burden, channel cross-talk, and reduced temporal resolution when multiple probes and detection paths are required to visualize different organelles in living cells, limiting throughput and compromising dynamic analysis.

To overcome these limitations, we focus on single-stain multiorganelle imaging coupled to computational demultiplexing, in which one broadly distributing probe labels multiple membrane-associated organelles and computational models assign organelle identity. Deep learning has shown that multiple organelle identities can be recognized from limited optical inputs, supporting algorithmic demultiplexing as a practical route for live-cell imaging (*38*–*41*). Although deep learning has also been applied to FLIM, most efforts have emphasized accelerating lifetime analysis rather than compartment assignment (*42*). Organelle segmentation is expected to be more robust when computational models are provided with contrast dimensions beyond intensity, particularly an orthogonal contrast derived from microenvironmentally responsive fluorescence lifetime. A growing class of amphiphilic, environment-sensitive lipid probes is well suited to this strategy because these dyes access diverse membrane-associated structures while reporting local biophysics, and several are compatible with superresolution implementations (*43*–*45*). Among them, Nile Red (NR) provides an attractive entry point: It stains lipid-rich structures with minimal labeling burden and is compatible with STED (*46*–*48*). Moreover, further exploration shows that NR and its analogs exhibit lifetime-encoded microenvironmental information (*45*, *49*, *50*), highlighting their potential in increasing contrast dimensions for image-based deep-learning segmentation. Together, NR-based staining, STED-FLIM imaging, and algorithmic demultiplexing motivate a powerful framework for one-shot, compartment-resolved interrogation of the ER-LD-mitochondria axis in living cells.

Here, we develop a single-shot STED-FLIM workflow using NR analogs and integrate it with a lightweight VGG16-UNet segmentation framework to demultiplex mitochondria, LDs, and ER from single acquisitions. The model operates on full-frame 1024 by 1024–pixel STED-FLIM images to preserve subcellular detail while maintaining computational tractability through frozen pretrained layers, and we find that pseudocolor standardization of lifetime renderings improves segmentation robustness. Applied to time-lapse imaging, this platform enables rapid triorganelle segmentation across sequences and supports quantitative mapping of nanoscale organization and lifetime-encoded microenvironmental changes during lipid stress, including ferroptosis- and apoptosis-inducing conditions. Collectively, our approach provides an experimentally accessible route to simultaneously resolve nanoscale architecture, microenvironmental state, and organelle identity across the ER-LD-mitochondria axis in living cells, enabling mechanistic dissection of organelle interactions and lipid remodeling in living cells.

## RESULTS

### Photochemical profiling identifies NR analogs for triorganelle labeling

NR is a widely used lipophilic fluorophore that becomes strongly emissive in hydrophobic environments, enabling rapid, wash-free labeling of lipid-rich cellular structures. Consistent with broad membrane partitioning, NR can label LDs, mitochondria, and ER, suggesting that tailored NR derivatives might provide a compact probe set for triorganelle lipid imaging. Thus, we selected NR and three structurally related analogs—NR 2OH, NR 3OH, and NR 6CN (Fig. 1A)—to benchmark photochemical properties and cellular staining behavior. We first characterized absorption and emission in organic solvents with different polarities. Compared to NR, NR 6CN showed a pronounced red shift in absorption, which can be attributed to the enhanced electron-withdrawing capability of the cyano (CN) substituent (fig. S1A). Density functional theory calculations for geometry optimization further revealed that the CN group in NR 6CN stabilizes both the LUMO (lowest unoccupied molecular orbital) and the HOMO (highest occupied molecular orbital), producing the narrowest energy gap (0.10166 eV) among the series (fig. S1B). Spectra collected across solvents of increasing polarity showed red-shifted absorption (Fig. 1B and table S1). The solvatochromic shifts between diethyl ether and dimethyl sulfoxide (DMSO) were 57 nm (NR), 55 nm (NR 2OH), 61 nm (NR 3OH), and 44 nm (NR 6CN), with NR 3OH exhibiting the strongest polarity response (fig. S2), consistent with an intramolecular charge-transfer character of the NR fluorophore.

**Fig. 1.**
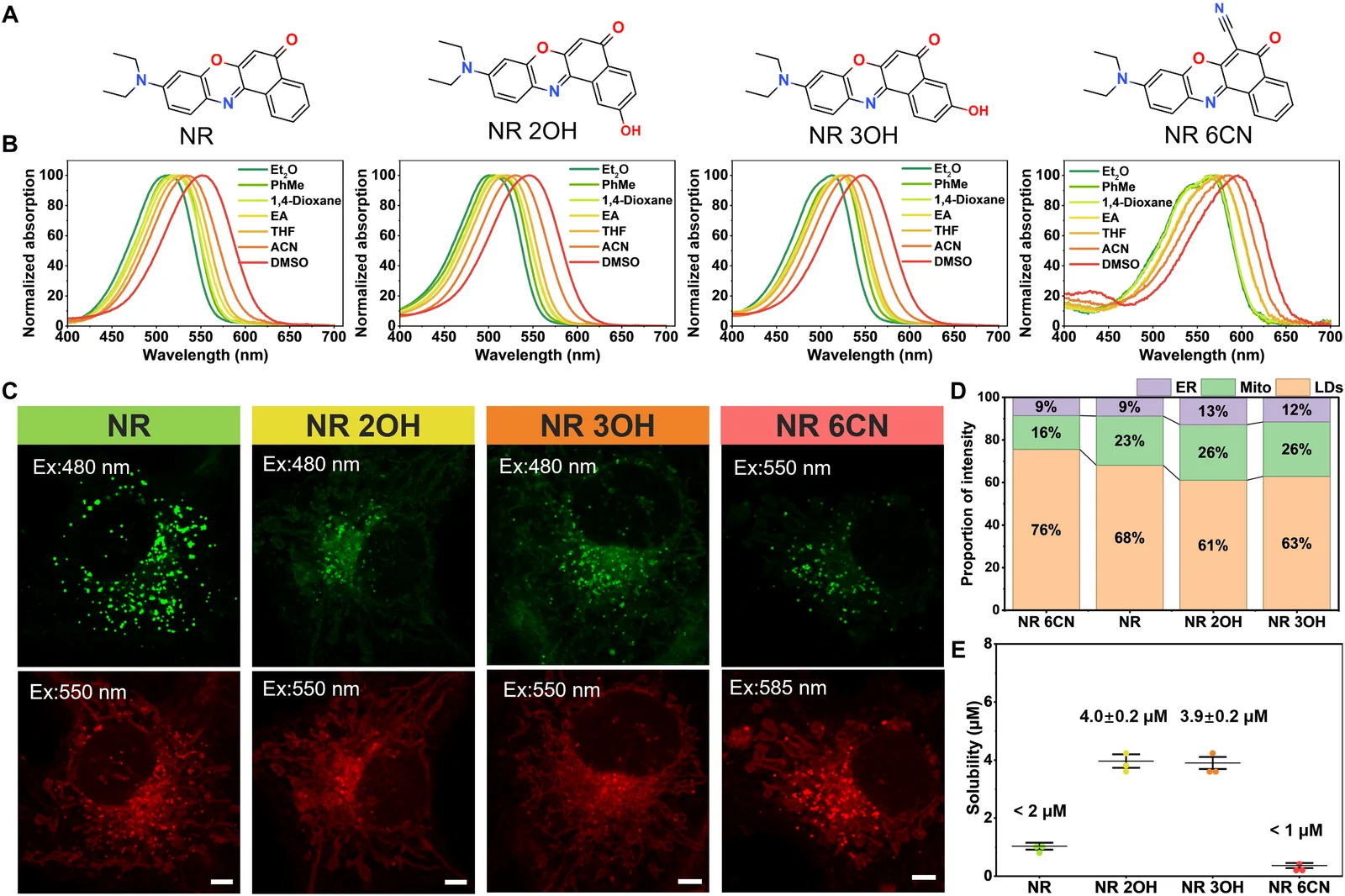
Photochemical characterization and CONF imaging of NR analogs. (**A**) Chemical structures of NR, NR 2OH, NR 3OH, and NR 6CN. (**B**) Absorption spectra of the NR analogs in solvents of different polarity, including 1,4-dioxane, DMSO, ethyl acetate (EA), toluene (PhMe), tetrahydrofuran (THF), ether (Et_2_O), and acetonitrile (ACN). (**C**) CONF images of live COS-7 cells stained with the NR analogs under short- and long-wavelength excitation. Scale bars, 5 μm. (**D**) Relative fluorescence contributions of LDs, mitochondria (Mito), and ER under long-wavelength excitation, with the mean ER intensity set as the reference. (**E**) Water solubility of the NR analogs. Data are the means ± SD (*n* = 3 independent measurements).

Because ER, LDs, and mitochondria present distinct lipid compositions and membrane physical properties, we next compared cellular staining performance. All analogs supported high signal-to-noise ratio imaging at very low concentrations (∼500 nM) and showed good biocompatibility (Fig. 1C and fig. S3). Notably, excitation wavelength altered apparent compartment emphasis: Under shorter-wavelength excitation, staining preferentially highlighted LDs with green emission, whereas longer-wavelength excitation additionally revealed mitochondria and ER with red emission. This wavelength-dependent labeling is consistent with the stronger hydrophobicity of LDs relative to mitochondria and ER, which creates a low-polarity microenvironment that blue shifts probe emission.

We therefore standardized subsequent triorganelle comparisons using long-wavelength excitation (550 nm for NR, NR 2OH, and NR 3OH; 585 nm for NR 6CN). Colocalization with commercial markers confirmed that all four NR analogs labeled LDs, mitochondria, and the ER in living COS-7 cells (figs. S4 to S6). Pearson’s correlation coefficient (PCC) analysis revealed distinct partitioning preferences (fig. S7). For LDs, NR and NR 6CN showed substantially higher correlation with the LD marker (PCC > 0.75) than NR 2OH and NR 3OH (PCC < 0.6). By contrast, NR 2OH and NR 3OH correlated more strongly with mitochondrial markers (PCC ≈ 0.8) than NR and NR 6CN (PCC ≈ 0.7). ER colocalization was broadly similar across the series (PCC ≈ 0.7). On the basis of this comparable performance in ER labeling, we used ER-associated fluorescence as an internal reference to compare relative signal contributions from each compartment (Fig. 1D). The ER signal remained weak across all probes, whereas LD and mitochondrial contributions diverged more strongly. The LD intensity fraction increased from 68% (NR) to 76% (NR 6CN), whereas NR 2OH and NR 3OH reduced LD bias (61 and 63%, respectively). The strong LD preference of NR 6CN is consistent with its lower water solubility and greater enrichment in highly hydrophobic environments (Fig. 1E). Conversely, the higher mitochondrial contribution for NR 2OH and NR 3OH is consistent with more balanced amphiphilicity and larger dipole moments, which may favor association with mitochondrial membranes. Overall, these data establish the NR analog as a tunable, single-stain probe set that supports triorganelle labeling across the ER-LD-mitochondria axis in living cells.

### Depletion-tolerant NR analogs support live-cell time-lapse STED imaging

To achieve visualization of the lipid-associated ultrastructure in living cells, we validated the performance of NR analogs under STED imaging. Relative to confocal (CONF) imaging, all analogs were compatible with STED and enabled visualization of subdiffraction membrane features (Fig. 2A). To estimate effective image resolution, we performed Fourier ring correlation (FRC) analysis on time-resolved STED raw data (*51*). Photon streams were split into odd and even subsets to reconstruct two statistically independent STED images from each acquisition (fig. S8). FRC analysis yielded effective resolutions of ∼140 to 150 nm across the four probes, with NR 3OH performing the best (∼140 nm) and NR 6CN performing slightly less well (∼150 nm) (Fig. 2B and fig. S9). The modestly lower FRC performance of NR 6CN may reflect its less uniform intracellular distribution, which can reduce whole-image resolution estimates (*52*).

**Fig. 2.**
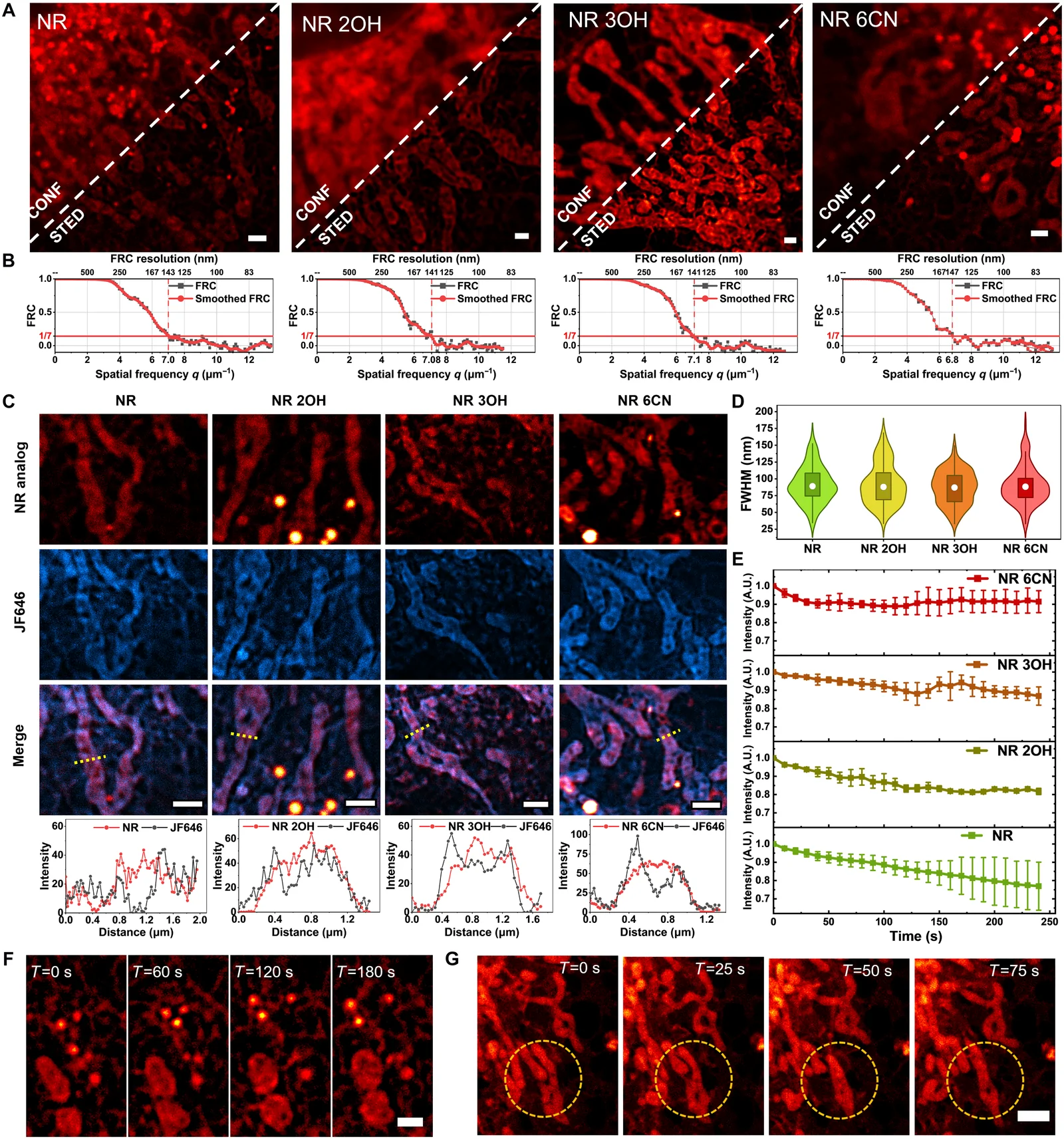
NR analogs enable time-lapse STED imaging in living cells. (**A**) Representative CONF and STED images of COS-7 cells stained with NR (λ_ex_ = 550 nm, λ_STED_ = 775 nm), NR 2OH (λ_ex_ = 550 nm, λ_STED_ = 775 nm), NR 3OH (λ_ex_ = 550 nm, λ_STED_ = 775 nm), and NR 6CN (λ_ex_ = 585 nm, λ_STED_ = 775 nm). Scale bars, 1 μm. (**B**) FRC analysis of whole-image resolution. Resolution was defined as the inverse spatial frequency at which the FRC curve crossed the 1/7 threshold, yielding values of 143, 141, 141, and 147 nm, respectively. (**C**) Colocalization STED imaging of COS-7 cells stained with NR analogs and the outer mitochondrial membrane marker TOM20-Halo labeled with JF646 (λ_ex_ = 650 nm, λ_STED_ = 775 nm). The bottom row shows line profiles along the yellow dashed lines in the merged images. Scale bars, 2 μm. (**D**) Apparent widths of ultrastructural features quantified by Gaussian fitting and reported as FWHM; *n* ≥ 100 measurements per analog. (**E**) Photostability of the NR analogs during continuous STED acquisition, normalized to the initial frame. Data are the means ± SD from three biologically independent replicates. A.U., arbitrary units. (**F**) Time-lapse STED imaging of LD, ER, and mitochondrial dynamics in COS-7 cells stained with NR 3OH. Scale bar, 1 μm. (**G**) Time-lapse STED imaging of mitochondrial fusion in COS-7 cells stained with NR 3OH. The yellow circular selection denotes a mitochondrial fusion event. Scale bar, 2 μm.

To further assess the ability of these lipophilic analogs to delineate complex organelle morphology, we focused on the mitochondrial network, whose dense and dynamic membrane architecture serves as a benchmark for high-performance live-cell imaging. We examined mitochondrial staining in greater detail using dual-color STED imaging with established mitochondrial membrane markers (*53*, *54*). Colocalization with TOM20-Halo labeled by JF646 showed that the NR analogs overlapped with the outer mitochondrial membrane regions while also labeling additional membrane-rich intracellular structures (Fig. 2C). Costaining with an inner mitochondrial membrane marker yielded only partial overlap, and reduced brightness observed in costained samples may reflect altered probe partitioning (fig. S10). Together, these results indicate that NR analogs report mitochondrial membrane–associated regions. To quantify the apparent width of locally resolved membrane features, we further quantified crista-like features generated by the folded inner mitochondrial membrane. Full width at half maximum (FWHM) analysis yielded values of ∼90 nm overall, with the smallest mean width observed for NR 3OH (∼86 nm) (Fig. 2D), consistent with the strong mitochondrial signal performance of NR 3OH.

We next evaluated whether these probes were sufficiently photostable for dynamic superresolution imaging in living cells. During continuous time-lapse STED acquisition, all analogs retained at least 80% of their initial fluorescence after 250 s, with NR 3OH and NR 6CN retaining more than 85% (Fig. 2E), indicating good depletion tolerance and photostability. Given their enhanced mitochondrial contribution relative to NR and NR 6CN, NR 2OH and NR 3OH are well suited for probing mitochondrion-linked dynamics within the ER-LD-mitochondria axis. Although NR 2OH and NR 3OH performed similarly, NR 3OH exhibited nearly twofold higher cellular fluorescence intensity than NR 2OH (fig. S11), enabling lower excitation power and improved compatibility with long-term imaging. We therefore used NR 3OH for time-lapse STED imaging to observe interactions among LDs, mitochondria, and ER (movie S1). Under basal conditions, LD motility coincided with local ER remodeling (Fig. 2F), and mitochondria displayed pronounced dynamics including fusion events (Fig. 2G and movie S2). NR 3OH performed similarly across additional cell lines and instruments (figs. S12 and S13), supporting the generalizability of the imaging workflow. Overall, NR analogs, particularly NR 3OH, combine STED compatibility and photostability sufficient for time-lapse superresolution imaging of triorganelle dynamics in living cells.

### STED-FLIM reports organelle microenvironment states beyond intensity contrast

Staining heterogeneity and convergent organelle morphologies are common in fluorescence imaging, and intensity-based STED images alone can therefore be ambiguous when different compartments produce similar signals, making reliable organelle segmentation challenging. We further validated whether the fluorescence lifetime from STED-FLIM could provide orthogonal contrast to discriminate organelle microenvironments across the ER-LD-mitochondria axis. As expected, NR analogs exhibited polarity-dependent lifetime responses in solvents of different polarity (fig. S14). NR, NR 2OH, and NR 3OH showed increasing lifetimes from low- to high-polarity solvents; NR 3OH exhibited a 0.8-ns change from toluene (3.80 ns) to acetonitrile (4.67 ns). By contrast, NR 6CN showed decreasing lifetime with increasing polarity, with 4.65 ns in toluene and 3.35 ns in DMSO, corresponding to the largest polarity-dependent range (1.3 ns) (table S2).

Motivated by polarity differences across cellular microenvironments, we next assessed STED-FLIM performance in cells (Fig. 3A). Compared with CONF-FLIM, STED-FLIM produced only a slight decrease in whole-cell average lifetime, typically <0.1 ns for most probes (figs. S15 and S16), consistent with the known effect of the depletion laser on fluorescence decay kinetics in time-resolved STED measurements (*55*–*58*). At the organelle level, the lifetime deviation between CONF and STED measurements was generally <0.05 ns and not statistically significant in most cases (fig. S17). STED-FLIM preserved the intrinsic interorganelle lifetime separation of ∼0.3 to 0.4 ns observed by CONF-FLIM (Fig. 3B). In particular, in LDs, NR 6CN showed the longest average lifetime (∼3.7 ns), whereas NR 2OH and NR 3OH were ∼3.4 ns. In mitochondria, lifetimes were more tightly clustered, centered near ∼3.0 ns. In ER, NR 6CN again exhibited the longest mean lifetime (∼2.9 ns), whereas the hydroxylated analogs were slightly shorter (∼2.7 ns). Because this biological separation substantially exceeded the minor instrumental offset introduced by STED, STED-FLIM provided sufficient compartment-resolved lifetime contrast for multiplexed discrimination. We also noted that NR yielded less stable lifetime imaging than the other analogs, with occasionally bright, low-lifetime aggregates (fig. S18), consistent with effects such as high local concentration, aggregation, or microenvironment heterogeneity. Notably, structures with similar brightness in STED intensity images (Fig. 3C) could be separated in STED-FLIM maps (Fig. 3D), indicating that intensity-defined puncta were not exclusively LDs and might include mitochondrion-associated structures. Overall, these phenomena emphasize that lifetime contrast improved interpretability in regions where intensity cues were insufficient.

**Fig. 3.**
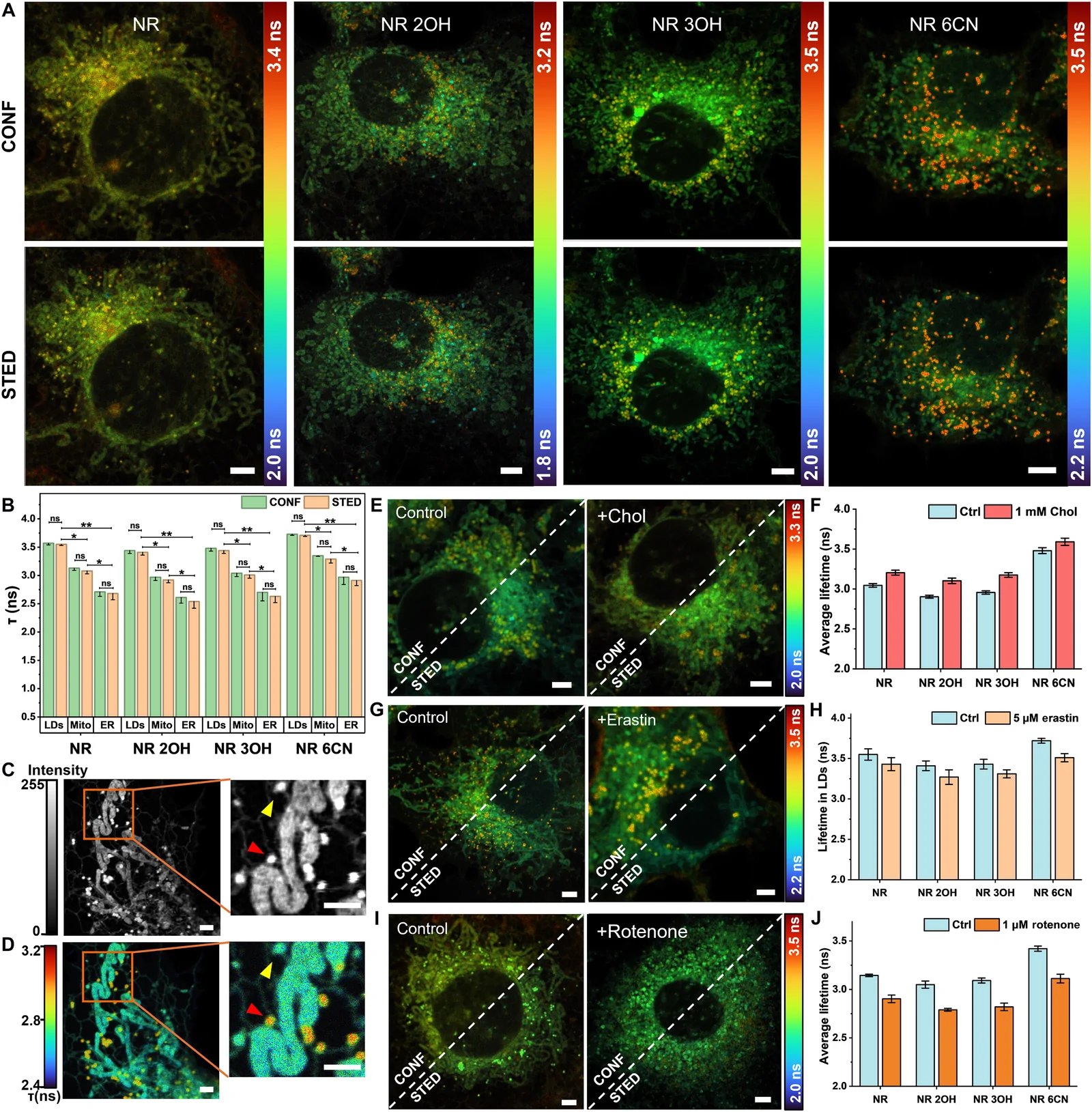
STED-FLIM resolves organelle-associated lifetime contrast and stress-dependent changes. (**A**) Representative CONF-FLIM and STED-FLIM images of COS-7 cells stained with the NR analogs. For each probe, CONF and STED images were acquired from the same field of view. Scale bars, 5 μm. (**B**) Mean fluorescence lifetimes (τ) in LDs, mitochondria (Mito), and ER measured under CONF and STED imaging for the NR analogs. Data are the means ± SD (*n* ≥ 20 cells per analog). Statistical significance was determined by a *t* test (ns, not significant; *P < 0.05; **P < 0.01). (**C**) Representative STED intensity image and (**D**) the corresponding STED-FLIM image of COS-7 cells stained with NR 3OH. Scale bars, 2 μm. (**E**) Representative CONF-FLIM and STED-FLIM images of NR 3OH–stained COS-7 cells without and with cholesterol treatment (1 mM, 30 min). Scale bars, 5 μm. (**F**) Cell-averaged lifetime changes after cholesterol treatment for the NR analogs under STED-FLIM imaging. Data are the means ± SD (*n* ≥ 20 cells per analog). (**G**) Representative CONF-FLIM and STED-FLIM imaging of NR 6CN–stained COS-7 cells without and with erastin treatment (5 μM, 3 hours). Scale bars, 5 μm. (**H**) Lifetime changes in LDs after erastin treatment for the NR analogs under STED-FLIM imaging. Data are means ± SD (*n* ≥ 25 measurements per analog). (**I**) Representative CONF-FLIM and STED-FLIM images of NR-stained COS-7 cells without and with rotenone treatment (1 μM, 20 min). Scale bars, 5 μm. (**J**) Cell-averaged lifetime changes after rotenone treatment for the indicated NR analogs under STED-FLIM imaging. Data are the means ± SD (*n* ≥ 20 cells per analog).

We next validated the lifetime response of NR analogs in organelles to microenvironmental changes. To directly perturb membrane physicochemical properties, we supplemented cells with cholesterol, which is expected to alter lipid packing and polarity in cellular membranes (*48*). Following cholesterol addition (1 mM water-soluble cholesterol, 30 min), STED-FLIM imaging revealed a global increase in lifetimes across compartments (Fig. 3E and fig. S19), with multiple analogs showing notable shifts and an average increase of ∼0.2 ns (Fig. 3F). Repeated imaging of untreated controls yielded fluctuations below 0.05 ns (fig. S20 and movie S3), confirming that the observed lifetime shifts represented a true biophysical response rather than acquisition drift. To determine whether these changes reflected bona fide alterations in membrane state, we independently measured whole-cell microviscosity using the molecular rotor CCVJ and membrane order using Laurdan (*59*, *60*). Cholesterol treatment increased both microviscosity and lipid packing (figs. S21 and S22), consistent with the longer lifetimes of NR analogs measured under the same conditions. Consequently, these results establish that NR analogs can report changes in the lipid environment through fluorescence lifetime under STED-FLIM imaging.

We then assessed lifetime sensitivity under lipid stress conditions, with a particular emphasis on apoptosis. Lipid peroxidation–associated ferroptosis and mitochondrial inhibition–driven apoptosis are both accompanied by remodeling of organelle morphology and membrane state (*61*, *62*). Upon erastin treatment (5 μM, 3 hours), NR analogs reported LD aggregation together with microenvironmental shifts (Fig. 3G and fig. S23). Across NR analogs, lifetimes decreased in both LDs and mitochondria (Fig. 3H and fig. S24), with NR 6CN exhibiting the strongest response in LDs (∼0.2-ns decrease). In contrast, rotenone-induced mitochondrial stress, an apoptosis-inducing perturbation, produced the most pronounced lifetime decreases (∼0.4 ns; Fig. 3, I and J, and fig. S25), in agreement with reduced microviscosity and membrane order during apoptosis-associated remodeling (figs. S21 and S22). Together, these orthogonal measurements suggest that the lifetime changes of NR analogs reflect, at least in part, altered membrane microviscosity and lipid order. Meanwhile, lifetime estimation reflected additional sources of variability beyond within-cell microenvironment differences, including cell line–dependent effects (fig. S26). In addition, the plasma membrane consistently exhibited the longest lifetimes (fig. S27), consistent with a more ordered lipid environment. Although three-dimensional (3D) STED-FLIM imaging is attractive, the current axial resolution did not support reliable organelle identification (fig. S28). Overall, these results establish that STED-FLIM provides a lifetime-encoded microenvironmental dimension that improves triorganelle discrimination and sensitively reports lipid-state remodeling under perturbation.

### A lightweight deep-learning network segments organelles from STED-FLIM images

STED-FLIM provides rich information in distribution, intensity, and lifetime, but rapid identification of ER, LDs, and mitochondria requires computational segmentation. To enable high-throughput analysis of organelle dynamics and interactions, we implemented a VGG16-UNet segmentation model (Fig. 4A). The model combines a VGG16 encoder for hierarchical feature extraction with a UNet-style decoder for pixel-wise reconstruction and operates directly on full-frame 1024 by 1024–pixel STED-FLIM images, thereby preserving subcellular detail without image compression (fig. S29). Training separate binary models for each organelle (LDs, ER, and mitochondria) reduces interclass competition and allows organelle-specific optimization, while on-the-fly data augmentation with Dice-optimized training enables robust segmentation from limited annotated data. Training masks were generated according to predefined criteria on the basis of morphology, intensity distribution, and fluorescence lifetime, with commercial costains used to anchor organelle-specific signatures during annotation (fig. S30).

**Fig. 4.**
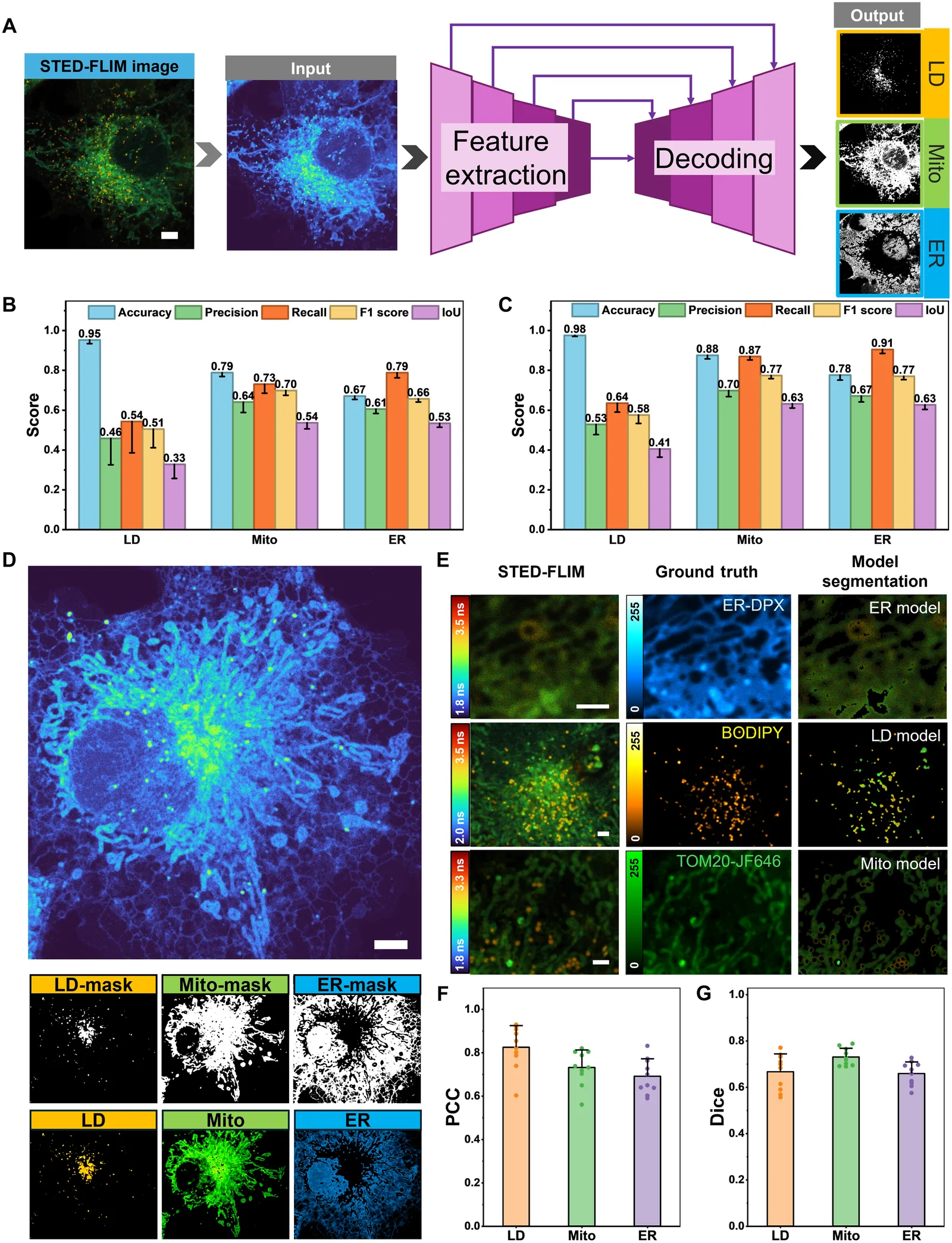
Deep learning–assisted demultiplexing of organelles from single-shot STED-FLIM images. (**A**) Schematic of the VGG16-UNet workflow for organelle demultiplexing from single-shot STED-FLIM images. Grayscale-converted and pseudocolored STED-FLIM images were used as inputs for feature extraction and decoding to generate compartment masks for LDs (LD), mitochondria (Mito), and ER. (**B** and **C**) Segmentation performance of the model between (B) original STED-FLIM inputs and (C) pseudocolored inputs using manually annotated binary masks as ground truth, evaluated by accuracy, precision, recall, F1 score, and IoU. Data are the means ± SD from fivefold cross-validation. (**D**) Representative pseudocolored STED-FLIM image. Corresponding reference masks and model-predicted compartment assignments for LD, mitochondria, and ER. Scale bar, 5 μm. (**E**) Representative comparison between model-predicted compartment assignments and commercial organelle markers (LD, BODIPY 493/503; mitochondria, TOM20-JF646; ER, ER-DPX) acquired in the same cell. Scale bars, 2 μm. (**F**) PCC between model-predicted intensity and commercial organelle-marker intensity for LD, mitochondria, and ER. (**G**) Dice score of different organelles’ prediction accuracy. Data are the means ± SD (*n* ≥ 10 independent samples).

We evaluated model performance using accuracy, precision, recall, F1 score, and intersection over union (IoU), with fivefold cross-validation based on a 4:1 training-validation split. Preprocessing improved performance: Converting STED-FLIM images to grayscale and reassigning pseudocolor produced better results than using the original STED-FLIM images with intensity-weighted lifetime (fig. S31). Compared to original input (Fig. 4B), pseudocolor input improved segmentation performance and stability across folds (Fig. 4C), including an increase of ∼0.1 in accuracy for the mitochondrial and ER models. With pseudocolored input, the mitochondrial and ER models achieved higher overall performance (precision of ∼0.7; F1 score of ∼0.8); the mitochondrial model achieved higher accuracy (0.88), whereas the ER model achieved higher recall (0.91). These results may be related to the variability driven by the microenvironment in the original images (affecting lifetime/contrast distribution) and the sensitivity of the image mask. In addition, using intensity-only maps yielded the weakest performance (fig. S32), consistent with limited contrast between organelles when lifetime information is absent. Therefore, standardizing color and brightness for STED-FLIM images via pseudocoloring improved feature consistency for training.

In addition, the LD model showed a distinct metric profile. Both mitochondrial and ER models converged within 80 epochs (fig. S33), whereas the LD model remained limited, likely due to foreground-background imbalance. This produced high accuracy (0.98) but lower precision, recall, F1 score, and IoU, generally below 0.55. Confusion-matrix analysis confirmed high true-negative rates across all models, reflecting the large background fraction, whereas mitochondria and ER exhibited higher true-positive rates because of their broader distribution (fig. S34). Representative prediction outputs are shown in Fig. 4D. Together, these data establish practical triorganelle segmentation from single-shot STED-FLIM images while indicating that model performance must be interpreted considering organelle-specific distribution patterns rather than any single metric alone.

To assess biological relevance more directly, we compared model-predicted masks with orthogonal organelle markers imaged in the same cells from an independent dataset, including BODIPY 493/503 for LDs, TOM20-JF646 for mitochondria, and ER-DPX for the ER (Fig. 4E). Spatial agreement was quantified using PCC and the Dice coefficient. Across all organelle classes, PCC values exceeded 0.7, and Dice coefficients exceeded 0.6 (Fig. 4, F and G), demonstrating close correspondence between predictions and independently labeled distributions. The model also remained robust across different cell lines and staining concentrations, with typical PCC values above 0.6 (fig. S35), supporting its transferability.

We next compared our approach with conventional phasor-based analysis, a fitting-free and computationally efficient method for lifetime-based segmentation (*63*). Phasor analysis reliably separated organelles with well-separated lifetime populations, notably mitochondria, approaching model performance (fig. S36). However, ER segmentation by phasor analysis was substantially poorer, with lower accuracy and F1 score than the VGG16-UNet model (VGG16-UNet: >0.8 accuracy, >0.8 F1 score; phasor: <0.6 accuracy, <0.3 F1 score), likely because the diffuse and overlapping ER lifetime distribution cannot be cleanly isolated by geometric gating in the phasor space. Because traditional phasor analysis evaluates pixels independently on the basis of decay signatures alone, it struggles when lifetime populations overlap and often requires subjective manual boundary placement (*64*). By integrating lifetime contrast with spatial continuity and morphology, the deep-learning model produced more coherent compartment assignments. Together, these results show that deep learning complements phasor analysis and provides a more robust solution for triorganelle demultiplexing from single-acquisition STED-FLIM images.

### Automated single-shot STED-FLIM time-lapse imaging captures lipid-organelle dynamics under stress

While the preceding experiments establish triorganelle imaging in static frames, interrogating lipid homeostasis requires tracking organelle behaviors and contacts over time. This need is particularly acute during apoptosis, where organelle remodeling can be rapid, asynchronous across organelles, and readily obscured by end-point measurements. Quantitative, scalable capture of these dynamics therefore benefits from time-lapse imaging paired with automated, compartment-aware analysis. However, longitudinal datasets amplify segmentation and tracking challenges, especially when a single lipid probe labels a broad range of membrane-associated structures. We therefore applied our segmentation framework to time-lapse STED-FLIM imaging to enable convenient quantification of dynamic organelle behaviors and interactions.

Using the VGG16-UNet model, mitochondria, LDs, and ER could be segmented across image sequences with high efficiency. We first performed time-lapse STED-FLIM imaging of COS-7 cells stained with NR 3OH during erastin-induced lipid stress (movie S4 and Fig. 5A) and efficiently extracted all three organelles throughout the sequence (fig. S37). Tracking individual droplets with the LD model revealed discrete LD fusion events (Fig. 5B). Integrating the three segmentation masks further highlighted frequent close appositions among LDs, ER, and mitochondria, with all compartments exhibiting persistent motility and remodeling (Fig. 5C), indicating that ferroptosis is accompanied not only by changes in morphology and lifetime contrast but also by highly dynamic interorganelle coupling.

**Fig. 5.**
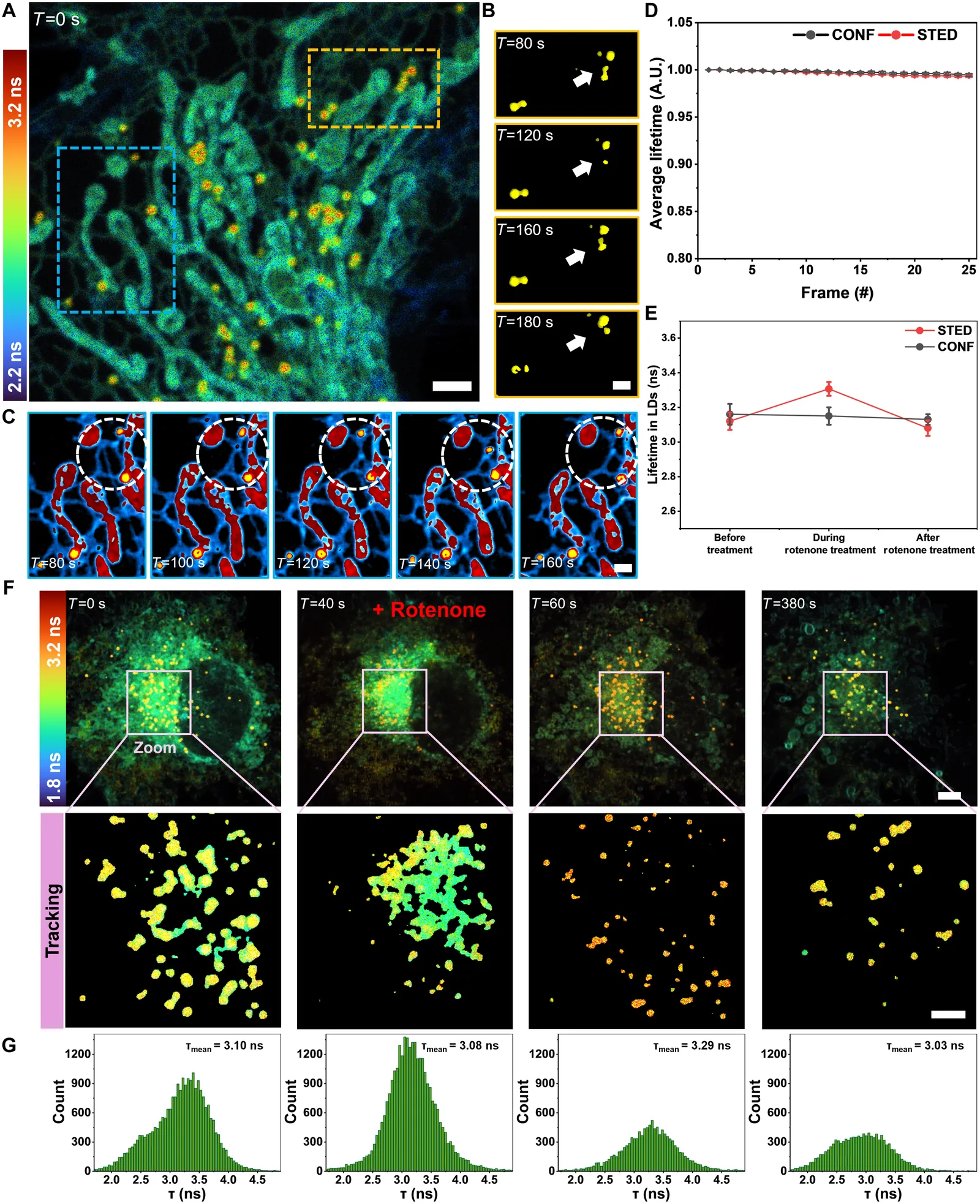
Automated segmentation for time-lapse STED-FLIM images resolves triorganelle dynamics under lipid stress. (**A**) Time-lapse STED-FLIM imaging of COS-7 cells stained with NR 3OH after erastin treatment to induce ferroptosis (frame interval, ∼20 s; acquisition time, ∼10 s per frame). Scale bar, 2 μm. (**B**) Tracking LDs within the boxed region in (A), highlighting an LD fusion event (arrow). Scale bar, 1 μm. (**C**) Overlay of triorganelle segmentation masks for the region highlighted in (A), with LDs in yellow, mitochondria in red, and ER in cyan. The dashed circle marks a representative close-proximity event among the three compartments. Scale bar, 1 μm. (**D**) Cell-averaged fluorescence lifetime trajectories of NR 3OH–stained COS-7 cells during continuous CONF-FLIM or STED-FLIM acquisition (frame interval, ∼20 s; acquisition time, ∼7 s per frame), normalized to the initial frame. Data are the means ± SD of independent experiments (*n* ≥ 3). (**E**) Cell-averaged and LD-specific lifetime trajectories before, during, and after rotenone treatment (∼5 μM), acquired by CONF-FLIM or STED-FLIM. Data are the means ± SD of independent experiments (*n* ≥ 3). (**F**) Time-lapse STED-FLIM imaging of rotenone-treated COS-7 cells during apoptosis-associated remodeling, with LD tracking generated by the LD segmentation model (frame interval, ∼20 s; acquisition time, ∼7 s per frame). Rotenone (∼5 μM) was added at ∼40 s. Scale bars, 5 μm (overview) and 2 μm (zoom). (**G**) Fluorescence lifetime histogram of the zoomed region in (F) and the corresponding mean lifetime.

We next leveraged time-lapse STED-FLIM to probe apoptotic dynamics with higher temporal specificity. Before interpreting dynamic lifetime changes, we quantified measurement stability during repeated acquisition. Under the conditions used here, the depletion laser ($\lambda_{\text{STED}}\;=\;775\;\text{nm}$) was operated at an average power of <5 mW at the sample plane, and the standard frame interval was 20 s, comprising ≤10-s acquisition followed by at least ∼10-s recovery, which is compatible with live-cell STED-FLIM imaging (tables S3 and S4) (*65*, *66*). Under repeated imaging without drug treatment, both CONF-FLIM and STED-FLIM showed stable cell-averaged lifetime trajectories, indicating that the standard acquisition alone did not induce comparable lifetime shifts (Fig. 5D). We then used the same framework to monitor stress-associated remodeling after rotenone addition. Under these conditions, STED-FLIM revealed a transient ∼0.2-ns increase in LD-associated lifetime that was not observed in matched CONF-FLIM measurements (Fig. 5E). At the morphological level, mitochondria underwent marked remodeling, progressing toward swelling and rupture (Fig. 5F and movie S5), whereas LDs displayed a staged lifetime trajectory characterized by an initial increase followed by progressive decline (Fig. 5G and fig. S38), revealing a staged microenvironmental response that would be difficult to resolve from static snapshots and consistent with an early adaptive response to acute perturbation. To assess whether phototoxicity alone could explain the transient LD lifetime increase, we deliberately applied a harsher STED regime in the absence of drug treatment using continuous high-power depletion (∼5.25 mW at the sample plane) without recovery intervals (15-s frame interval; 15-s acquisition time). Under these conditions, phototoxicity accumulated and was accompanied by mitochondrial swelling or rupture (fig. S39A); however, LD lifetime did not show excursions comparable to those observed during rotenone challenge (fig. S39B). These findings argue against illumination alone as the source of the transient LD lifetime increase, but they also indicate that STED acquisition can modulate stress responses under rotenone treatment. One plausible explanation is that rotenone places LDs in a stress-sensitized state, after which additional STED exposure accentuates remodeling and microenvironmental change, thereby revealing a transient lifetime increase (*2*, *5*, *67*). More broadly, these results highlight the value of time-lapse imaging for capturing rapid, transient responses that are not resolved by end-point measurements. Overall, these results establish automated sequential STED-FLIM analysis as a practical framework for quantifying stress-associated organelle dynamics and compartment-resolved microenvironmental remodeling in a single-stain workflow.

## DISCUSSION

Live-cell mapping of lipid dynamics across multiple membrane compartments has traditionally relied on organelle-specific labels and multicolor acquisition, often requiring sequential excitation and detection to mitigate spectral cross-talk. These requirements increase experimental complexity, slow acquisition, and can be incompatible with rapid remodeling events. Environment-sensitive lipophilic dyes offer an alternative route: A single probe can reduce labeling burden while reporting local physicochemical properties that vary across membranes and lipid assemblies. NR is a prototypical example, it is weakly emissive in aqueous media but becomes bright in hydrophobic environments, and its emission and lifetime respond to microenvironmental features such as polarity and packing (*48*). By combining these characteristics with STED-FLIM and deep-learning demultiplexing, our work establishes a single-shot strategy for triorganelle imaging along the ER-LD-mitochondria axis.

A key practical advance is that modest modifications of the NR fluorophore tune intracellular partitioning and, thus, the balance of signal contributions from LDs, mitochondria, and ER. Increased lipophilicity enriches neutral lipid staining (LD-biased brightness), whereas modestly increased amphiphilicity promotes broader membrane association and improves visibility of non-LD structures. In our dataset, NR 6CN accentuates LD labeling, whereas hydroxyl-bearing analogs (NR 2OH and NR 3OH) redistribute the signal away from LDs and enhance mitochondrial-associated features. These probe-dependent shifts are operationally important because the ER signal is often comparatively weak, and balanced coverage is needed to interrogate triorganelle remodeling and proximity events that may reflect lipid dynamics.

Fluorescence lifetime provides an orthogonal dimension that strengthens demultiplexing when intensity cues are ambiguous. Across probes, we observe compartment-associated lifetime differences that improve interpretability in crowded regions where morphology and brightness converge. At the same time, lifetime should be interpreted cautiously: Although polarity is a major determinant for NR-class dyes, lifetime in cells can reflect a composite of factors including viscosity, lipid order/packing, refractive index, probe orientation, local concentration, and self-association (*14*, *68*). We therefore interpret lifetime primarily as a microenvironment-sensitive fingerprint that captures remodeling within and across organelles.

Our computational strategy directly addresses a central barrier to single-stain multiplexing: assigning organelle identity despite spectral overlap and intensity heterogeneity. The VGG16-UNet model operates directly on full-frame 1024 by 1024–pixel STED-FLIM images, preserving fine structures that are informative for organelle identity (including ER tubules and mitochondrial ultrastructure) while remaining computationally tractable through a lightweight design. We also identify two practical constraints, annotation quality and class imbalance, especially for small or sparse LD populations, that limit performance and represent clear opportunities for improvement through larger curated datasets, imbalance-aware losses (for example, Dice/focal variants), and refined ground truth generation (including semiautomated annotation with targeted validation) (*69*–*71*). More broadly, the limited availability of benchmarked superresolution organelle datasets remains a field-wide bottleneck. Advanced image restoration models offer one promising direction. For example, models such as LargePNet can improve the continuity and signal-to-noise ratio of weak mitochondrial and ER features, potentially facilitating downstream segmentation (fig. S40) (*72*). However, the broad and heterogeneous staining pattern of NR analogs makes these datasets more complex than conventional single-organelle superresolution images, which may limit direct transfer of restoration models trained under simpler conditions. Future workflows that jointly model restoration, segmentation, and lifetime-aware representation may offer a more general route for extracting weak membrane features from densely labeled superresolution data.

The comparison with phasor-based analysis is also instructive. Phasor methods provide a fitting-free and model-independent view of lifetime distributions and can effectively separate compartments with sufficiently distinct lifetime signatures (*63*, *73*, *74*). Deep learning, by contrast, more effectively incorporates spatial continuity, morphology, and multiscale texture. The two approaches are therefore complementary: Phasor analysis is useful for interrogating mixed populations and lifetime heterogeneity, whereas deep learning excels at preserving structural continuity and spatial integrity. Integrative frameworks that combine these strengths may further improve interpretability and robustness, especially in regions where organelles are closely apposed and probe signals are partially mixed.

Last, these results suggest probe-design principles for next-generation reagents optimized for this framework: moderate amphiphilicity to avoid the collapse of signal into LD cores while maintaining efficient labeling of membranes, strong photostability and depletion tolerance under STED to support time-lapse imaging, and robust lifetime separability across compartments. Together, our approach provides a scalable route to map nanoscale organization and microenvironmental state across the ER-LD-mitochondria axis in living cells, enabling mechanistic studies of organelle interactions, lipid-associated remodeling, and lipid homeostasis under stress.

## MATERIALS AND METHODS

### Polarity sensitivity measurement

NR (Adamas life), NR 2OH (Bidepharm), NR 3OH (Accela ChemBio), and NR 6CN (Achem-block) were prepared as 10 mM stock solutions in DMSO and diluted to 1 μM in the indicated solvents immediately before measurement. Compounds were excited at their absorption maxima (λ_abs_), and fluorescence spectra were acquired on a SHIMADZU RF-6000 spectrometer.

### Water solubility measurement

NR analogs were dissolved in 1 ml of methanol, with the final stock concentration ranging from 1 to 10 mM. The solvent was removed in vacuo. After that, 250 μl of double-distilled water was added. The mixture was vortexed and sonicated in an ultrasonic bath for 45 min at 90°C. The undissolved pellet was separated through centrifugation at 3000*g* for 5 min. Supernatant concentrations were determined by spectroscopy (RF-6000, SHIMADZU).

### Cell culture and transfections

COS-7 cells were obtained from FuHeng Biology Co., Ltd. (Shanghai, China), and maintained in high-glucose Dulbecco’s modified Eagle’s medium (DMEM; Adamas Life) supplemented with 10% fetal bovine serum (Adamas Life) and 1% antibiotic-antimycotic (Adamas Life) at 37°C in a humidified incubator containing 5% (v/v) CO_2_. The imaging validation of other cell lines (HeLa, U251, and GL261) follows the same culture method. For imaging experiments, cells were seeded onto one-chamber or four-chamber glass-bottom dishes (Biosharp) and cultured for 24 hours before staining or transfection. For TOM20 labeling, COS-7 cells were transfected with plasmid DNA encoding TOM20-Halo using Hieff Trans Liposomal Transfection Reagent (Yeasen Biotechnology) according to the standard procedure. Briefly, 0.5 μg of plasmid DNA and 1.2 μl of transfection reagent were diluted in 50 μl of DMEM, incubated for 20 min at room temperature, and added to each well of a 20-mm four-chamber glass-bottom dish.

### Cell viability assays

The cytocompatibility of the NR analogs was evaluated using a standard MTT [3-(4,5-dimethylthiazol-2-yl)-2,5-diphenyltetrazolium bromide] assay. COS-7 cells were seeded in 96-well plates (1 × 10^4^ cells per well) and cultured at 37°C in a 5% CO_2_ incubator for 24 hours. The culture medium was then replaced with a fresh culture medium containing the indicated probe at final concentrations of 0.5, 1, or 5 μM. After 6 hours of incubation, 20 μl of MTT solution (20 μl per well, 5 mg/ml) was added to each well, cells were incubated for an additional 4 hours, the solution was then replaced with DMSO (200 μl per well), and the samples were quantified using a microplate reader. Absorbance was measured using a microplate reader, and cell viability was calculated relative to the untreated control group.

### CONF imaging and colocalization analysis

For CONF imaging, COS-7 cells were cultured on glass-bottom dishes for 24 hours before staining. For organelle colocalization experiments, cells were incubated with 1 μM BODIPY 493/503 (Beyotime), 500 nM MitoTracker Green (MT-Green, Beyotime), or 500 nM ER-Tracker Blue-White DPX (ER-DPX, Yeasen Biotechnology) for 30 min at 37°C. The staining medium was then replaced with fresh DMEM, and cells were subsequently incubated with 500 nM NR, NR 2OH, NR 3OH, or NR 6CN for 5 min before imaging. CONF images were acquired on a Leica TCS SP8 STED 3× microscopy system equipped with a 100×/1.40 oil-immersion objective. Excitation was provided by a white-light laser set to 485 nm for BODIPY 493/503, 490 nm for MT-Green, and 405 nm for ER-DPX. Emission was collected at 495 to 550 nm, 500 to 550 nm, and 440 to 600 nm, respectively. Whole-cell microviscosity was assessed with CCVJ (Adamas) after 30 min of incubation, and membrane lipid order was assessed with Laurdan (TargetMol) after 30 min of incubation (*59*, *60*). Laurdan was excited at 405 nm and CCVJ at 450 nm.

Colocalization was quantified in Fiji/ImageJ. In addition to qualitative inspection, PCC was calculated for the indicated probe-marker pairs using identical thresholding criteria within each experiment.

### Live-cell STED imaging and STED-FLIM imaging

For live-cell STED and STED-FLIM imaging, COS-7 cells were seeded on one-chamber or four-chamber glass-bottom dishes and cultured for 24 hours. Cells were then incubated in DMEM containing 0.5 μM NR, NR 2OH, NR 3OH, or NR 6CN for ∼10 min at 37°C and imaged directly in the staining medium without washing. STED-FLIM imaging was performed on a Leica TCS SP8 STED 3× system equipped with two continuous-wave depletion lasers (592 and 660 nm) and a pulsed 775-nm depletion laser with a 600-ps pulse width at 80 MHz. Fluorescence was detected using a hybrid detector (HyD), and images were acquired with a STED WHITE objective (100×/1.40 oil-immersion). Unless otherwise stated, excitation was performed at 550 nm for NR, NR 2OH, and NR 3OH and at 585 nm for NR 6CN. Emission was collected at 565 to 700 nm for NR 2OH and NR 3OH and at 595 to 700 nm for NR 6CN. For orthogonal validation under live-cell superresolution conditions, cells were incubated with 500 nM HBmito Crimson (Genvivo) for 30 min or with 200 nM Janelia Fluor 646 (JF646) HaloTag ligand (Yeasen Biotechnology) for 1 hour and imaged directly without washing (*53*, *54*). These channels were excited at 655 and 645 nm, respectively, and emission was collected at 665 to 720 nm and 655 to 710 nm, respectively. The 775-nm pulsed laser was used for depletion in both channels.

Unless otherwise noted, STED images were acquired at a pixel size of 22.4 nm by 22.4 nm or smaller, with scan speeds of 200 or 400 Hz (pixel dwell time of 3.85 or 1.41 μs) and line averaging of 2 or 3. Nominal laser settings were 1 to 5% for excitation and 50 to 55% for the 775-nm STED laser, corresponding to an average power of ∼4.19 to 4.68 mW at the sample plane. For time-lapse STED imaging, the standard frame interval was 20 s, typically comprising ≤10-s acquisition followed by ∼10-s recovery. All acquisition parameters were kept constant within each experimental comparison. Raw images were processed in Fiji/ImageJ. Minor linear adjustments of brightness and contrast were applied only for figure display and were performed identically across matched image sets.

Additional superresolution imaging was performed on an Abberior Facility Line 3D microscopy system equipped with an Olympus UPlanSApo 100×/1.40 oil-immersion objective. Nominal laser settings were 5% for 561-nm excitation and 45% for the 775-nm STED beam (maximum output power, 1.2 W). The pixel size was set to 60 nm or higher for CONF and nondiffracted acquisitions. Minor contrast/brightness adjustments and deconvolution were performed using Imspector software (Abbey instrument).

### Fluorescence lifetime analysis of solution

Fluorescence decay curves of probes in solution were analyzed using EzTime software (HORIBA), which is compatible with the DeltaFlex platform. Decays were fit using *N*-component multiexponential models convolved with the instrument response function.

### Model architecture, dataset annotation, and training

Organelle segmentation was implemented in Python using TensorFlow in the PyCharm environment. The model was based on a VGG16-UNet architecture, in which a pretrained VGG16 encoder was combined with a symmetric decoder for pixelwise prediction. The model took 2D STED-FLIM RGB images (1024 by 1024 pixels) as input and generated multiclass segmentation masks for mitochondria, LDs, and ER.

The dataset comprised 75 STED-FLIM images acquired under representative imaging conditions. Given the limited dataset size, data augmentation was applied to expand the training data and improve model robustness. Augmentation was performed on the fly and included random horizontal and vertical flipping, 90° rotations, and modest adjustments in image brightness and contrast. Model performance was assessed by fivefold cross-validation, with a 4/1 split between training and validation data in each fold.

Ground truth masks were generated manually in Fiji/ImageJ. Annotation criteria were first established using correlative costaining with commercial organelle markers, including BODIPY 493/503 for LDs, MitoTracker Green for mitochondria, and ER-DPX for the ER, together with the corresponding NR analog signals. These experiments were used to define organelle-specific imaging signatures on the basis of morphology, intensity distribution, and fluorescence lifetime. Subsequent manual annotations were generated according to these marker-informed criteria.

The model was trained using an Adam optimizer with a binary cross-entropy loss function. Training was performed with an initial learning rate of 0.0001, which was reduced by a factor of 0.5 if the validation loss plateaued for 10 consecutive epochs. Training was conducted using a batch size of 4 for up to 200 epochs on an NVIDIA GeForce RTX 3080 Ti GPU. Early stopping was applied if the validation Dice score did not improve for 30 consecutive epochs, and the final model weights were selected from the epoch with the best validation performance.

### Image analysis

Fluorescence intensity analysis was performed in Fiji/ImageJ. The colocalization coefficient was obtained via the “colocalization finder” plug-in in ImageJ. For compartment-resolved analysis, regions of interest were defined either manually or from model-generated masks, as specified in the corresponding figure legends. Fluorescence intensity trajectories and summary plots were generated in OriginPro 2026. Unless otherwise stated, all image-processing operations used identical parameter settings within each experimental comparison.

### Statistical analysis

Statistical analysis was performed using OriginPro 2026. Data are presented as the means ± SD, unless otherwise specified in the figure legends. Comparisons between two independent groups were performed using an independent two-tailed Student’s *t* test. A *P* value of less than 0.05 was considered statistically significant.
