# Supplementary material for: Deep learning–enhanced single-shot triorganelle STED-FLIM imaging of lipid dynamics in living cells
Source: Sci Adv. 2026 Sep 30;12(40):eaeh3416. doi: 10.1126/sciadv.aeh3416 (PMC13626041; doi:10.1126/sciadv.aeh3416)
Supplement: Supplementary file 1 [file sciadv.aeh3416_sm.pdf]

Science Advances
AAAS

Supplementary Materials for

Deep learning–enhanced single-shot triorganelle STED-FLIM imaging of lipid dynamics in living cells

Lu Gao *et al.*

Corresponding author: Fu Wang, wangfu@sjtu.edu.cn; Li Jiang, jiang.li@ecust.edu.cn

Sci. Adv. **12**, eaeh3416 (2026)
DOI: 10.1126/sciadv.aeh3416

The PDF file includes:

Supplementary Methods
Figs. S1 to S40
Tables S1 to S4
Legends for movies S1 to S5

Other Supplementary Material for this manuscript includes the following:

Movies S1 to S5

Methods

FLIM analysis of cellular images

FLIM images were analyzed in LAS X FLIM software (Leica Microsystems). A separate empirical measurement of the instrument response function (IRF) was not available under the Leica STED-FLIM configuration used in this study. Therefore, fluorescence decays were fit in LAS X using the vendor-implemented reconvolution framework, in which the measured decay is modeled as the convolution of the system response with one or more exponential decay components.

For a multiexponential decay, the fitted signal can be written as:

$$y(t) = \{IRF(t + Shift_{IRF}) + Bkgr_{IRF}\} \otimes \left\{\sum_{i=0}^{n-1} A[i] e^{\left(-\frac{t}{\tau[i]}\right)} + Bkgr\right\} \quad \text{(S1)}$$

In Eq. S1, IRF denotes the effective system response function incorporated by the software, n denotes number of exponential components, A denotes amplitudes-exponential pre-factors, τ denotes Exponential Decay Times (e.g. lifetimes), Bkgr denotes tail offset-correction for background (afterpulsing, dark counts, environmental light), $Shift_{\mathrm{IRF}}$ denotes IRF Shift-correction for IRF displacement (e.g. wavelength dependent zero time of the detector), and $Bkgr_{\mathrm{IRF}}$ denotes IRF background-correction for IRF background.

The mean fluorescence lifetime reported in this study was the intensity-weighted lifetime:

$$\tau_{Av\ Int} = \frac{\sum_{k=0}^{n-1} I[k]\tau[k]}{I_{Sum}} \quad \text{(S2)}$$

This quantity was used as the representative lifetime value for each image or region of interest. Pixelwise fitting was performed by maximum-likelihood estimation. Depending on photon statistics and decay complexity, mono-, bi-, or tri-exponential reconvolution models were applied. Fits were accepted when the χ^2 was <4 for global fitting (owing to pixel heterogeneity) and <1.3 for region of interest fitting.

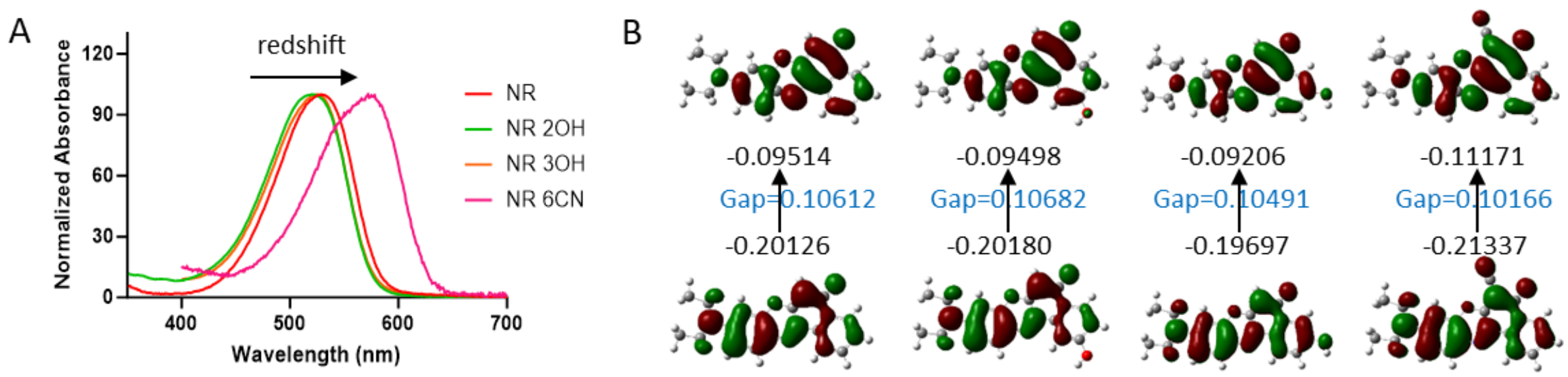

Fig. S1. Photophysical properties of the NR analogs. (A) Absorption spectra of NR and its analogs in tetrahydrofuran. (B) DFT calculation of NR analogs.

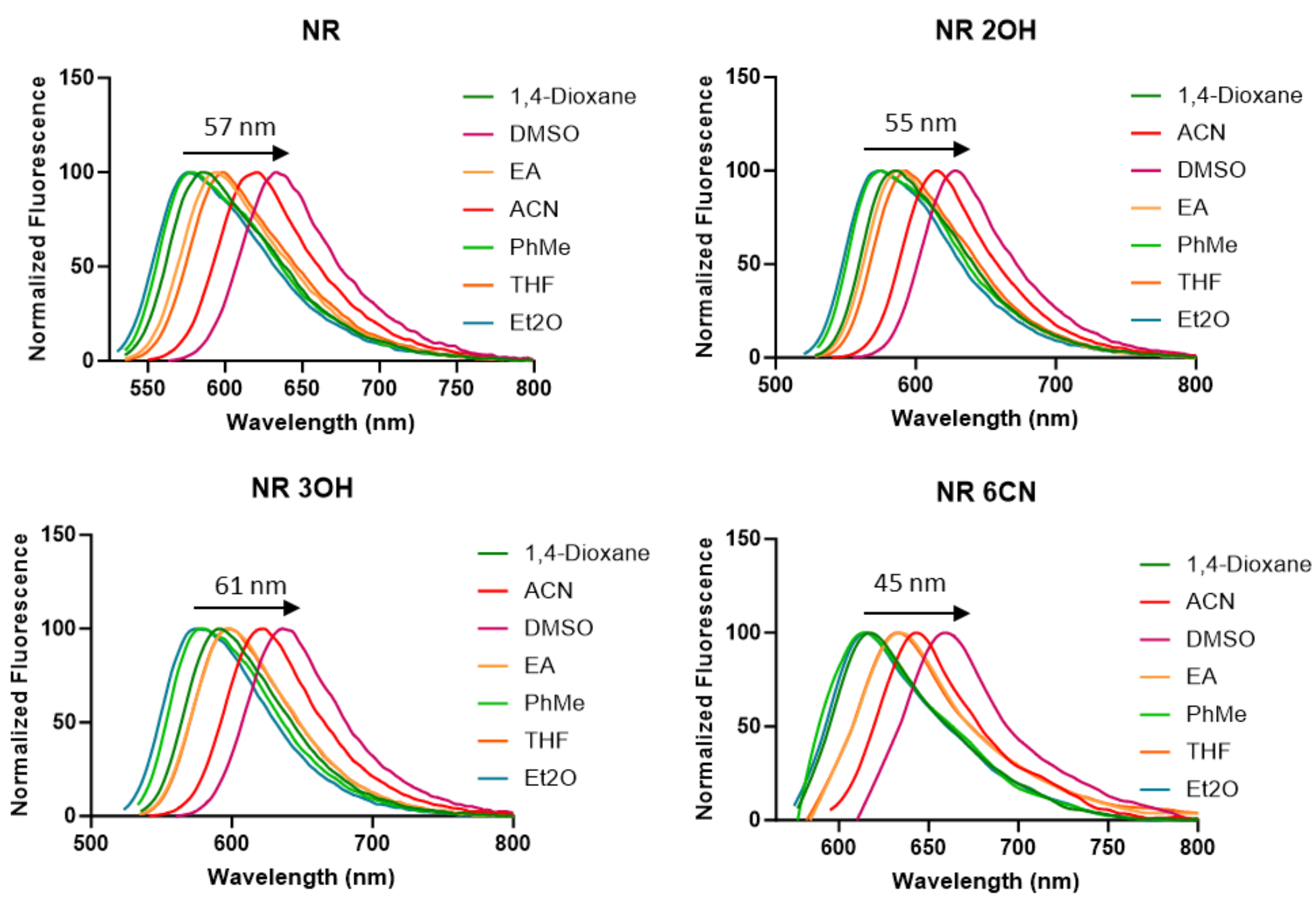

Fig. S2. Fluorescence emission of the NR analogs in solvents of different polarity. Fluorescence emission spectra of the NR analogs in 1,4-Dioxane, Dimethyl sulfoxide (DMSO), ethyl acetate (EA), toluene (PhMe), tetrahydrofuran (THF), diethyl ether (Et2O), and acetonitrile (ACN).

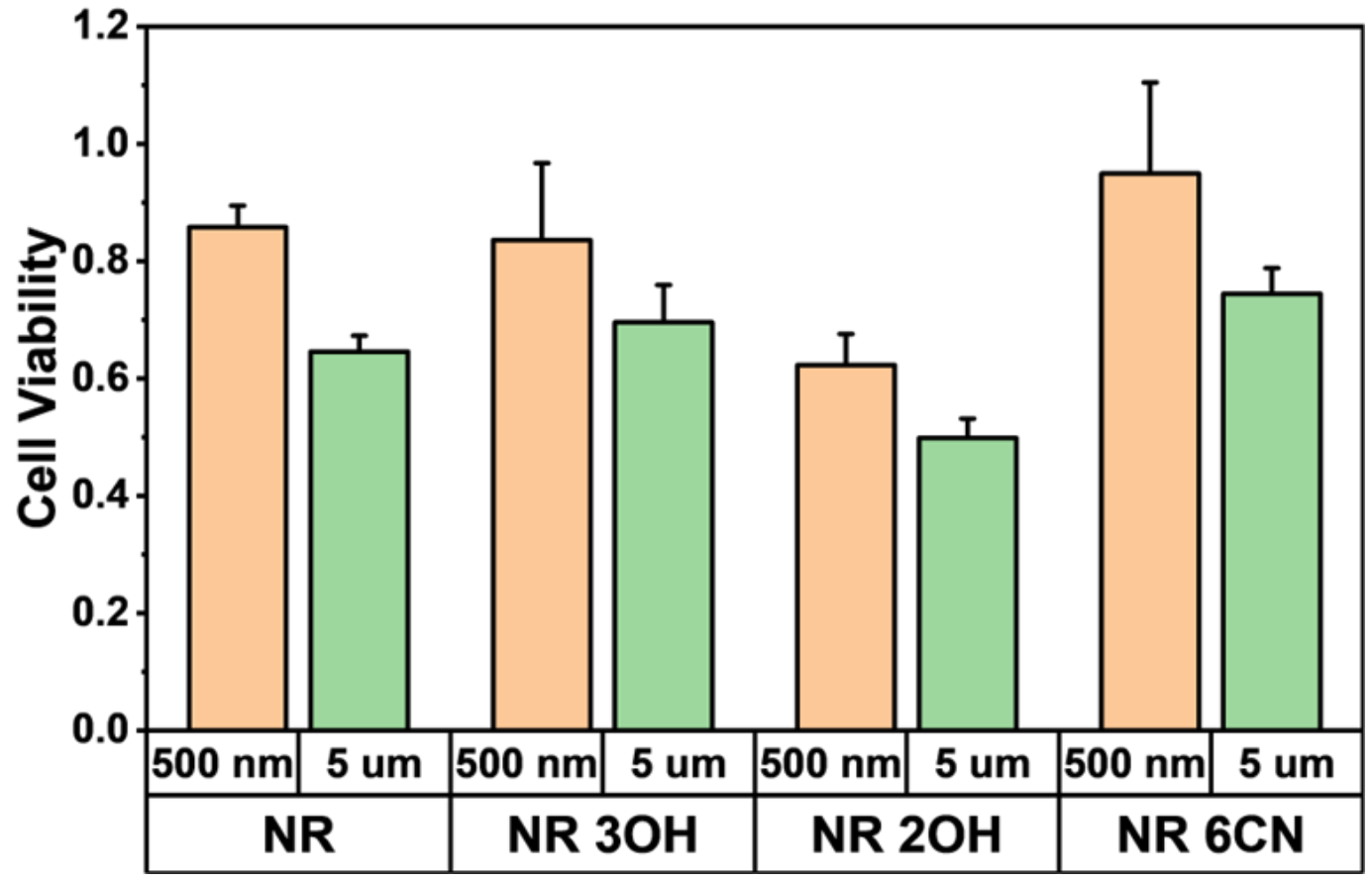

Fig. S3. Cytocompatibility of the NR analogs in COS-7 cells. MTT assays of COS-7 cells incubated with different concentrations of the NR analogs for 6 h. Data are mean ± s.d. from six biologically independent replicates.

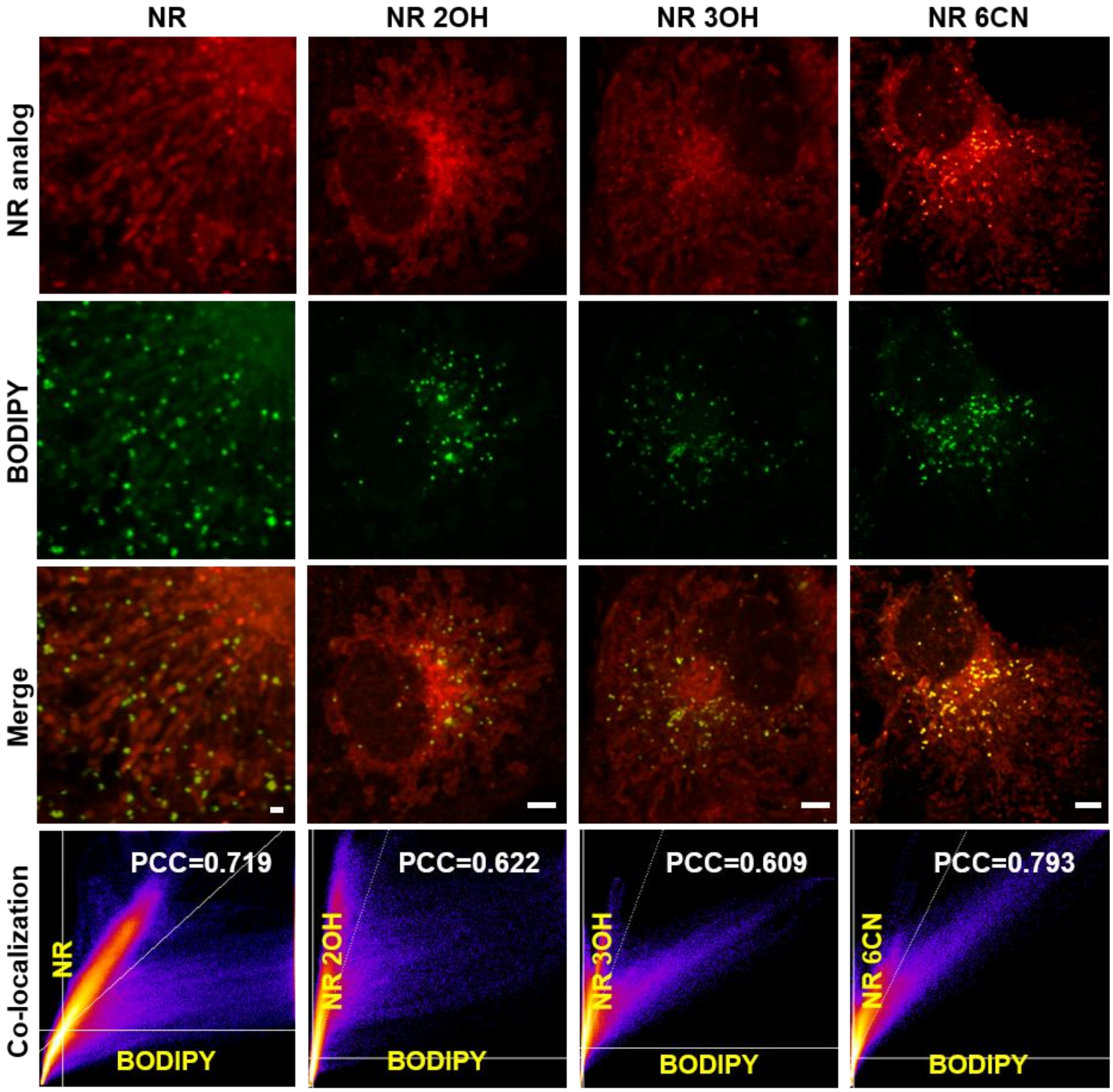

Fig. S4. Colocalization of NR analogs with lipid droplets in COS-7 cells. COS-7 cells were co-stained with BODIPY 493/503 and the indicated NR analogs. The bottom row shows scatter plots and the corresponding Pearson correlation coefficients (PCCs). Scale bars, 5 μm.

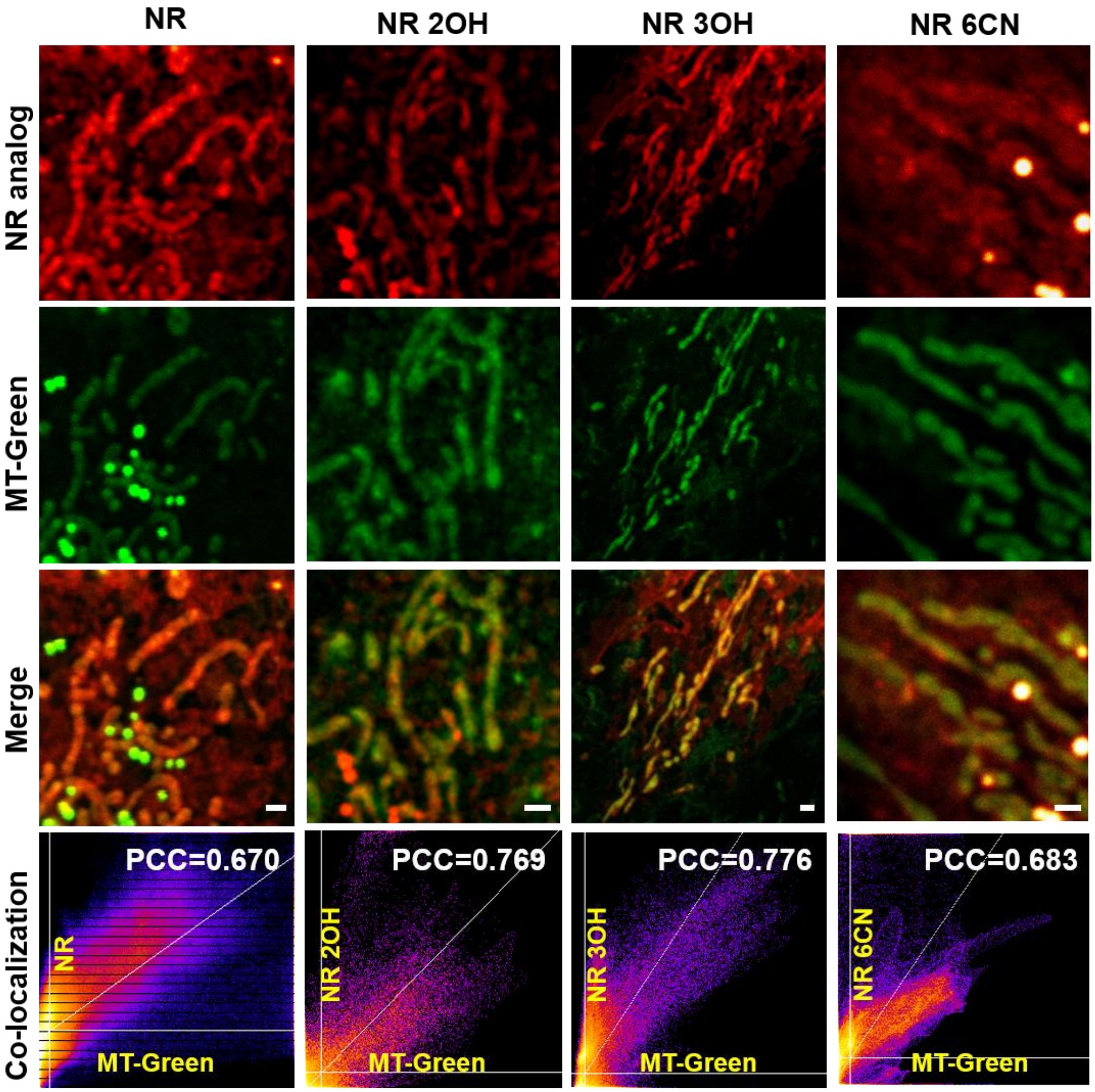

Fig. S5. Colocalization of NR analogs with mitochondria in COS-7 cells. COS-7 cells were co-stained with MitoTracker Green (MT-Green) and the indicated NR analogs. The bottom row shows scatter plots and the corresponding PCCs. Scale bars, 2 μm.

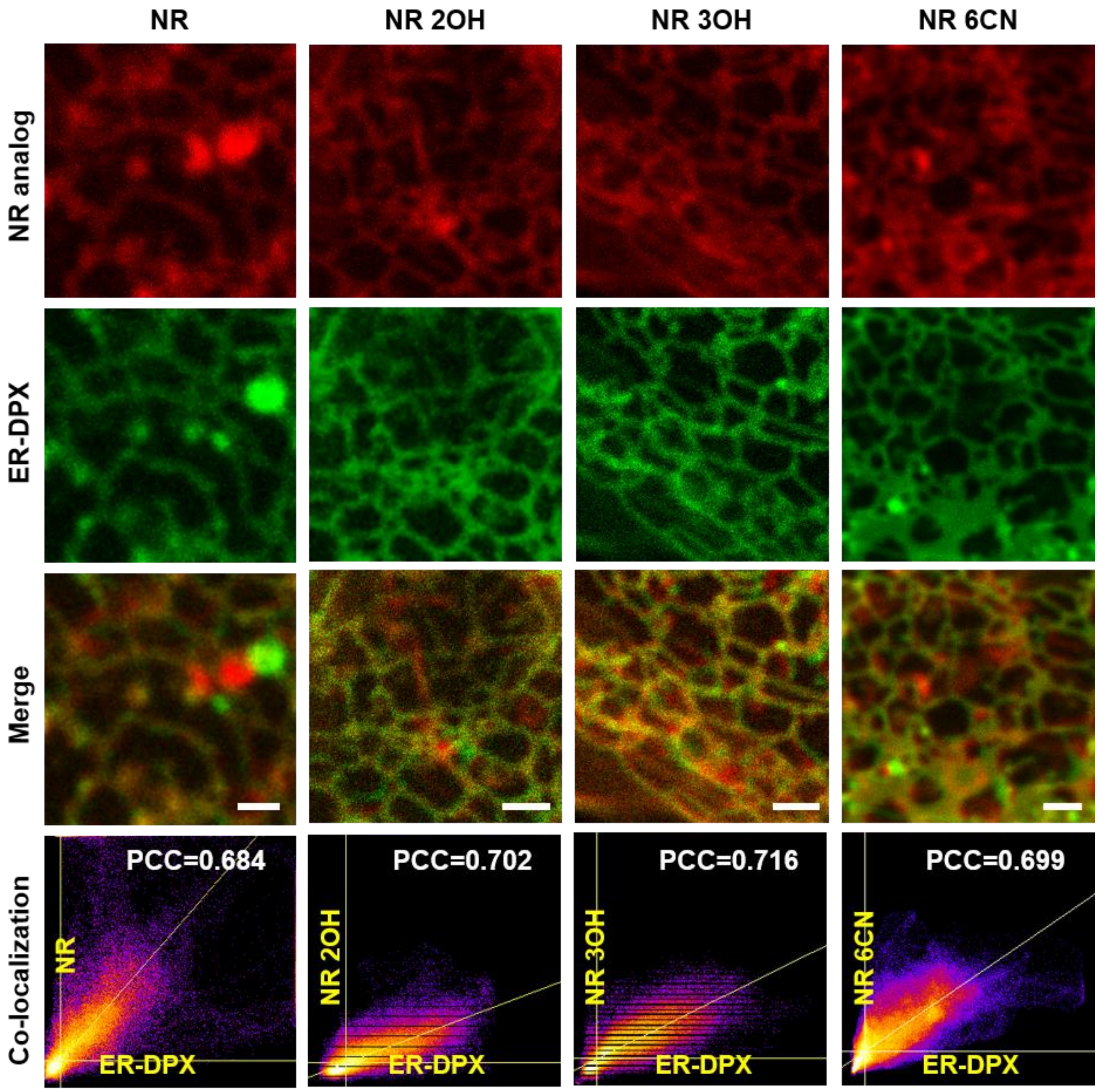

Fig. S6. Colocalization of NR analogs with the endoplasmic reticulum in COS-7 cells. COS-7 cells were co-stained with ER-Tracker Blue-White DPX (ER-DPX) and the indicated NR analogs. The bottom row shows scatter plots and the corresponding PCCs. Scale bars, 1 μm.

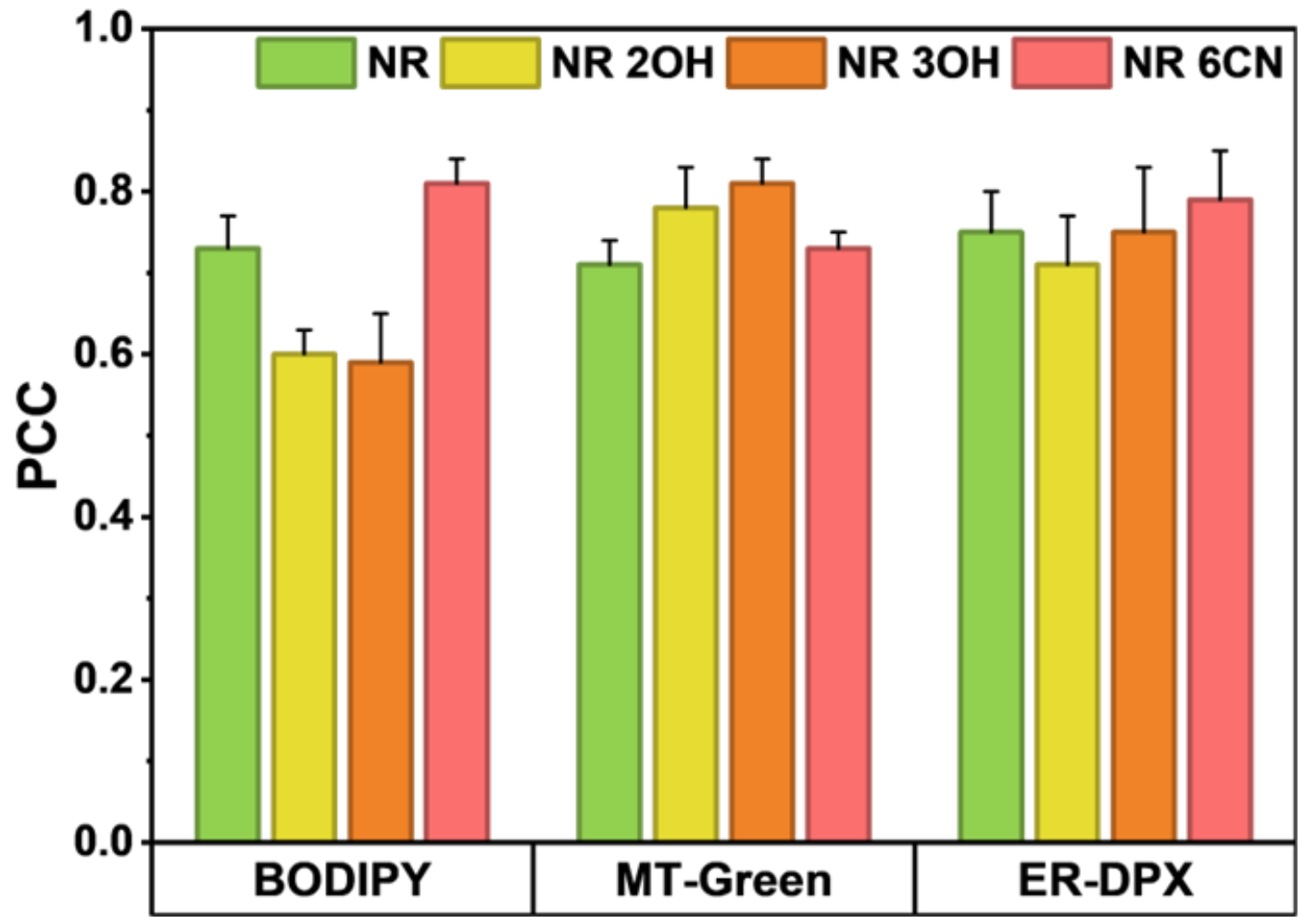

Fig. S7. Statistical summary of co-localization between NR analogs and organelle markers. PCC between the NR analogs and organelle markers for LDs (BODIPY 493/503), mitochondria (MT Green), and ER (ER-DPX). Data are mean ± s.d. (n ≥ 20 cells per analog).

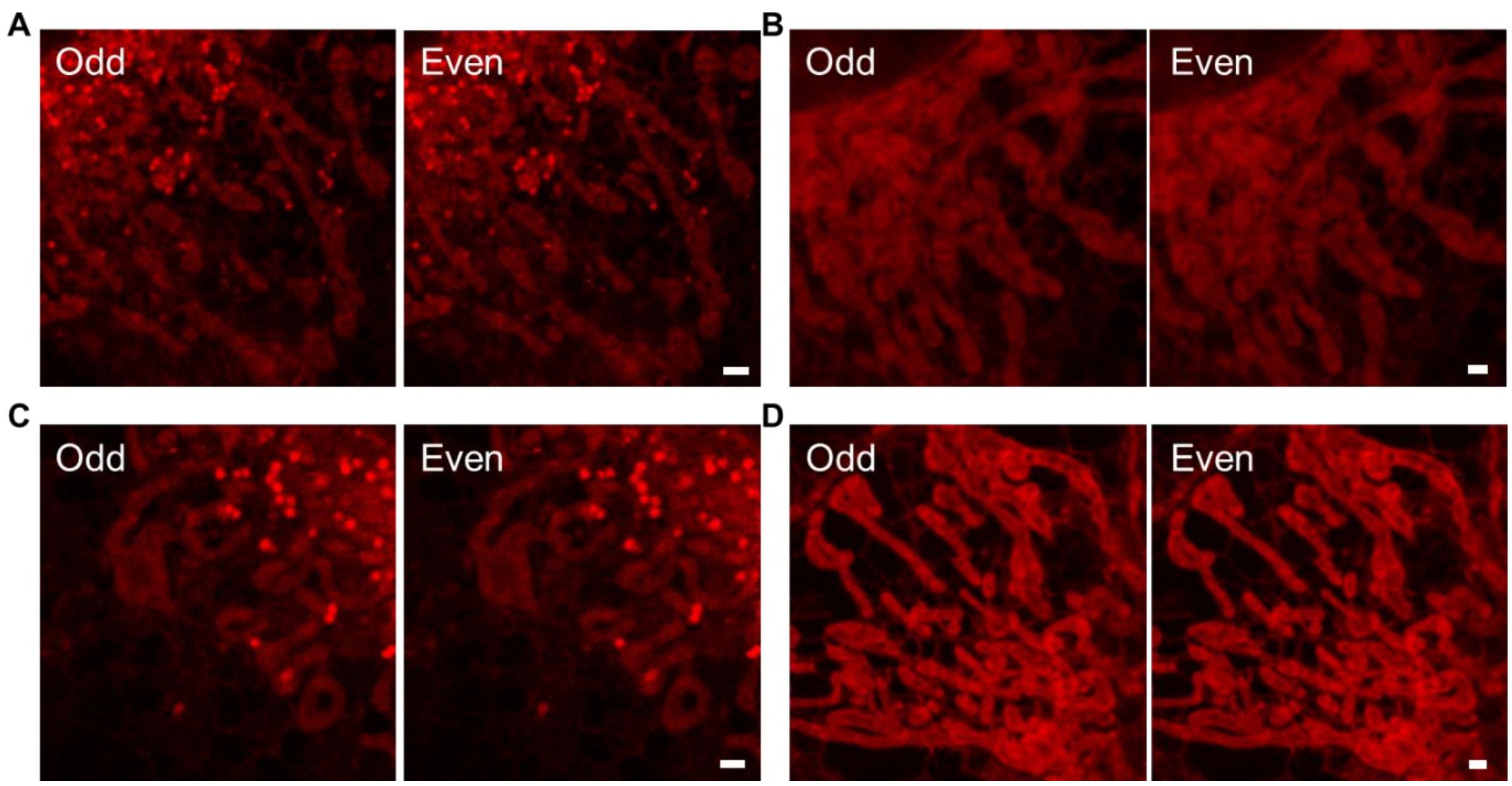

Fig. S8. FRC analysis based on time-resolved STED raw data. Two statistically independent images were reconstructed from odd and even frames of time-resolved STED raw data. (A) to (D) show COS-7 cells stained with NR, NR 2OH, NR 3OH, and NR 6CN, respectively. Scale bars, 2 µm.

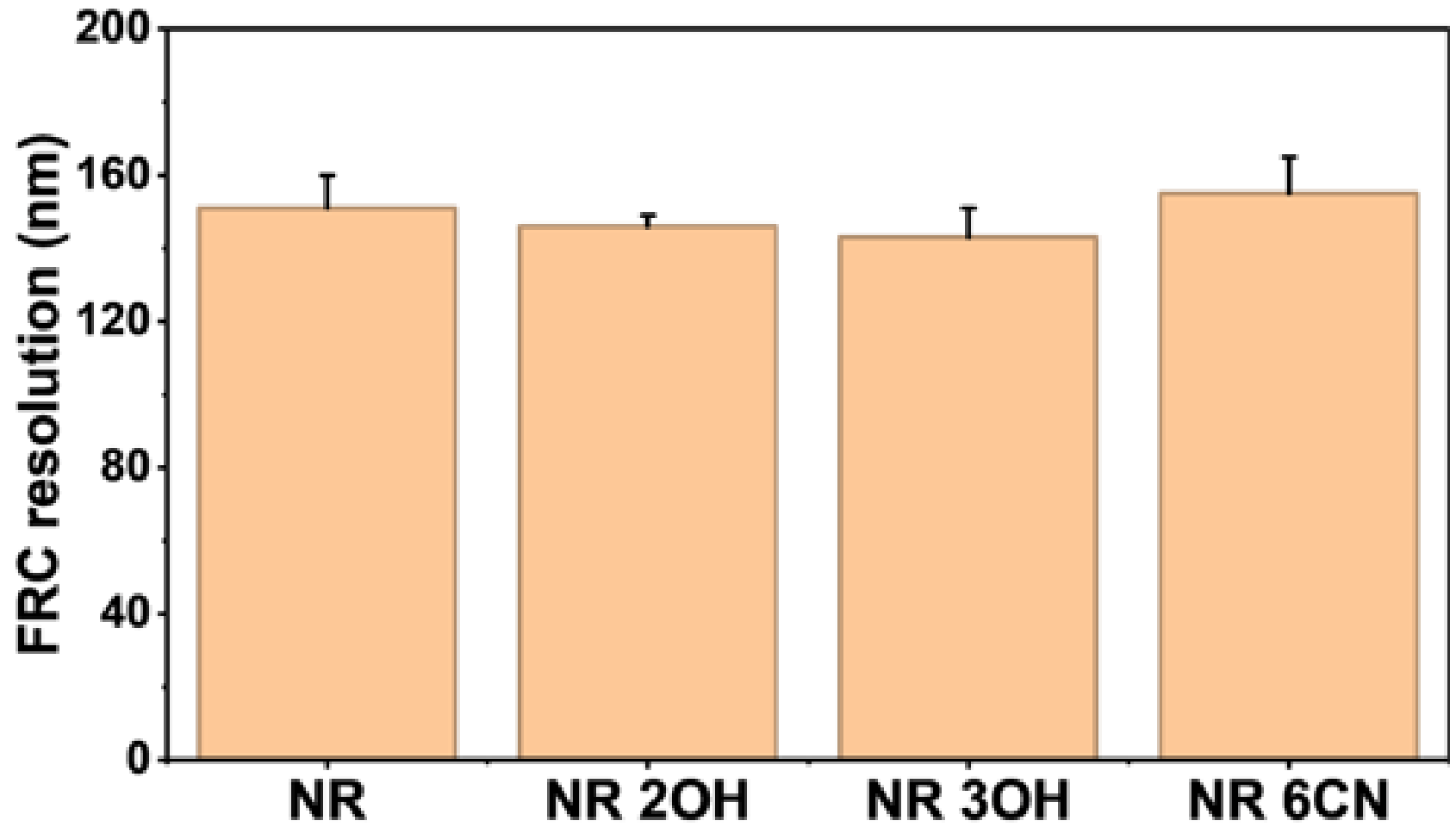

Fig. S9. Statistical summary of FRC resolution for NR-analog STED imaging. The STED images used for FRC calculation were obtained from COS-7 cells stained with NR, NR 2OH, NR 3OH, and NR 6CN, respectively. Data are mean ± s.d. ($n \geq 25$ images per analog).

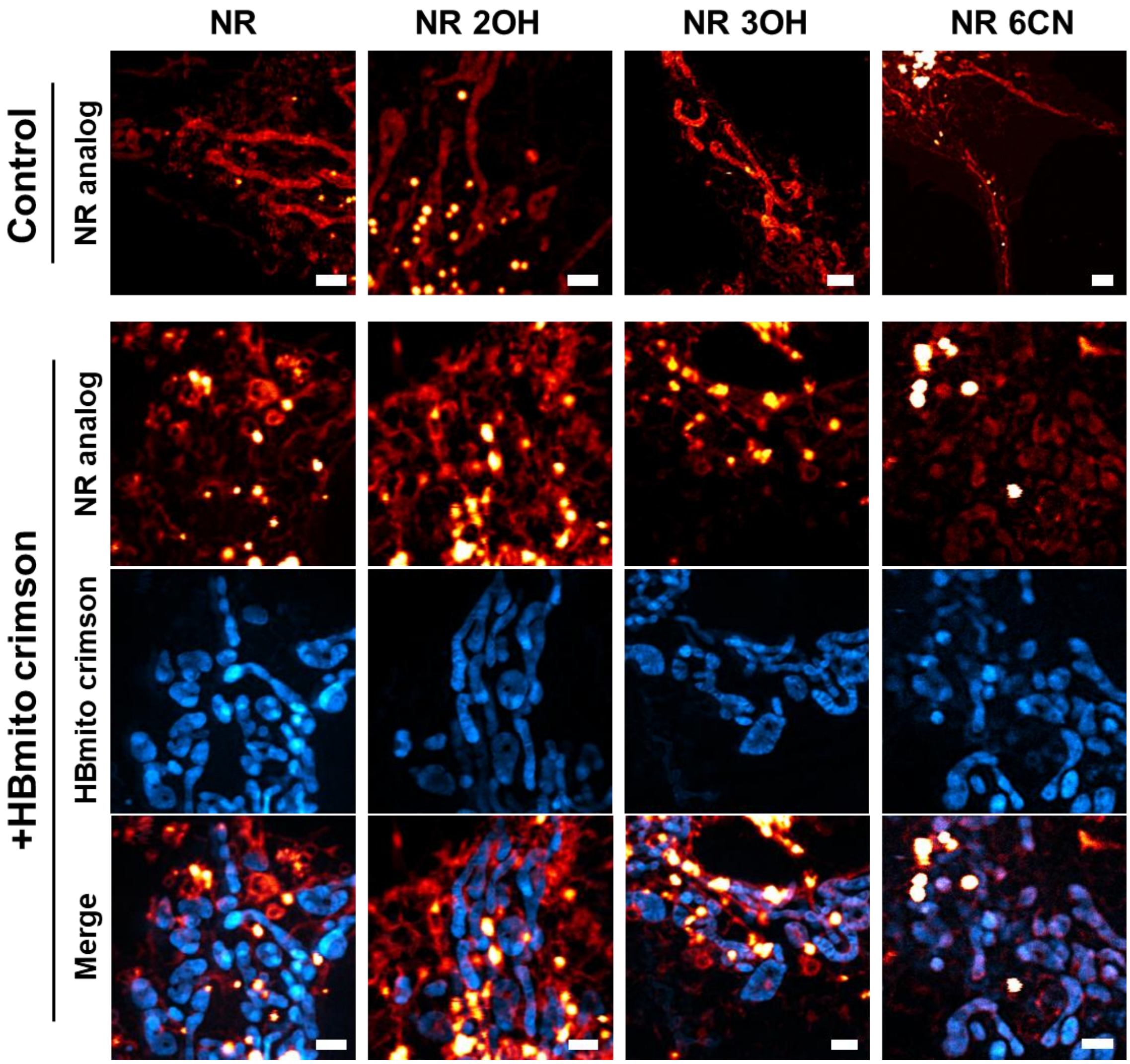

Fig. S10. Co-localization of NR analogs with an inner mitochondrial membrane marker. Co-localization STED imaging of NR analogs with the inner mitochondrial membrane marker HBmito Crimson. Cells stained with NR analogs alone were used as controls. Scale bars, 2 μm.

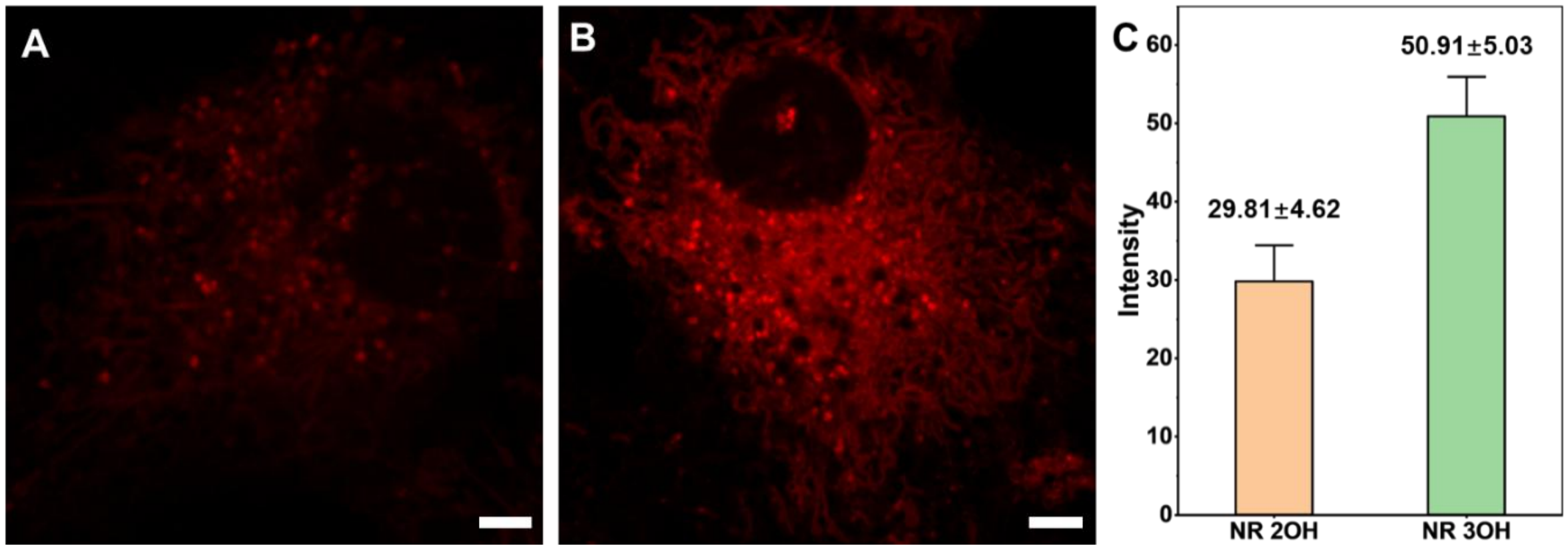

Fig. S11. Comparison of cellular fluorescence intensity between NR 2OH and NR 3OH. (A and B) Representative fluorescence images of COS-7 cells stained with NR 2OH or NR 3OH. Scale bars, 5 μm. (C) Mean cellular fluorescence intensities of COS-7 cells stained with NR 2OH (500 nM) or NR 3OH (500 nM) under identical excitation conditions (550 nm, 2% laser power). Data are mean ± s.d. (n ≥ 20 images per analog).

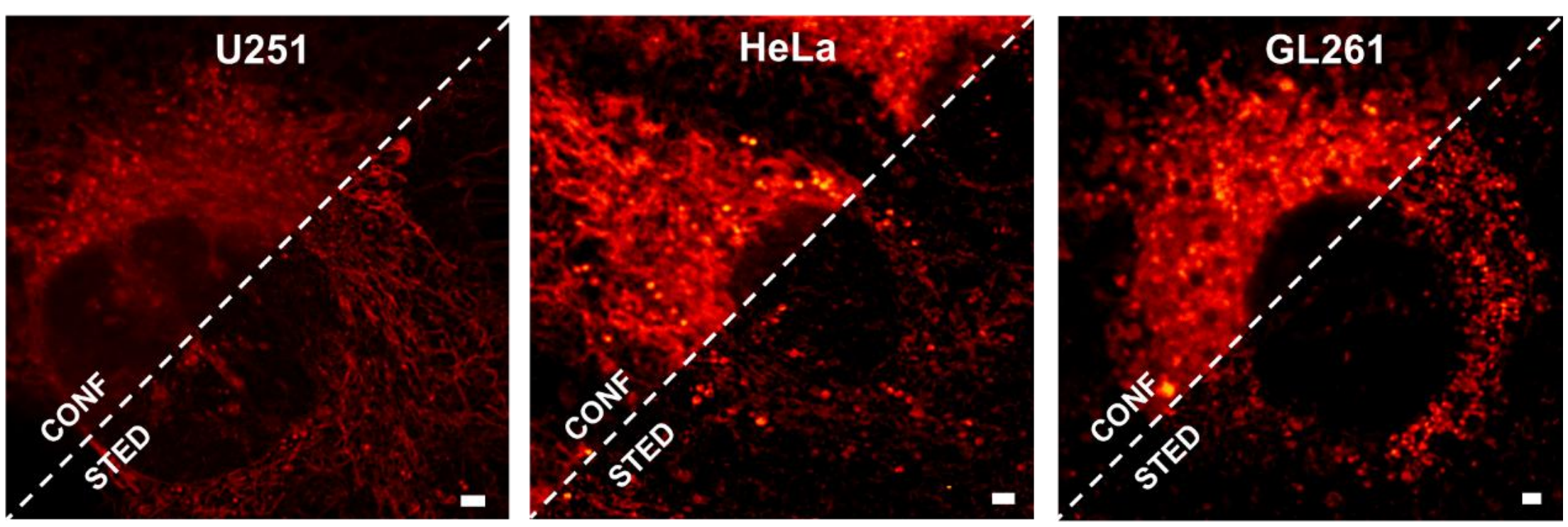

Fig. S12. Imaging performance of NR 3OH in different cell lines. Representative confocal and STED images of U251, HeLa, and GL261 cells stained with 500 nM NR 3OH. Scale bars, 2 μm.

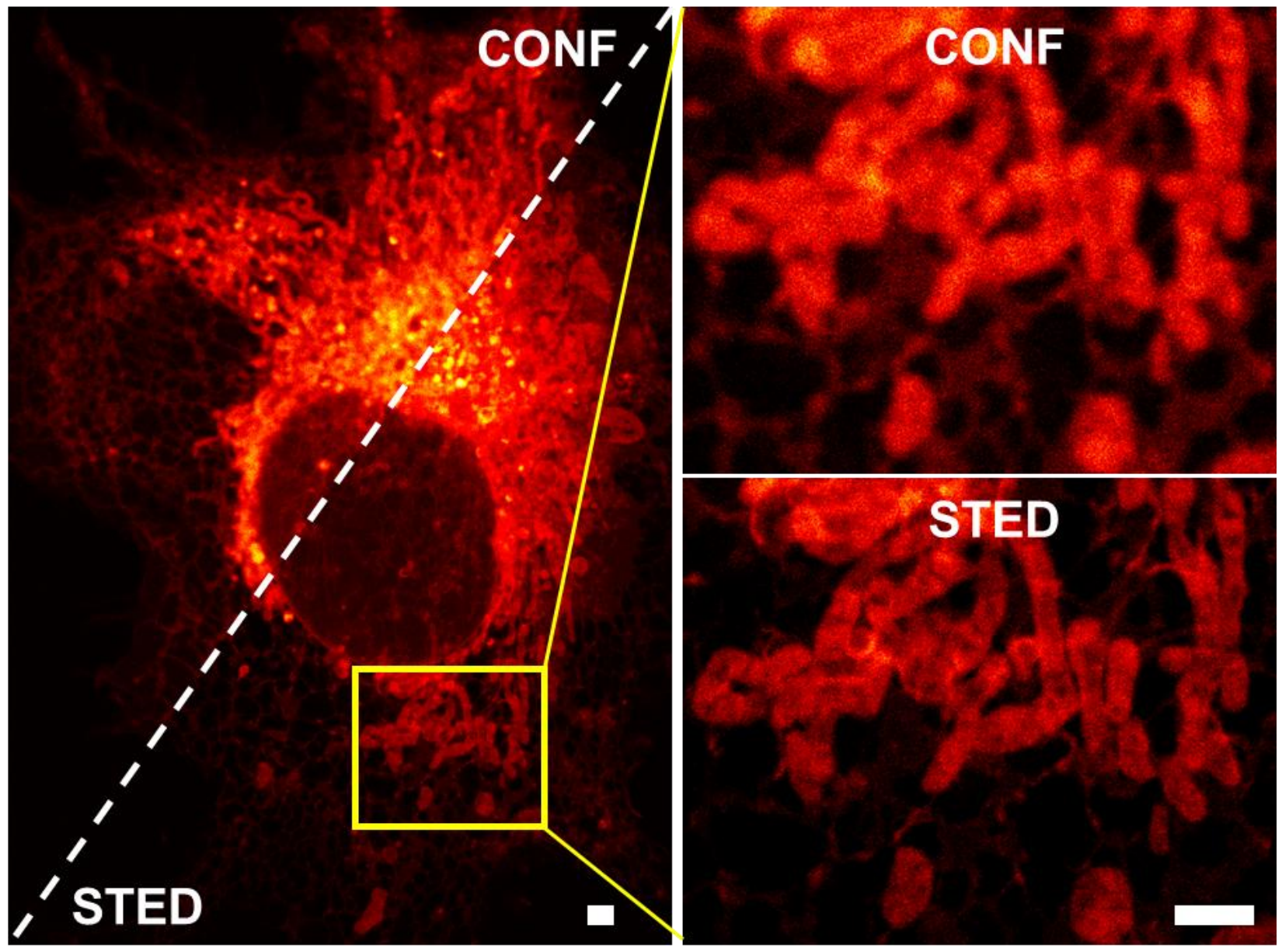

Fig. S13. Live-cell confocal and STED imaging of NR 3OH on an Aberrior microscope. Representative live-cell confocal and STED imaging of COS-7 cells stained with NR 3OH on an Aberrior STED microscope. Scale bars, 2 µm.

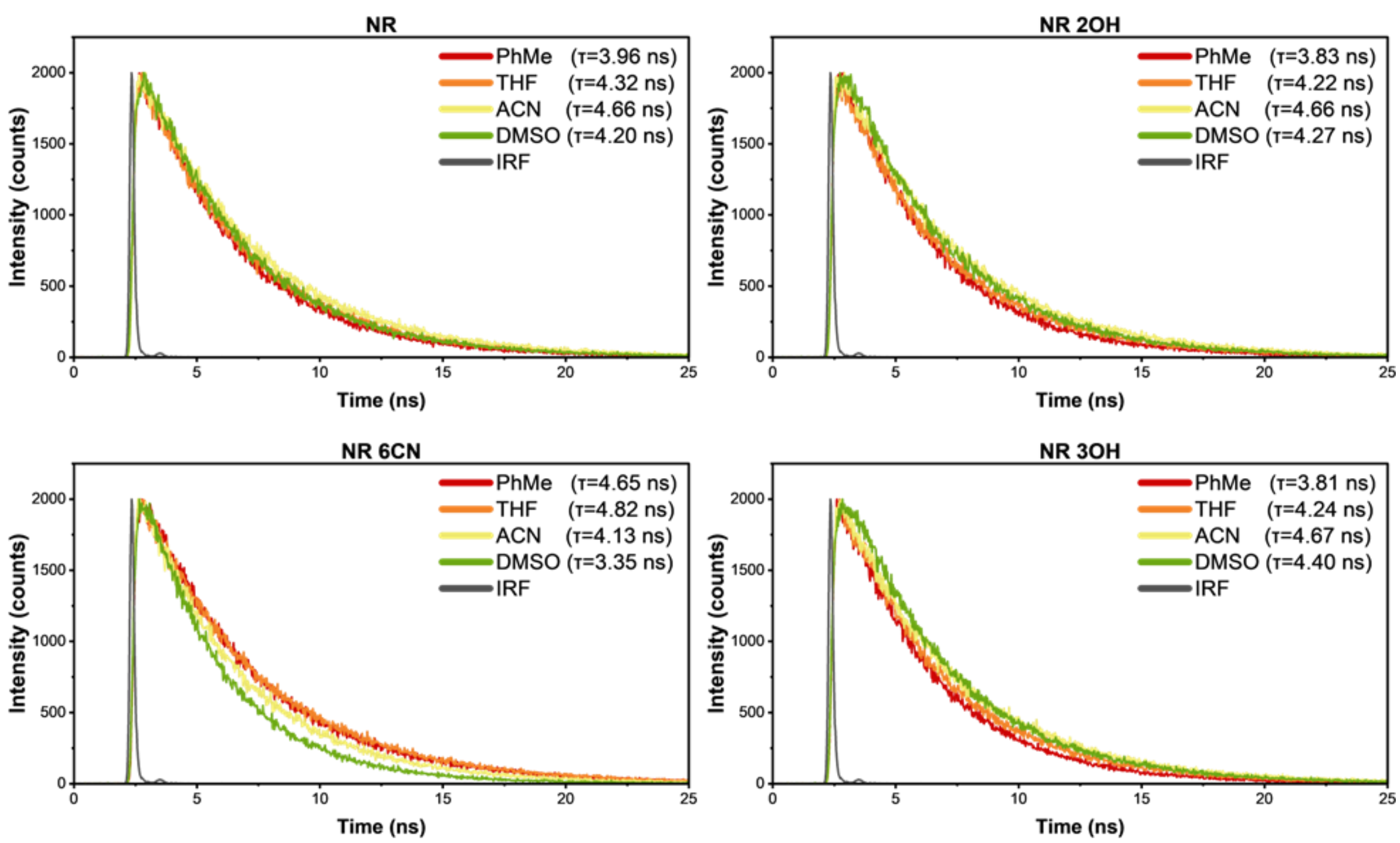

Fig. S14. Fluorescence lifetimes of the NR analogs in solvents of different polarity. Representative lifetime measurements in toluene (PhMe), tetrahydrofuran (THF), acetonitrile (ACN), and DMSO. IRF, instrument response function.

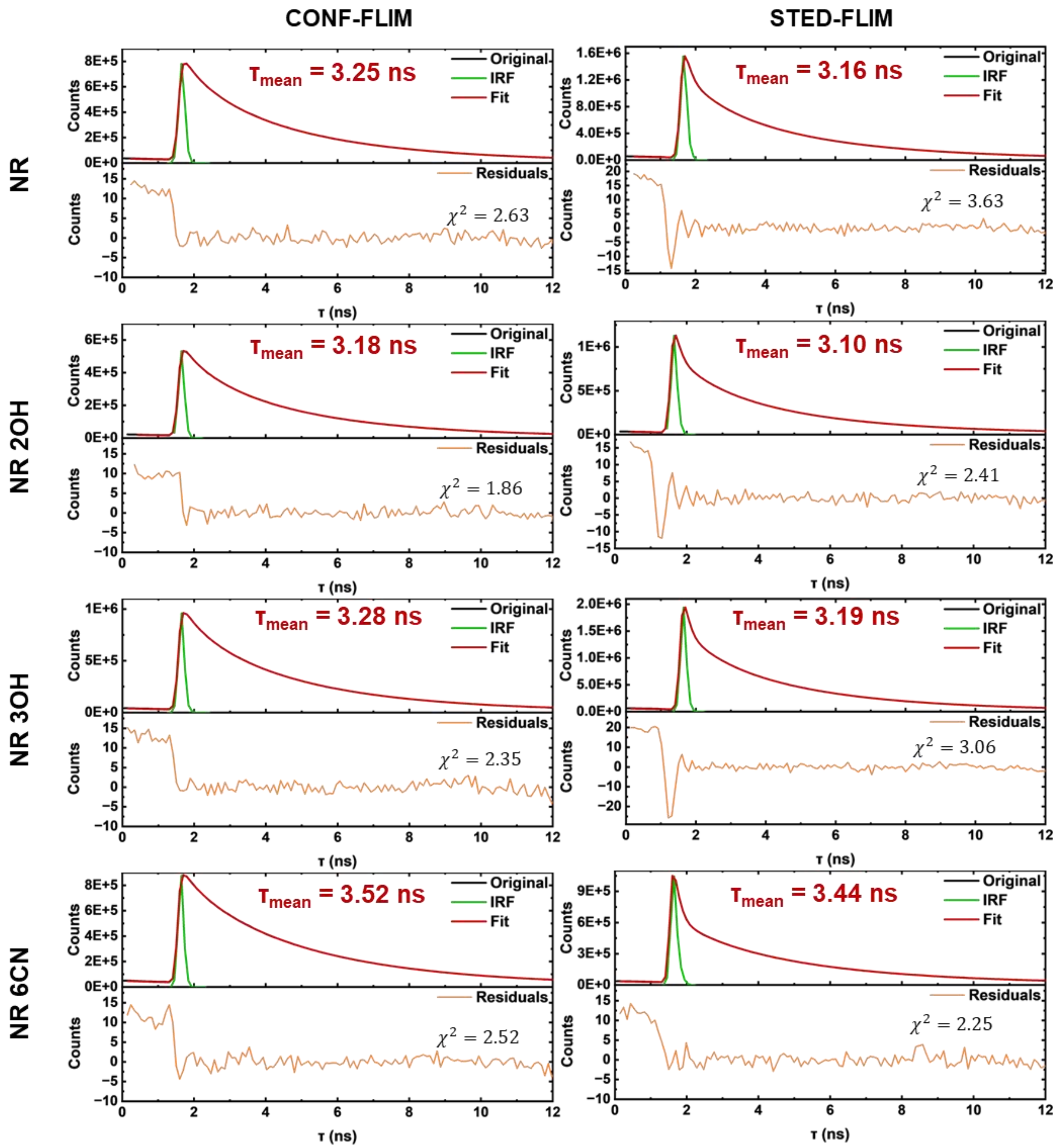

Fig. S15. Fluorescence lifetime fitting of COS-7 cells stained with the NR analogs. Fluorescence lifetime curves of COS-7 cells stained with NR, NR 2OH, NR 3OH, and NR 6CN from the same field of view in confocal and STED imaging modes, fitted in LAS X FLIM software using tri-exponential reconvolution.

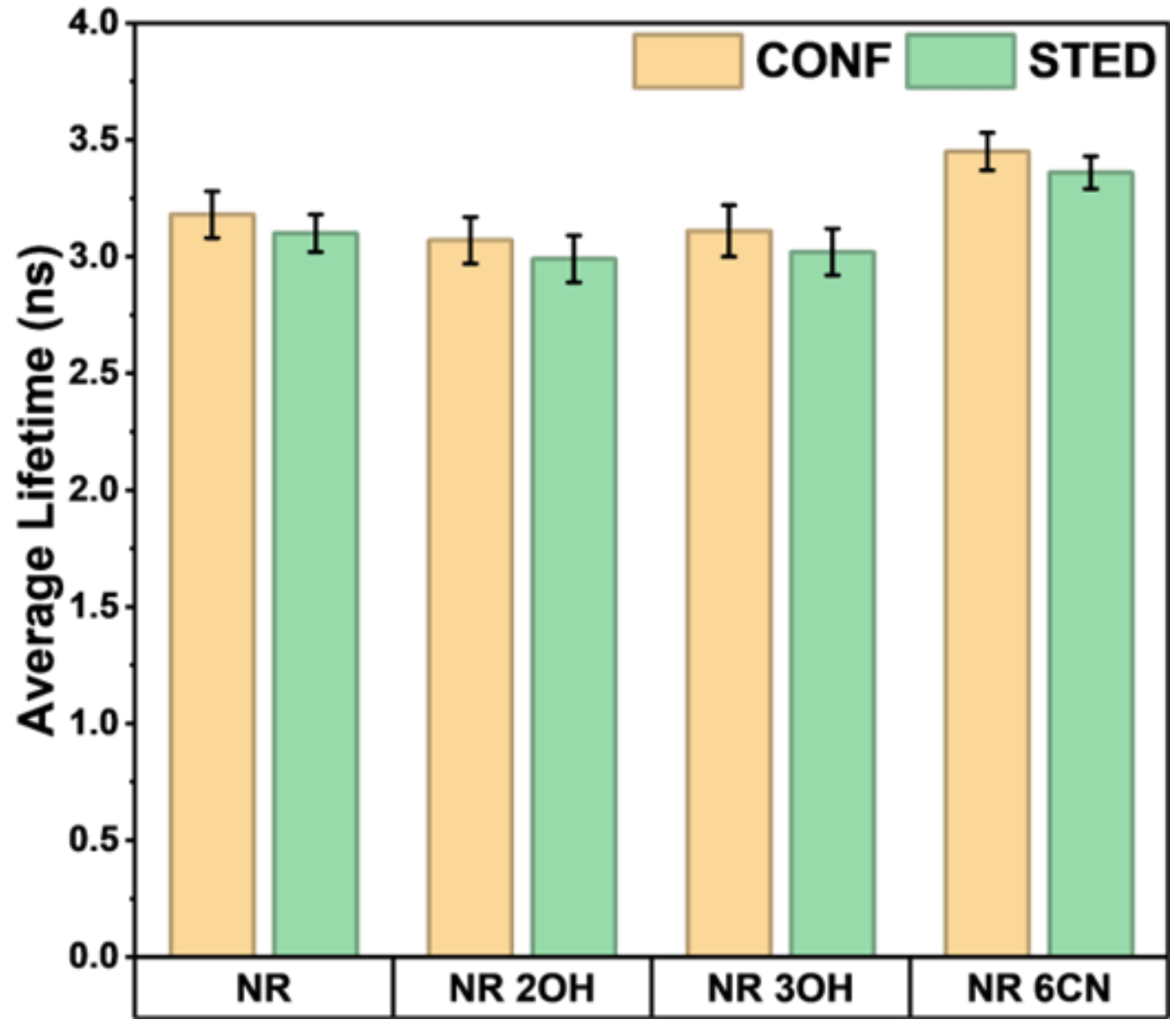

Fig. S16. Cell-averaged lifetime statistics measured in confocal and STED imaging modes. Data are mean ± s.d. (n ≥ 20 cells per analog).

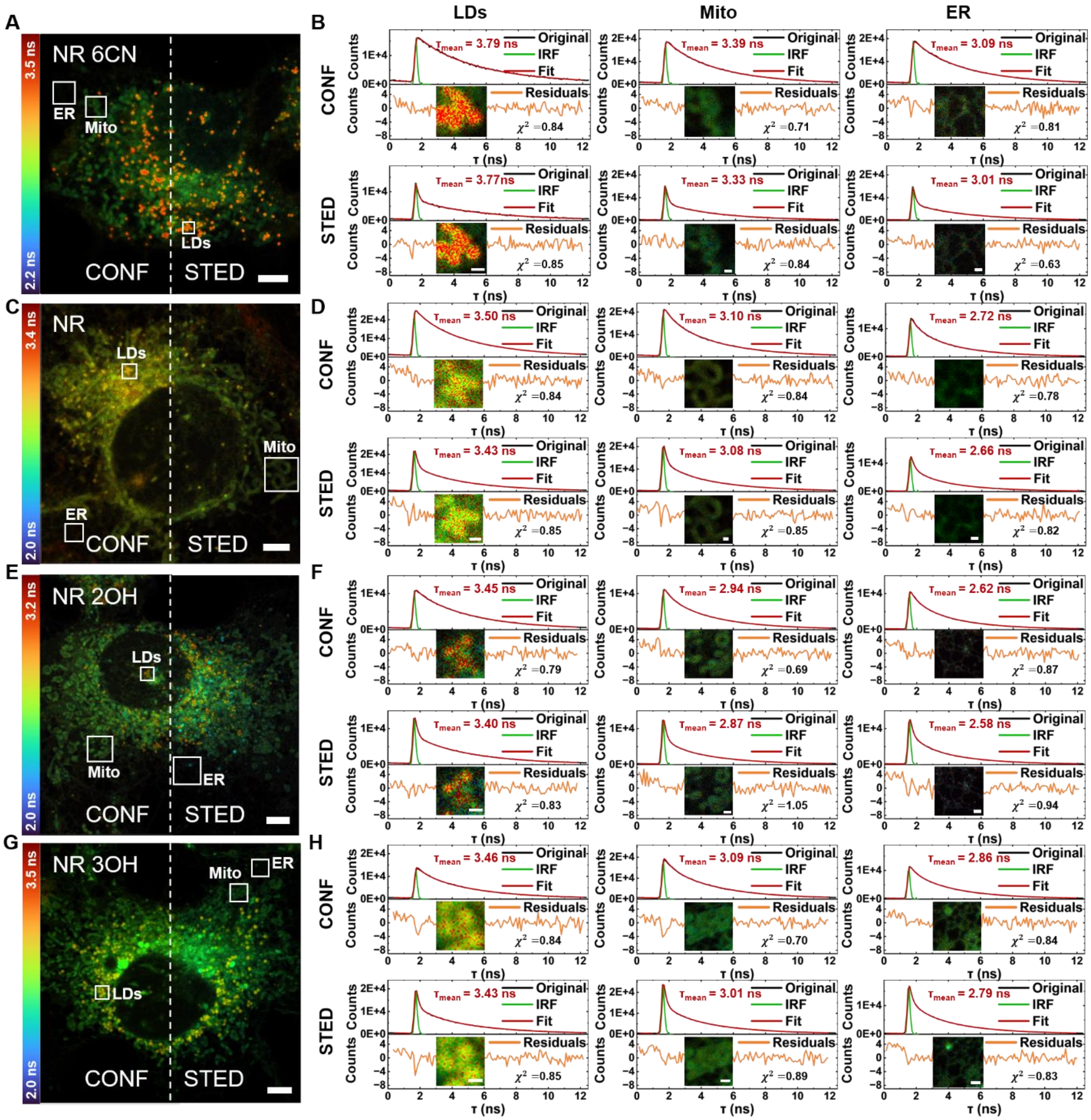

Fig. S17. Organelle-resolved lifetime analysis of NR analogs in confocal and STED imaging. (A) Representative CONF-FLIM and STED-FLIM images of COS-7 cells stained with NR 6CN from the same field of view. (B) Representative organelle selection in (A) and its corresponding fluorescence lifetime curve. The first and second rows are the CONF and STED modes, respectively, with lipid droplets (LDs), mitochondria (mito), and ER arranged from left to right. (C) Representative CONF-FLIM and STED-FLIM images of COS-7 cells stained with the NR from the same field of view. (D) Representative organelle selection in (C) and its corresponding fluorescence lifetime curve. (E) Representative CONF-FLIM and STED-FLIM images of COS-7 cells stained with the NR 2OH from the same field of view. (F) Representative organelle selection in (E) and its corresponding fluorescence lifetime curve. (G) Representative CONF-FLIM and

STED-FLIM images of COS-7 cells stained with the NR 3OH from the same field of view. (H) Representative organelle selection in (G) and its corresponding fluorescence lifetime curve. All lifetime curves were fitted in LAS X FLIM software using tri-exponential reconvolution. Scale bars, 5 µm.

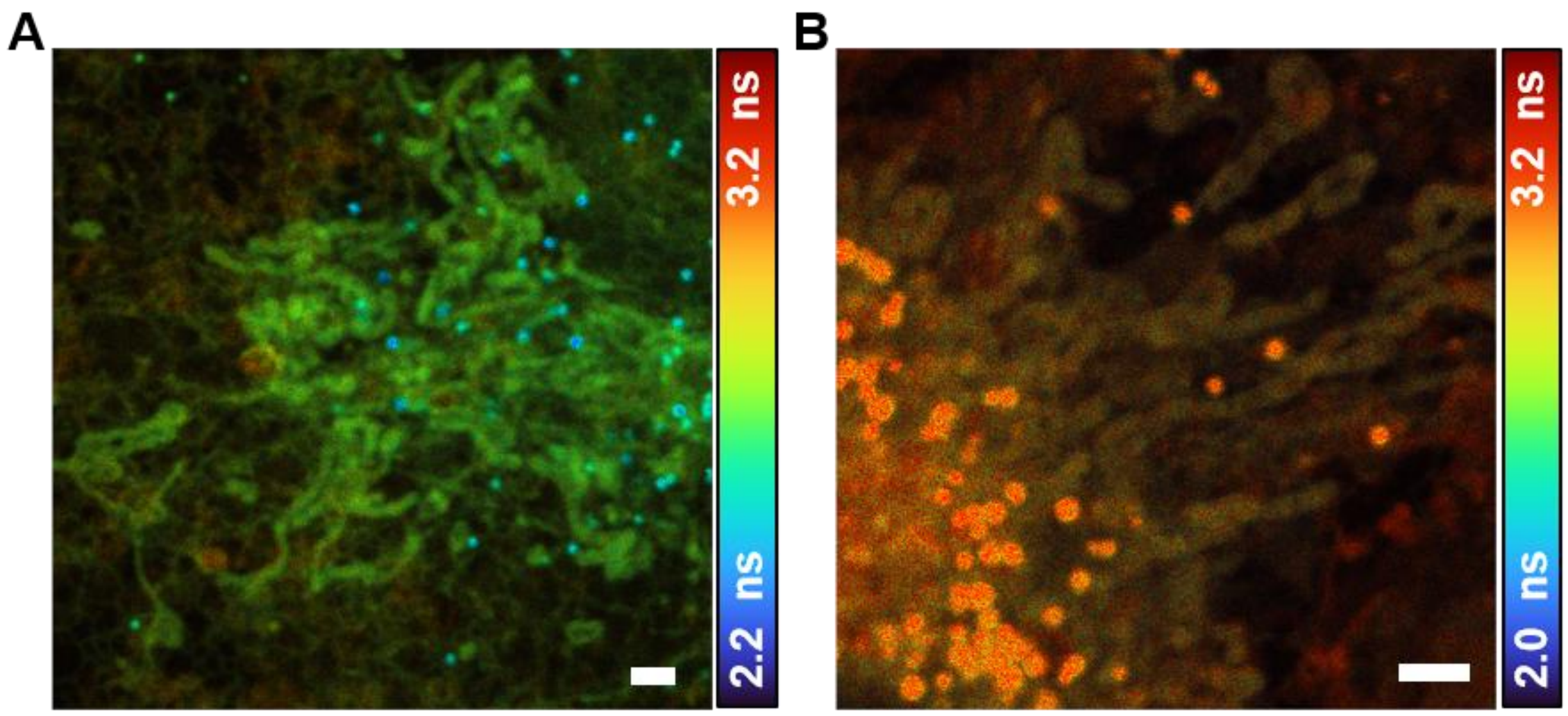

Fig. S18. Low-lifetime aggregates detected in NR-stained COS-7 cells. (A) Aggregates with fluorescence lifetimes lower than those of mitochondria. (B) Under basal conditions, the fluorescence lifetime in lipid droplets is higher than that in mitochondria. Scale bars, 2 µm.

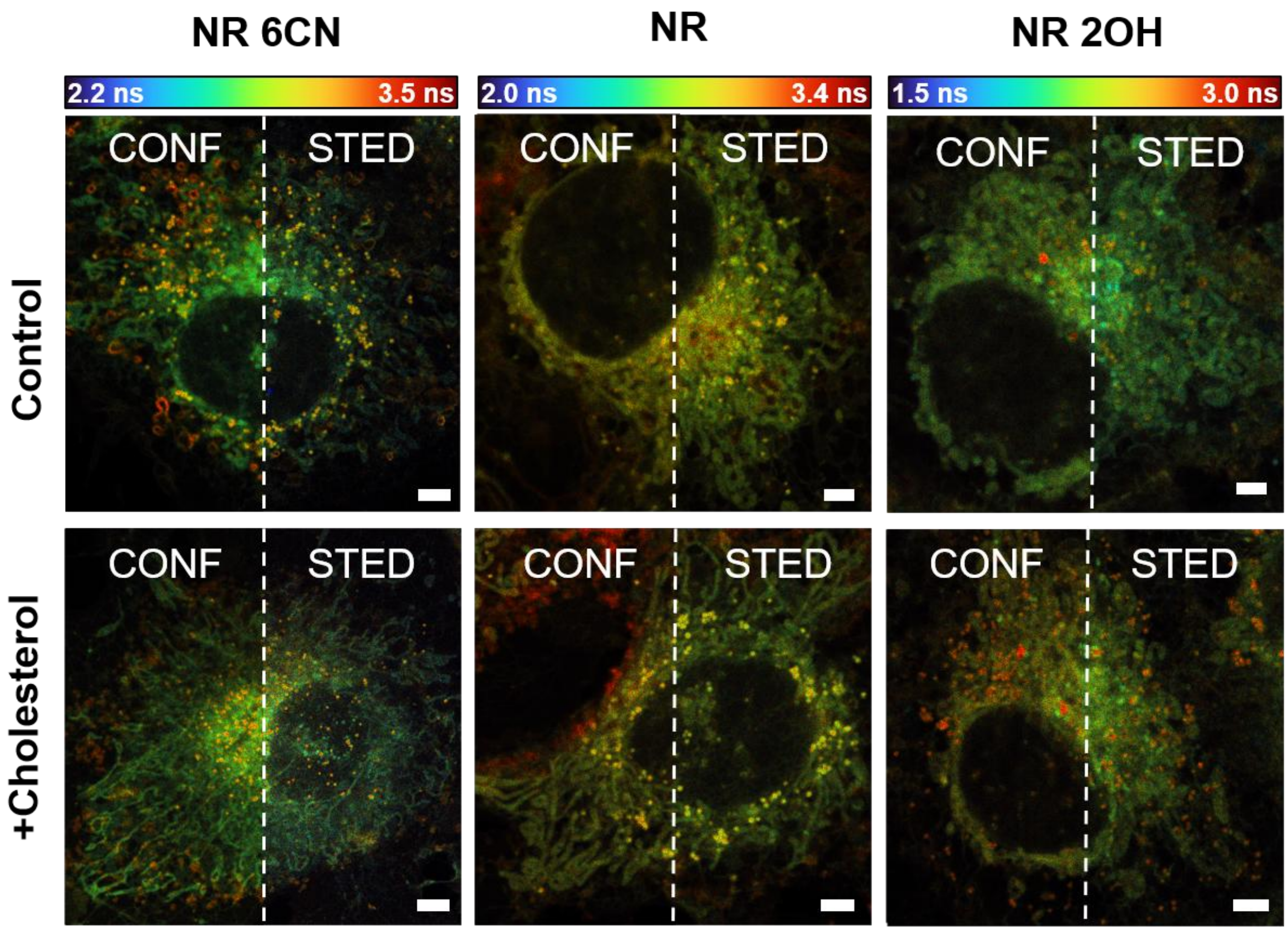

Fig. S19. Confocal-FLIM and STED-FLIM imaging of NR analogs after cholesterol treatment. Representative CONF-FLIM and STED-FLIM images of COS-7 cells stained with NR, NR 2OH, and NR 6CN after cholesterol treatment (1 mM, 20 min). Scale bars, 5 µm.

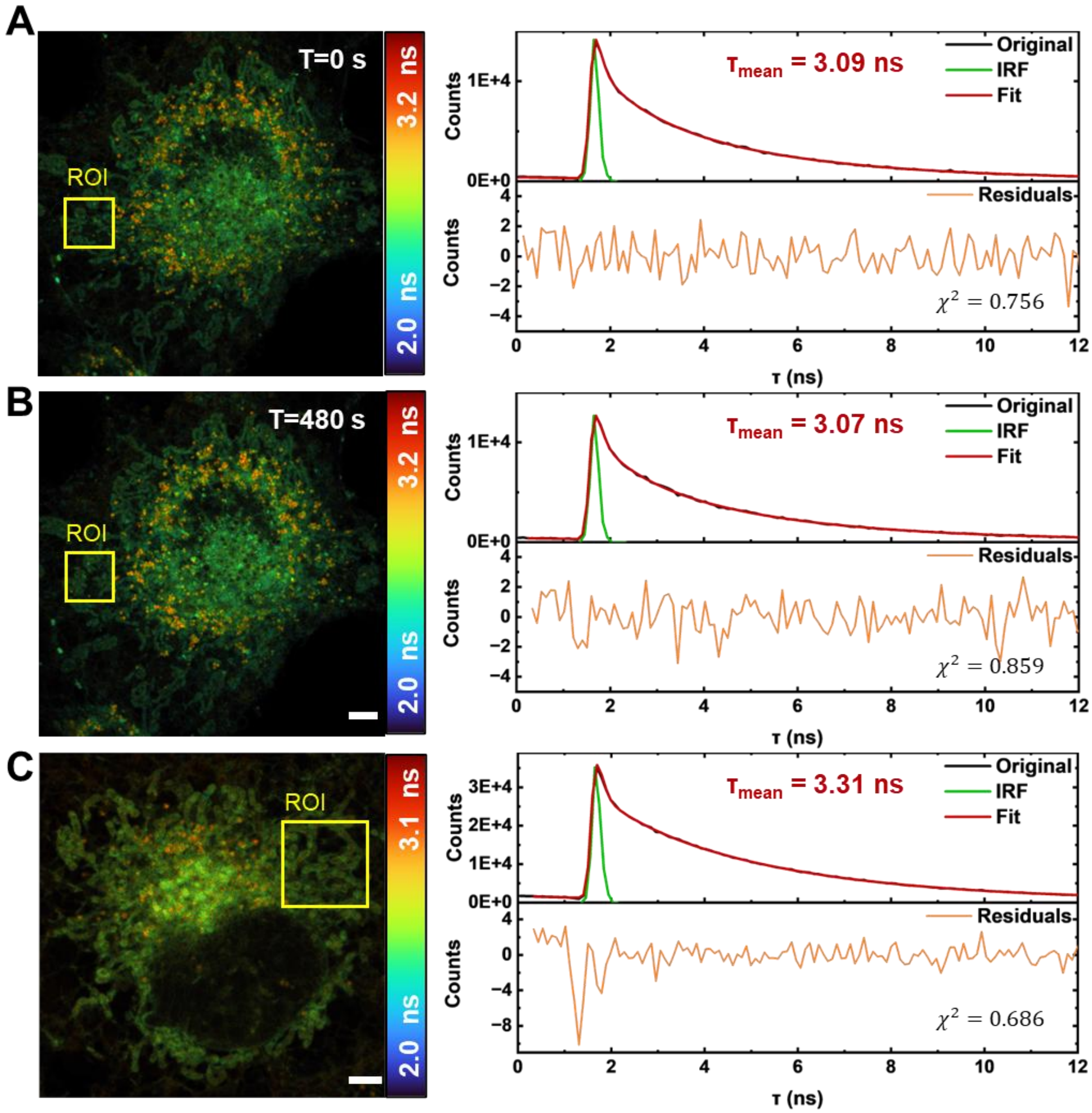

Fig. S20. Representative lifetime fitting during time-lapse STED-FLIM imaging and after cholesterol treatment. Fluorescence lifetime fitting by tri-exponential reconvolution ($\chi^2 < 1$) of the same region of interest (ROI) at (A) 0 s and (B) 480 s during time-lapse STED-FLIM imaging (20-s interval) of COS-7 cells stained with NR 3OH. The mean lifetimes are 3.09 and 3.07 ns, respectively. (C) Tri-exponential reconvolution fitting ($\chi^2 < 1$) of an ROI in STED-FLIM images of COS-7 cells stained with NR 3OH after cholesterol treatment. The mean lifetime is 3.31 ns. Scale bars, 5 μm.

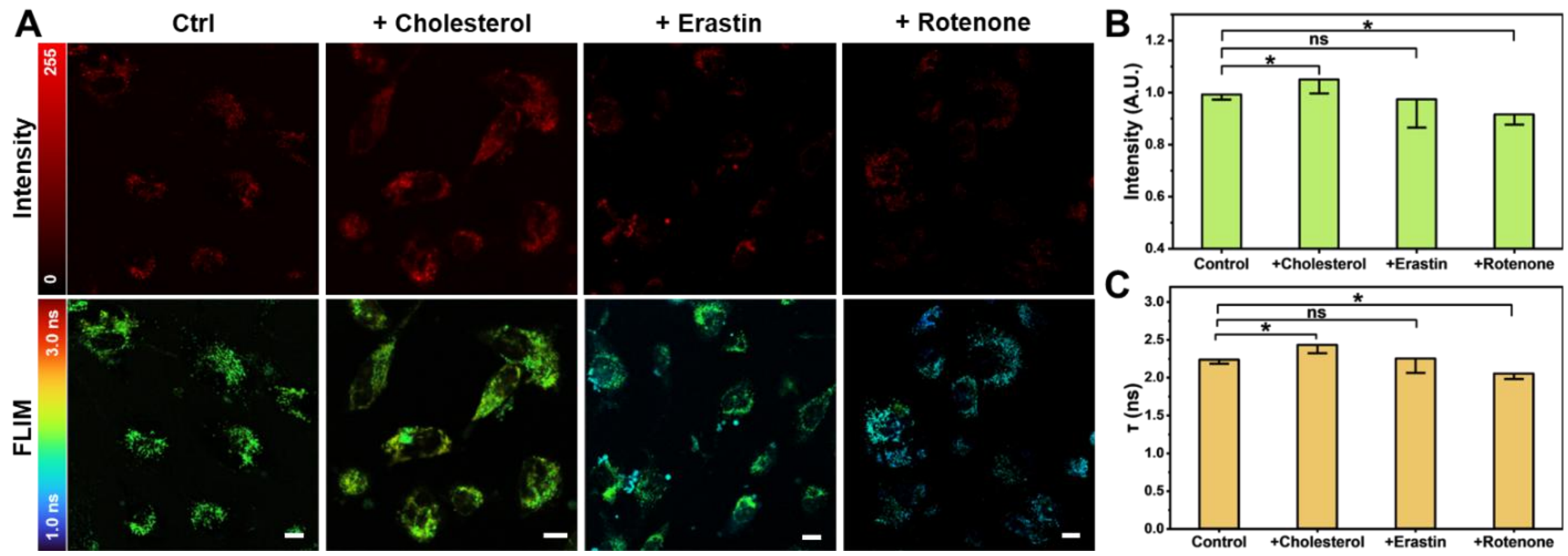

Fig. S21. Validation of intracellular viscosity changes using the fluorescent molecular rotor CCVJ. (A) Fluorescence and FLIM images of untreated, cholesterol-treated (1 mM, 30 min), erastin-treated (5 μM, 3 h), and rotenone-treated (1 μM, 20 min) COS-7 cells stained with CCVJ. Both CCVJ fluorescence intensity and lifetime increase with microviscosity. Scale bars, 10 μm. (B) Mean cellular fluorescence intensities normalized to untreated control, indicating relative changes in intracellular viscosity. Data are mean ± s.d. (n ≥ 20 cells per condition). (C) Cell-averaged lifetime statistics. Data are mean ± s.d. (n ≥ 20 cells per condition). Statistical significance was determined by t test (ns, not significant; *P < 0.05).

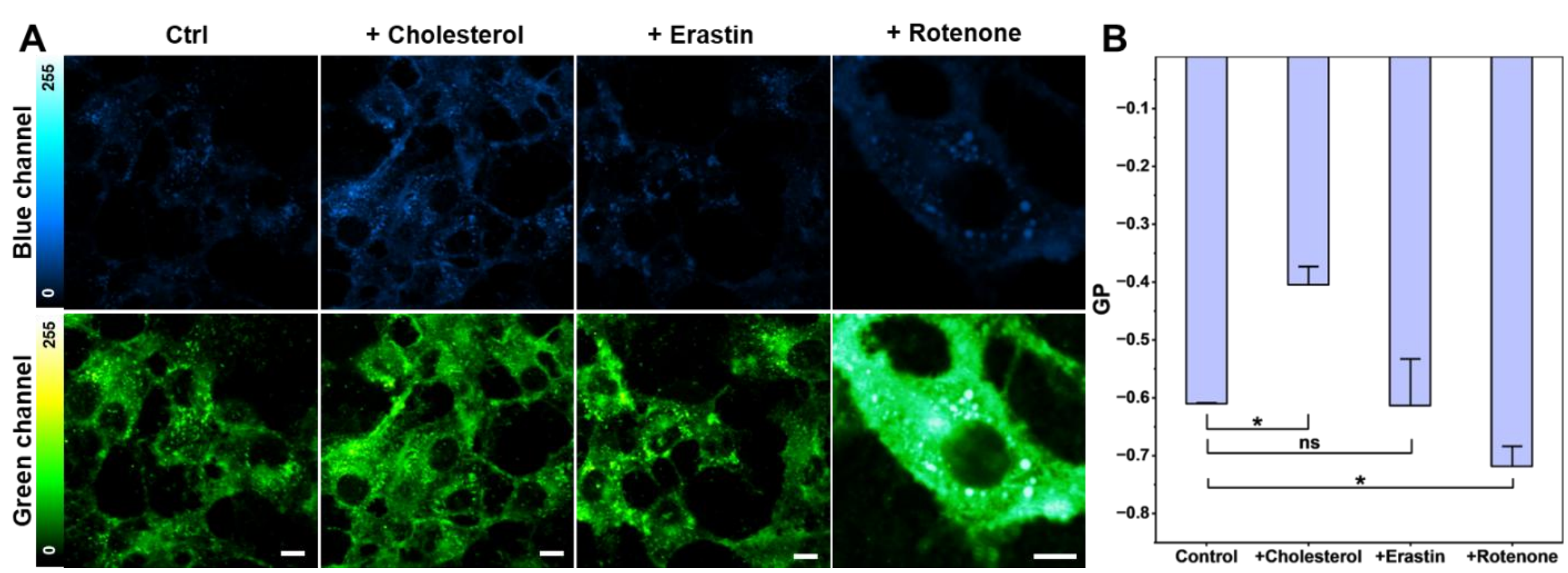

Fig. S22. Validation of membrane order changes using Laurdan. (A) Fluorescence images of untreated, cholesterol-treated (1 mM, 30 min), erastin-treated (5 μM, 3 h), and rotenone-treated (1 μM, 20 min) COS-7 cells stained with Laurdan. Generalized polarization (GP) was calculated as $GP = (I_{blue} - I_{green})/(I_{blue} + I_{green})$, where higher GP values indicate increased membrane order and reduced membrane hydration. Scale bars, 10 μm. (B) GP statistics under the indicated treatments. Data are mean ± s.d. (n ≥ 20 cells per condition). Statistical significance was determined by t test (ns, not significant; *P < 0.05).

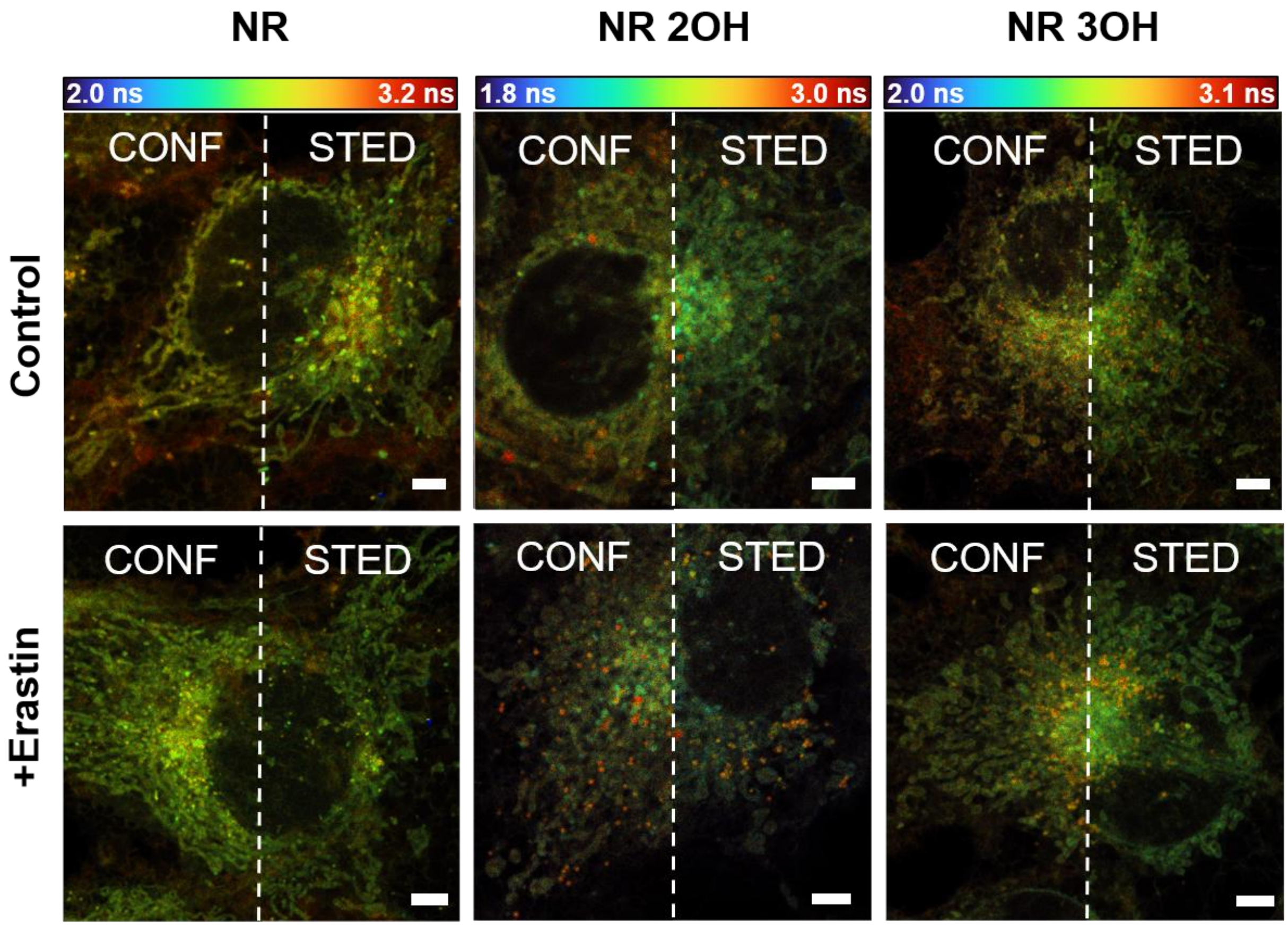

Fig. S23. Confocal-FLIM and STED-FLIM imaging of NR analogs after erastin treatment. Representative CONF-FLIM and STED-FLIM images of COS-7 cells stained with NR, NR 2OH, and NR 3OH after erastin treatment (5 µM, 3 h). Scale bars, 5 µm.

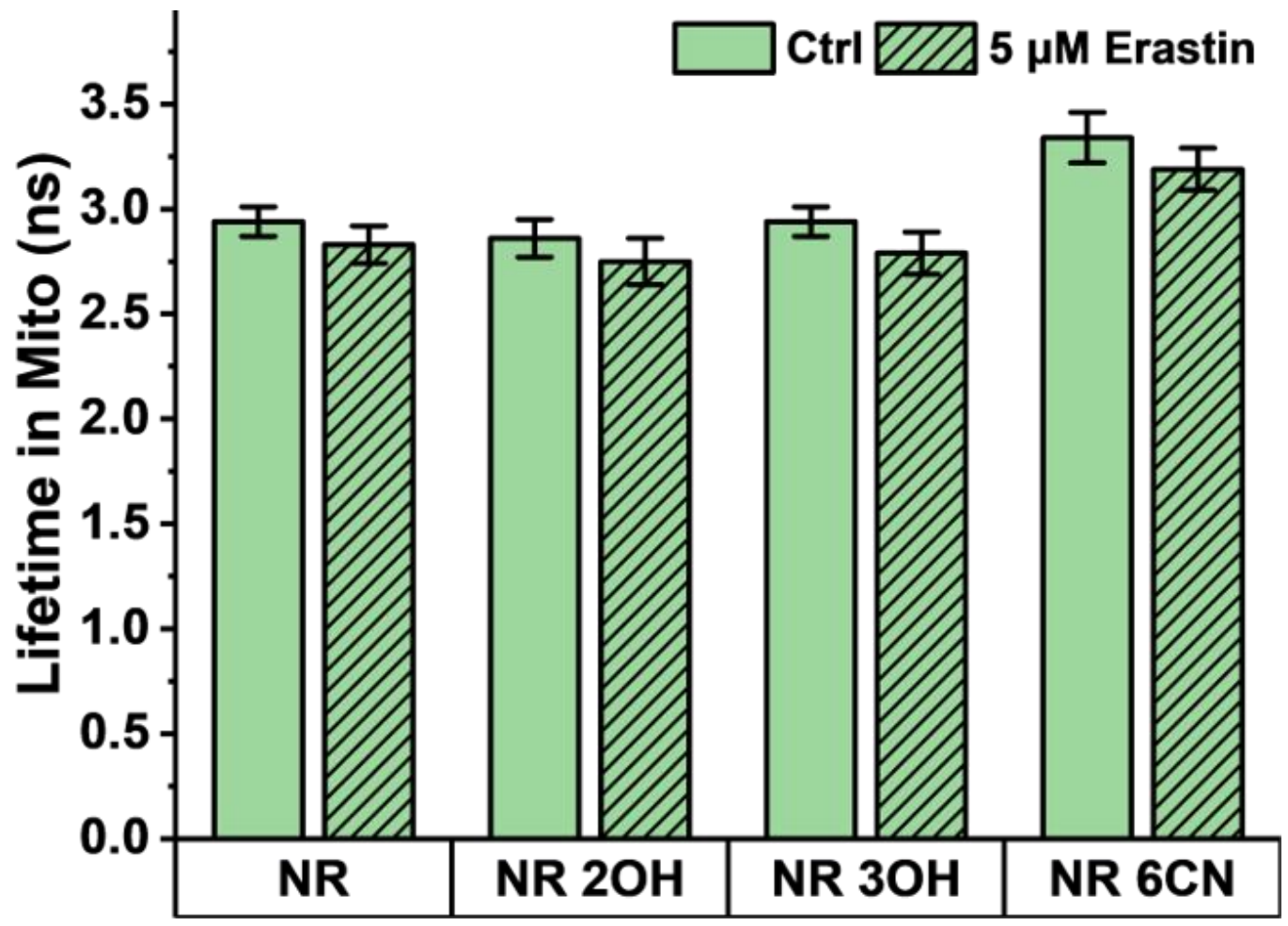

Fig. S24. Mitochondrial lifetime changes after erastin treatment across NR analogs. Data are mean ± s.d. ($n \geq 25$ measurements per analog).

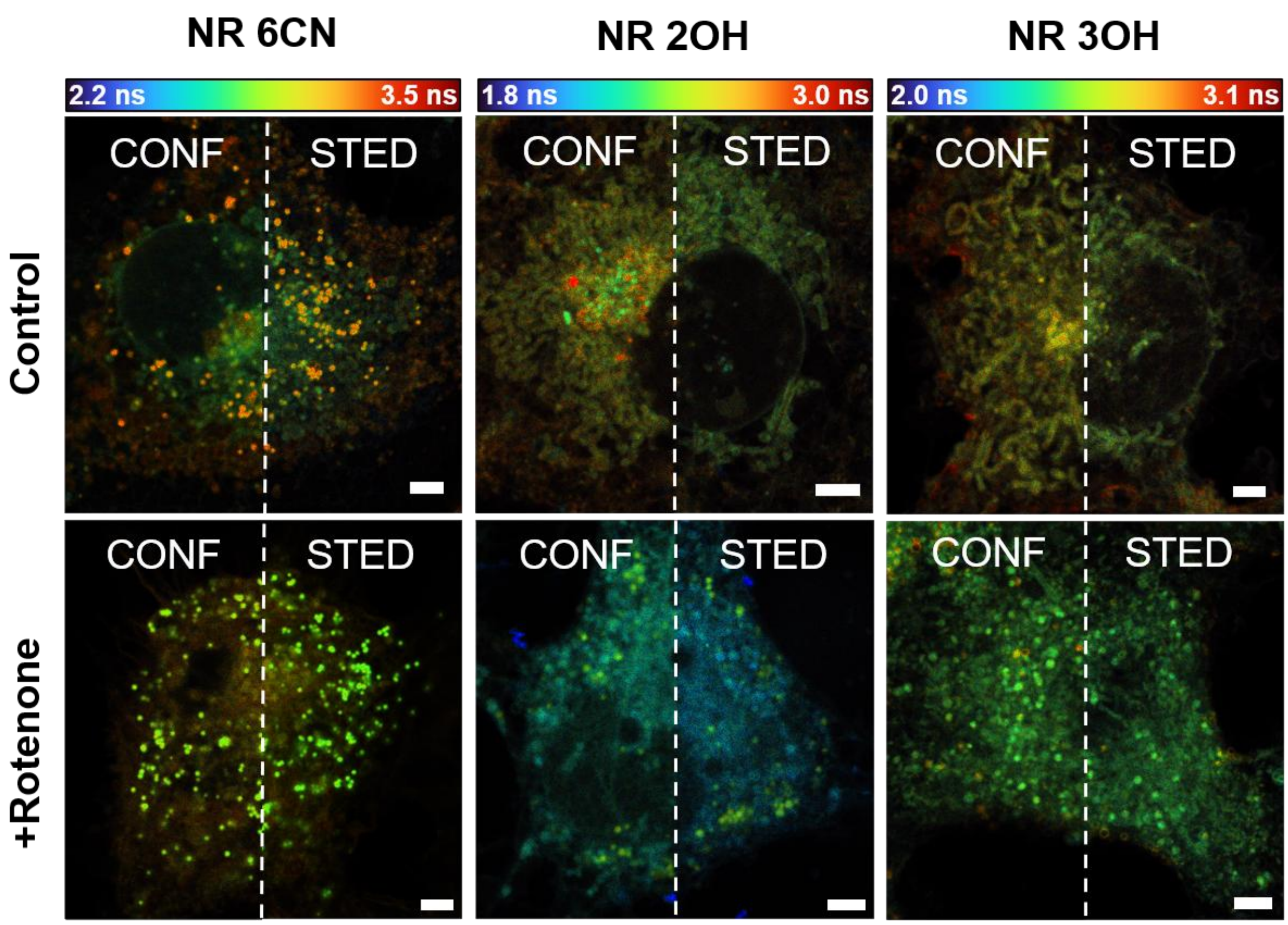

Fig. S25. Confocal-FLIM and STED-FLIM imaging of NR after rotenone treatment. Representative CONF-FLIM and STED-FLIM images of COS-7 cells stained with NR after rotenone treatment (1 μM, 20 min). Scale bars, 5 μm.

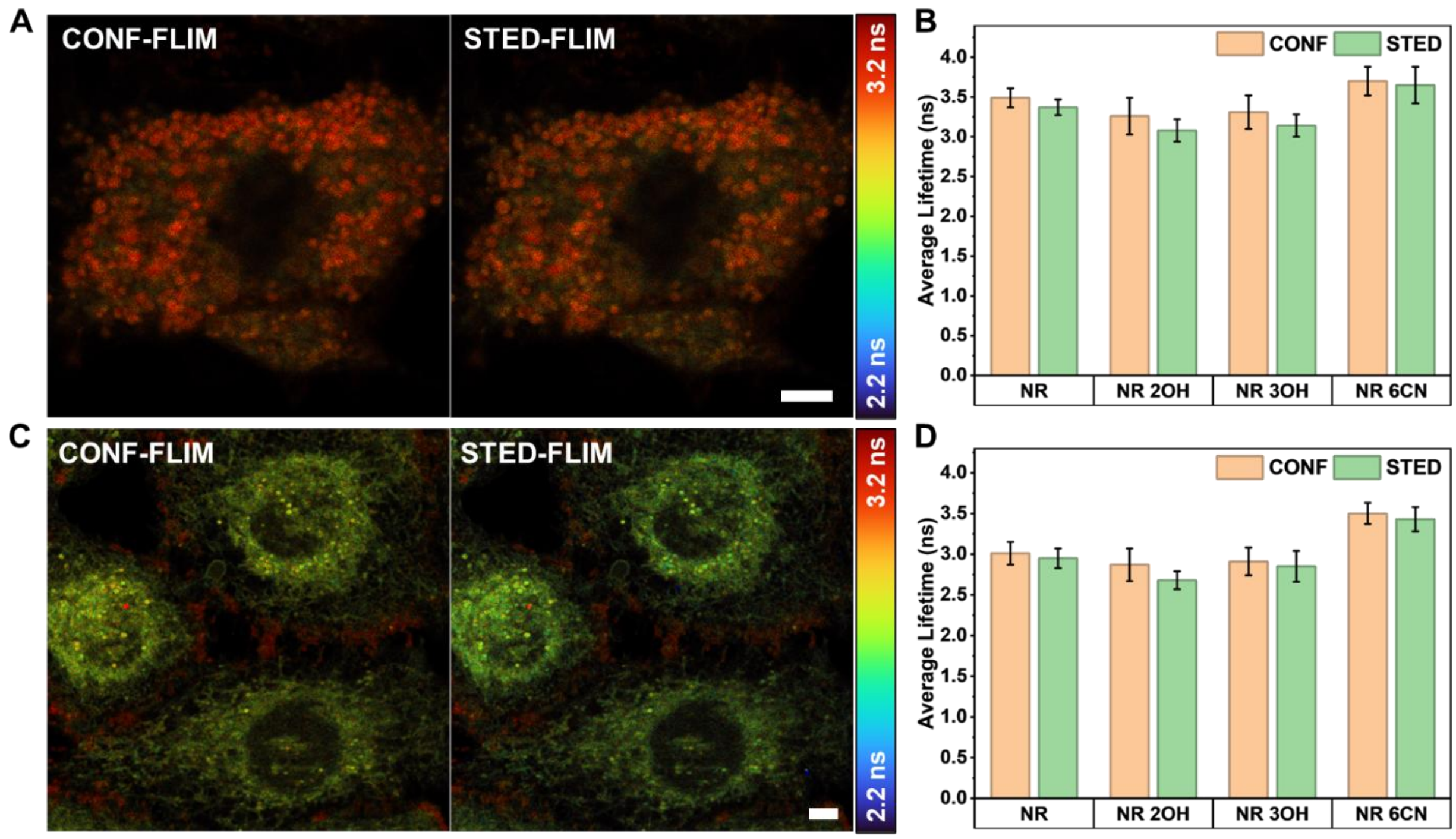

Fig. S26. CONF-FLIM and STED-FLIM imaging in additional cell lines. (A) Representative CONF-FLIM and STED-FLIM images of GL261 cells stained with NR 3OH. Scale bar, 5 µm. (B) Cell-averaged lifetime statistics measured in confocal and STED imaging modes for GL261 cells stained with NR 3OH. Data are mean ± s.d. (n ≥ 20 cells). (C) Representative CONF-FLIM and STED-FLIM images of HeLa cells stained with NR. Scale bar, 5 µm. (D) Cell-averaged lifetime statistics measured in confocal and STED imaging modes for HeLa cells stained with NR. Data are mean ± s.d. (n ≥ 20 cells).

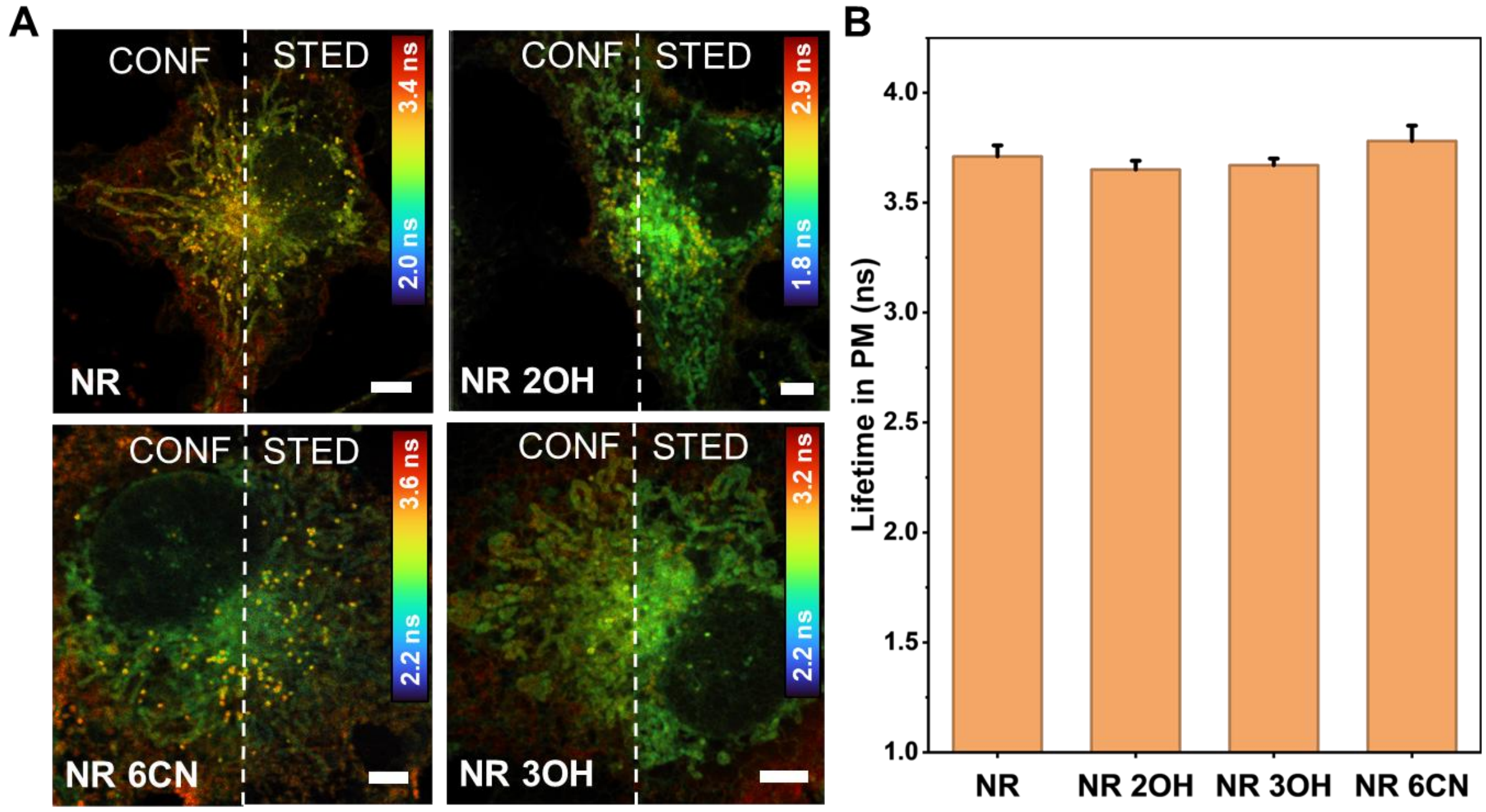

Fig. S27. Fluorescence lifetime of NR analogs at the plasma membrane. (A) Representative CONF-FLIM and STED-FLIM images of the plasma membrane in COS-7 cells stained with the NR analogs. Scale bars, 5 µm. (B) Fluorescence lifetime statistics of the probes at the plasma membrane. Data are mean ± s.d. (n ≥ 25 measurements per analog).

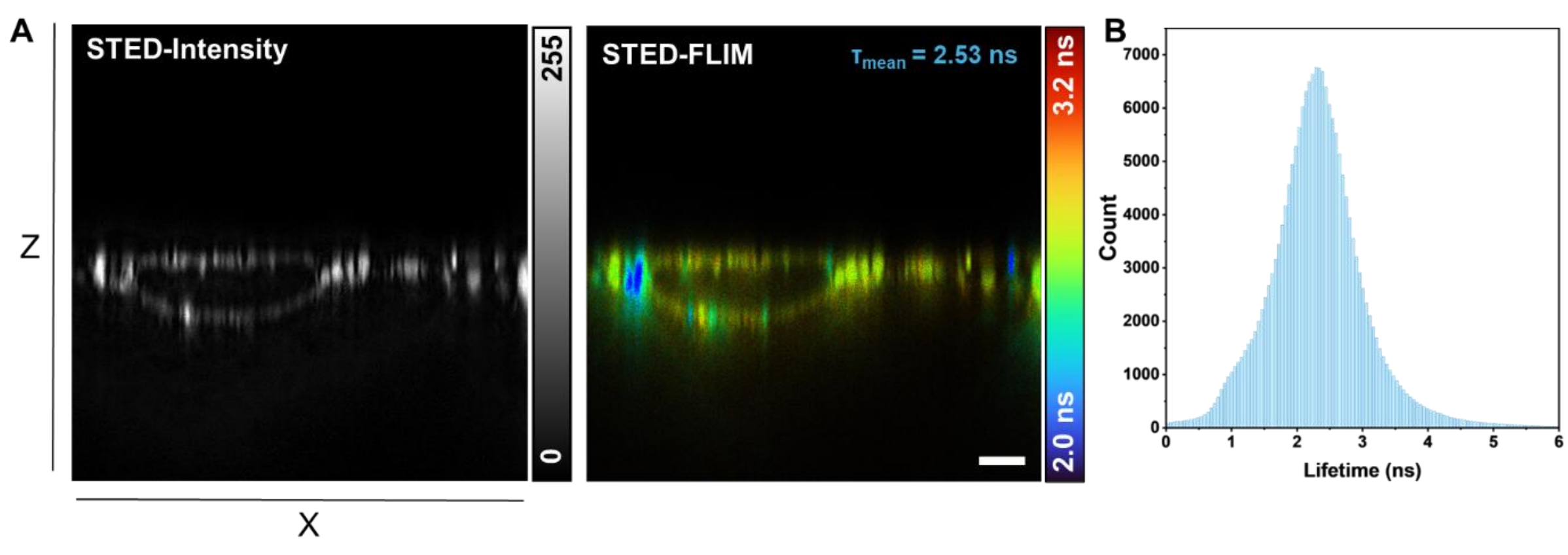

Fig. S28. Z-stack STED intensity and STED-FLIM imaging of living COS-7 cells stained with NR. (A) Z-axis STED intensity and STED-FLIM imaging of living COS-7 cells stained with NR. Scale bar, 5 µm. (B) Fluorescence lifetime histogram corresponding to (A).

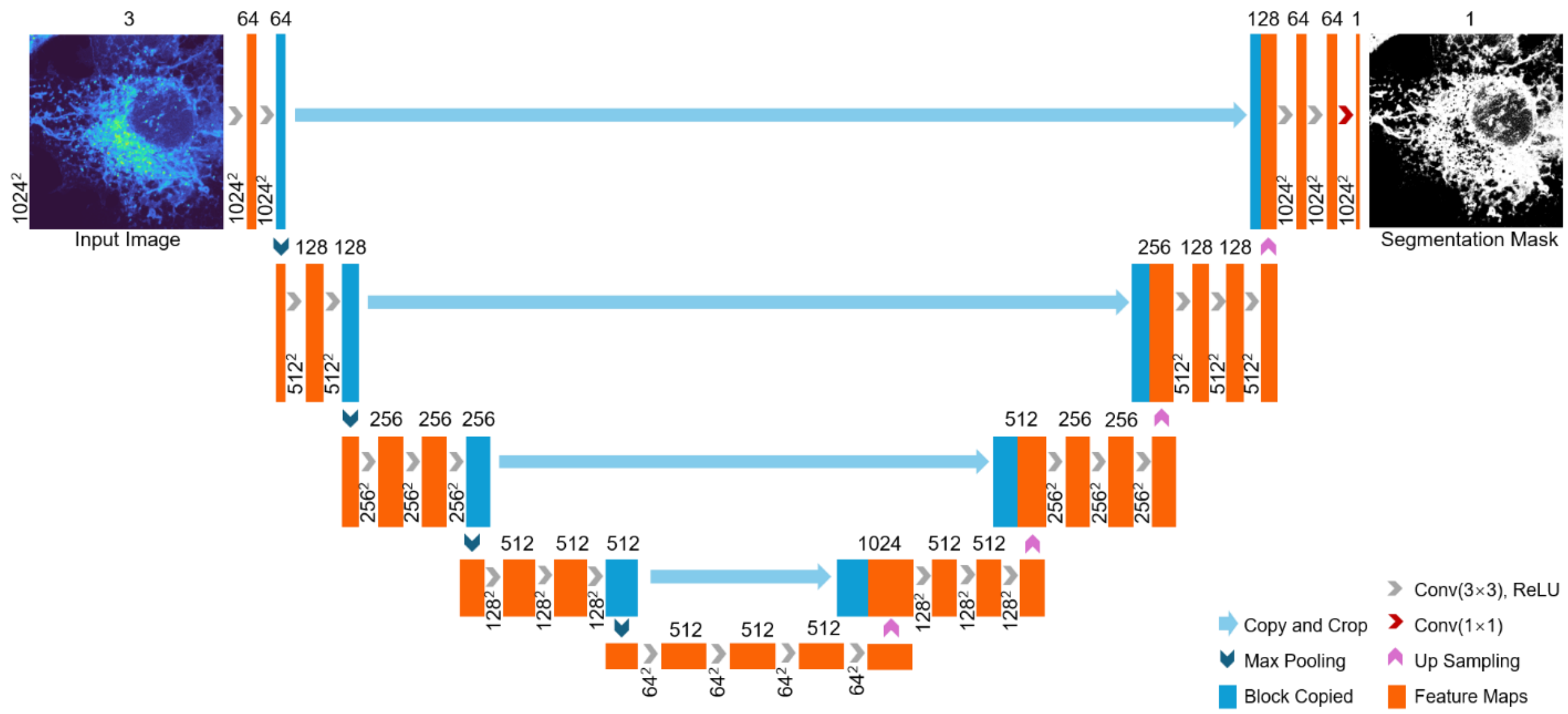

Fig. S29. Architecture of the VGG16-UNet image segmentation network. This figure provides a supplementary explanation of **Fig. 4A** in the main text and shows the mitochondrial output as a representative segmentation result.

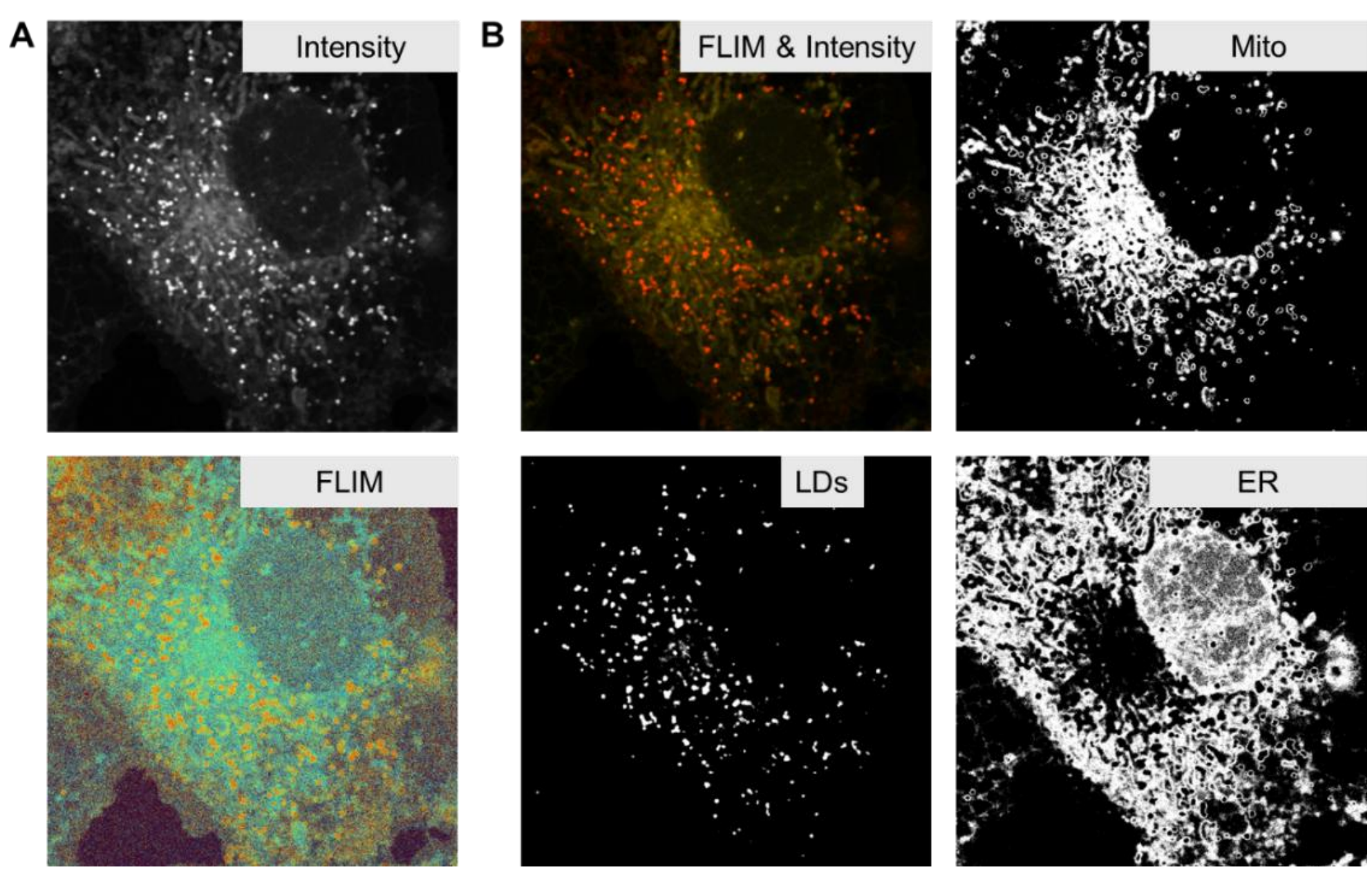

Fig. S30. Training data used for the VGG16-UNet model. (A) Raw STED-FLIM images represented by intensity and fluorescence lifetime, respectively. (B) Intensity-weighted fluorescence lifetime image and the corresponding manually segmented reference mask.

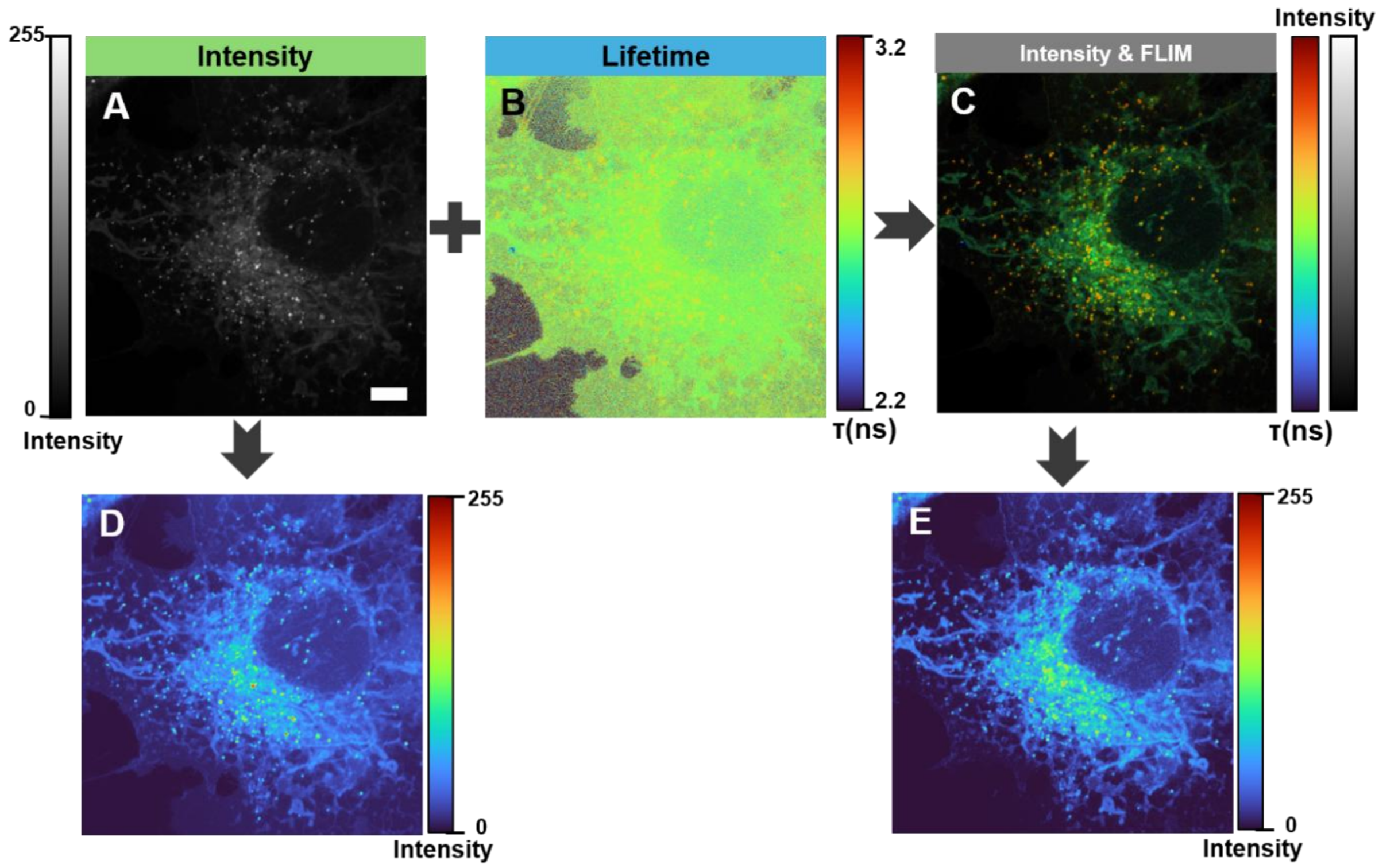

Fig. S31. Preprocessing of STED-FLIM images. This figure provides a detailed supplement to the image shown in **Fig. 4A** of the main text. (A) Raw STED image. (B) Raw STED-FLIM image. (C) Intensity-weighted STED-FLIM image generated by LAS X software. (D) Pseudo-colored STED image. (E) Pseudo-colored STED-FLIM image. Scale bar, 5 µm.

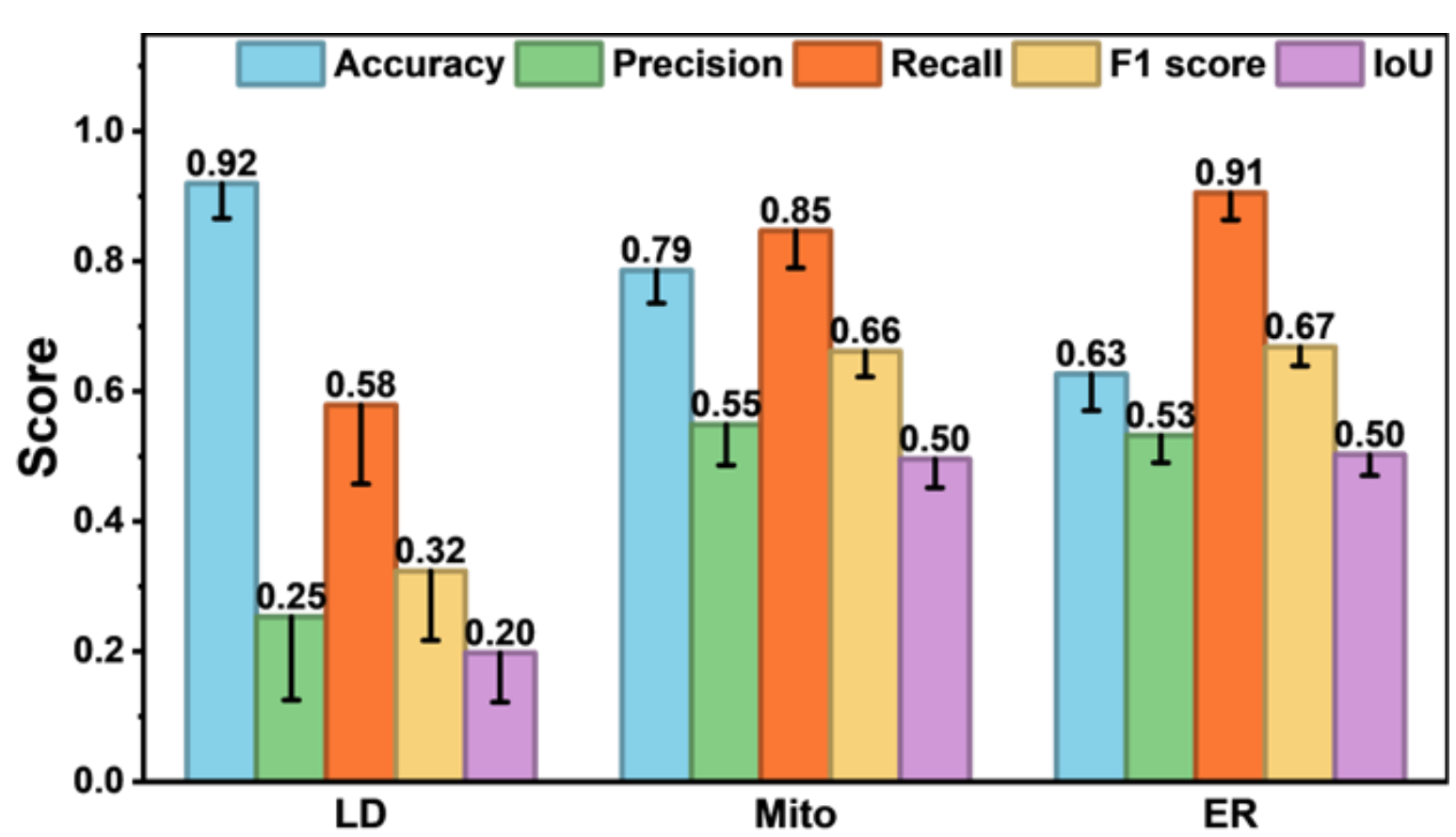

Fig. S32. Training performance of the model using pseudo-colored intensity-only STED images as input. Data are mean ± s.d. from five-fold cross-validation.

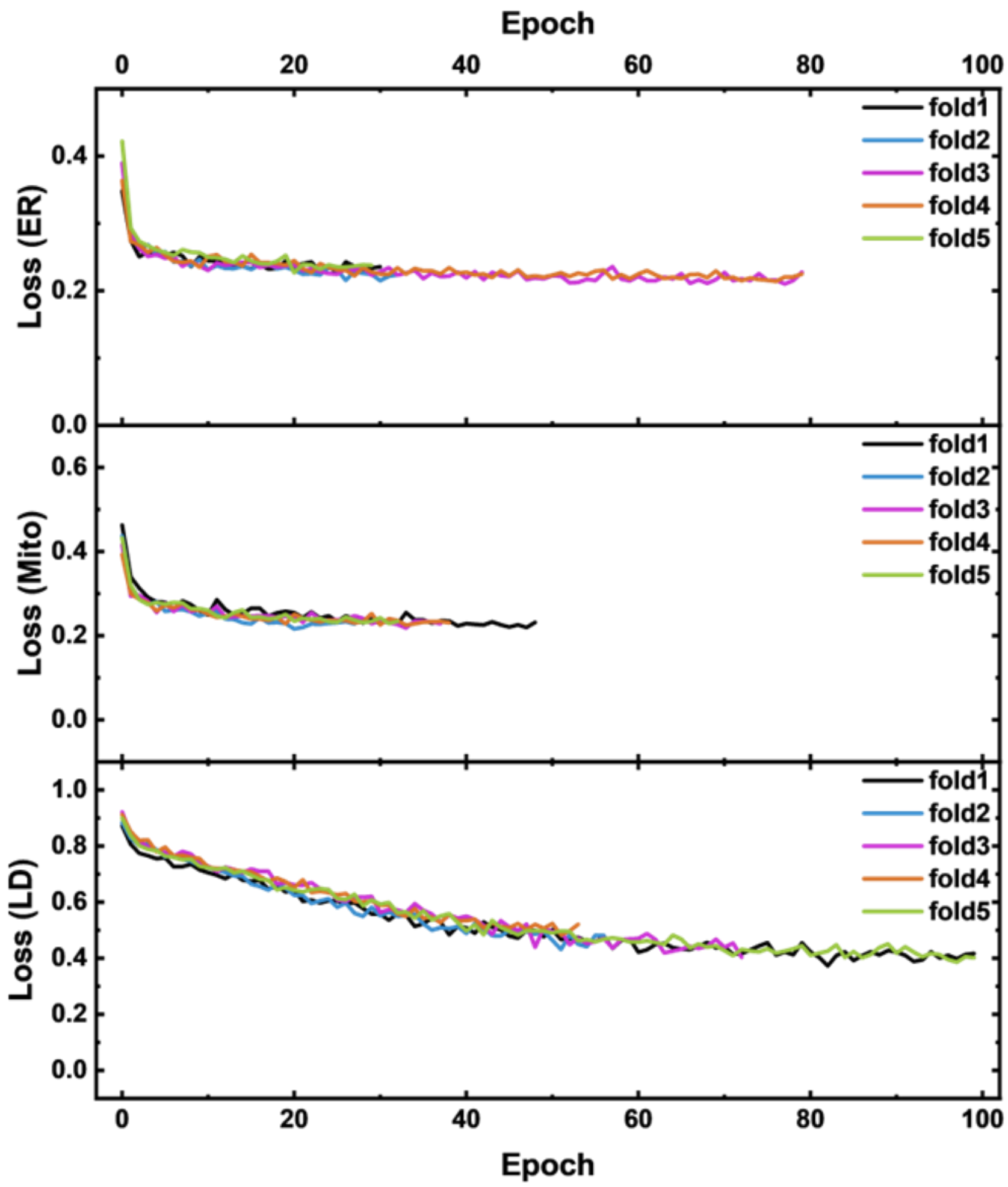

Fig. S33. Cross-validation loss curves for organelle segmentation models. Loss curves of the LD, mitochondria, and ER segmentation models across the five cross-validation folds.

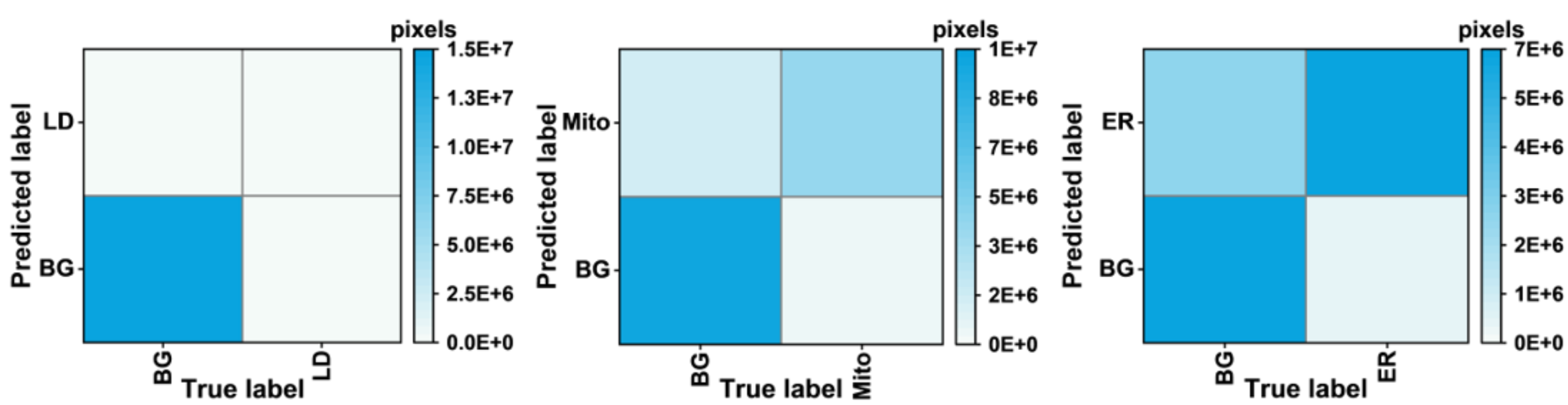

Fig. S34. Representative confusion matrices for LD, mitochondria, and ER segmentation models. Shown are representative confusion matrices obtained with pseudo-colored inputs.

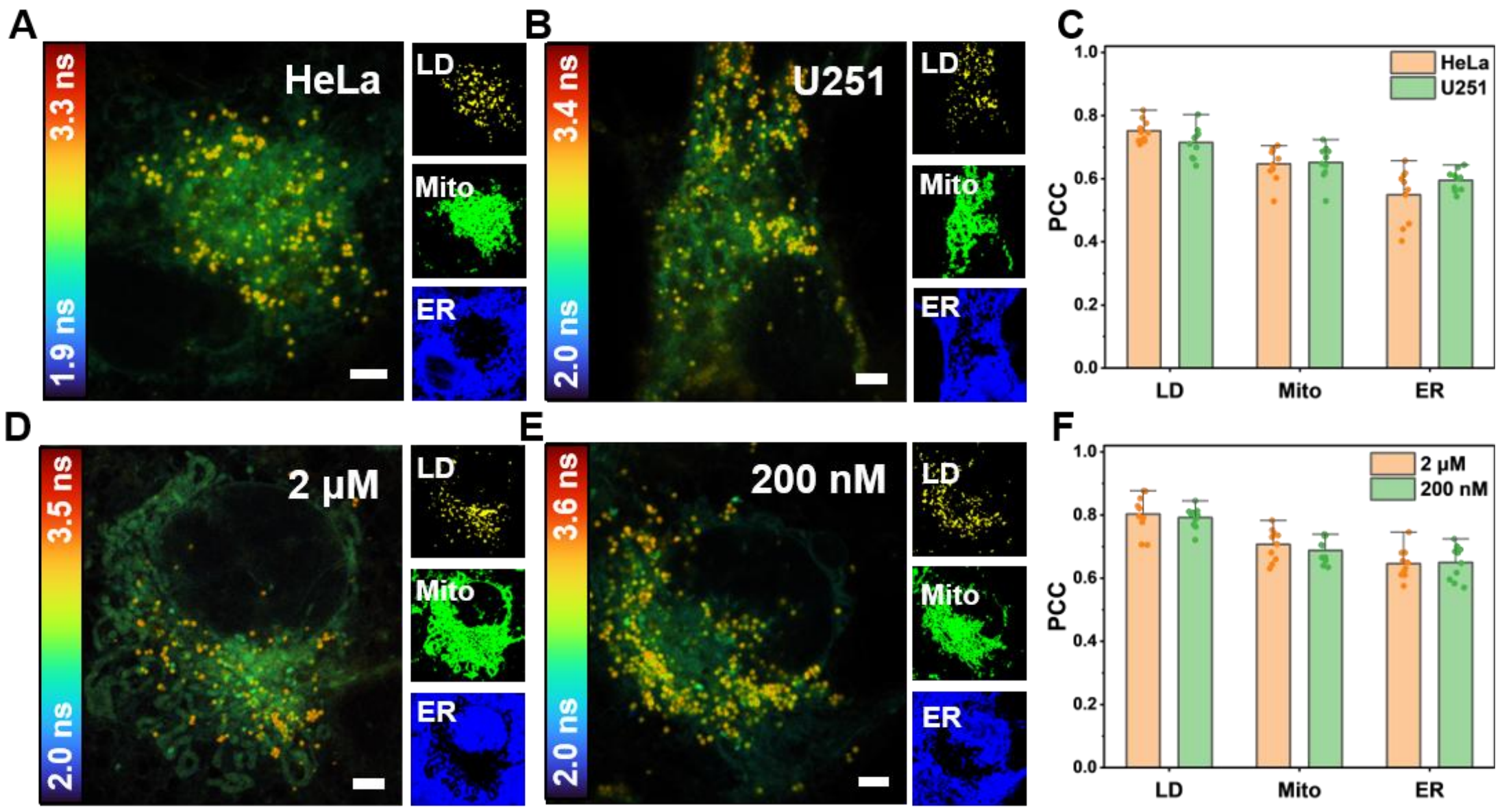

Fig. S35. Model performance on independent datasets with different cell lines and probe concentrations. (A) Representative STED-FLIM image of HeLa cells stained with NR 6CN (500 nM) and the corresponding predicted organelle masks. (B) Representative STED-FLIM image of U251 cells stained with NR 6CN (500 nM) and the corresponding predicted organelle masks. (C) The PCC of different organelles between DL model-predicted organelle maps and corresponding commercial organelle-marker images. Data are mean ± s.d. (n = 10 samples per model). (D) Representative STED-FLIM image of COS-7 cells stained with high concentration (2 μM) NR 6CN and the corresponding predicted organelle masks. (E) Representative STED-FLIM image of COS-7 cells stained with low concentration (200 nM) NR 6CN and the corresponding predicted organelle masks. (F) The PCC of different organelles between DL model-predicted organelle maps and corresponding commercial organelle-marker images. Data are mean ± s.d. (n = 10 samples per model). Scale bars, 5 μm.

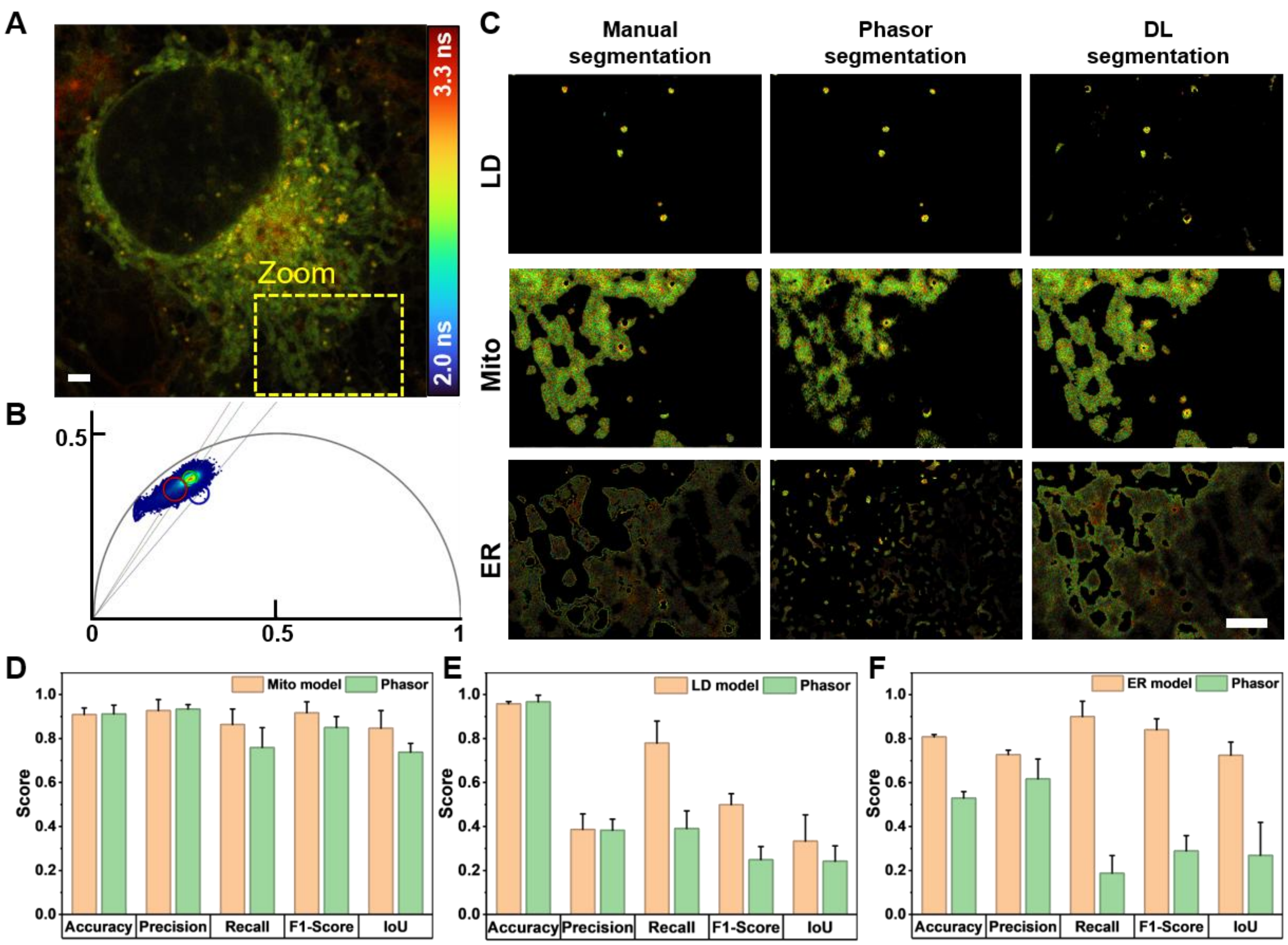

Fig. S36. Comparison between deep-learning-based and phasor-based segmentation. (A and B) Representative STED-FLIM images of NR-stained COS-7 cells and the corresponding phasor plots. (C) Comparison of representative organelle segmentation results using manual segmentation, phasor segmentation, and deep learning (DL) segmentation. (D) Performance comparison between the Mito model and phasor-based segmentation using manually annotated binary masks as ground truth, evaluated by accuracy, precision, recall, F1 score, and intersection-over-union (IoU). (E) Performance comparison between the LD model and phasor-based segmentation. (F) Performance comparison between the ER model and phasor-based segmentation. Data are mean ± s.d. ($n \geq 10$ samples per model). Scale bars, 2 μm.

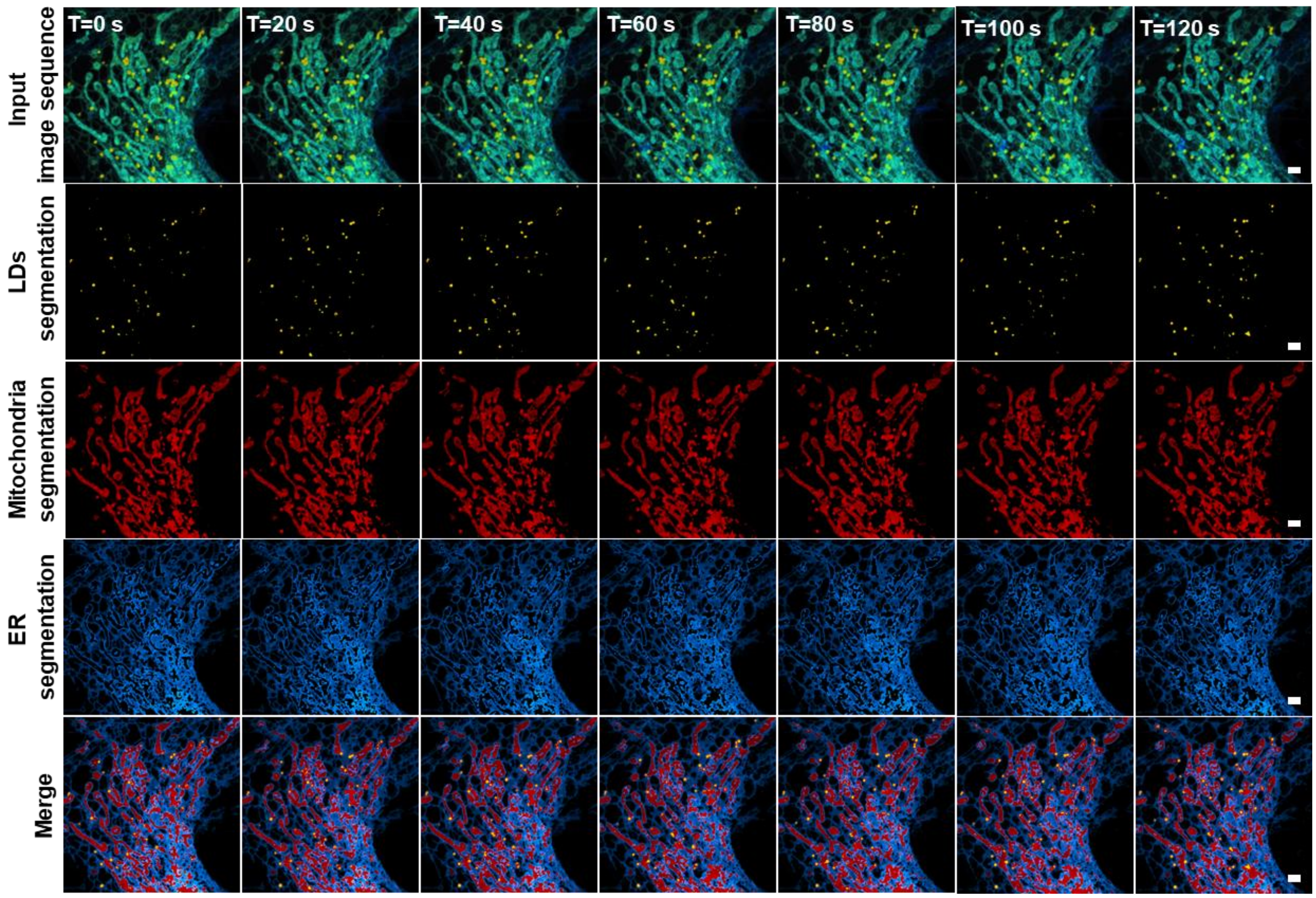

Fig. S37. Time-lapse segmentation of three organelles during ferroptosis. LDs, mitochondria, and ER were rapidly segmented by the VGG16-UNet model from time-lapse images of cells stained with NR 3OH during ferroptosis. Scale bars, 2 μm.

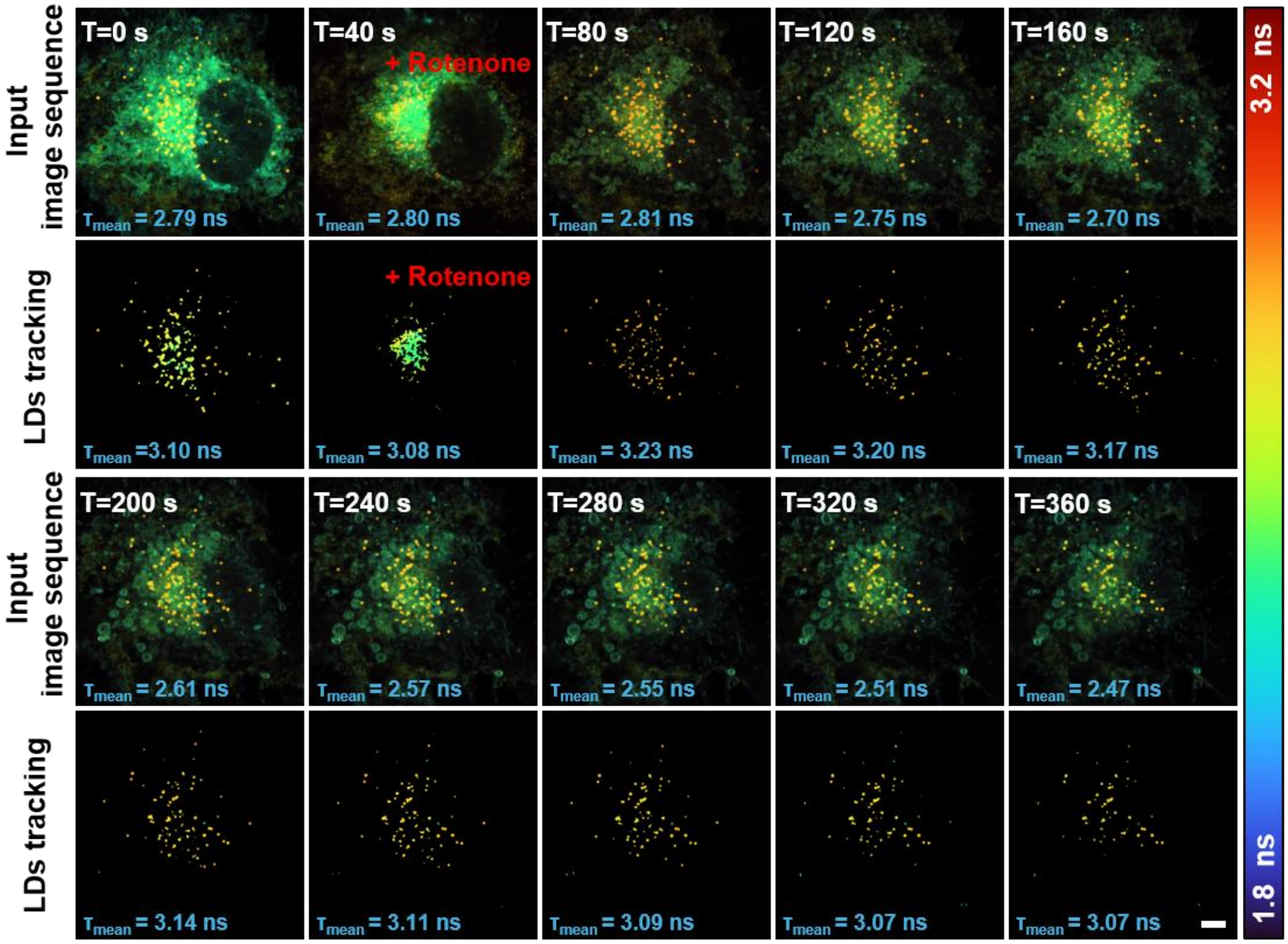

Fig. S38. Time-lapse tracking of lipid droplets during rotenone treatment. The VGG16-UNet model was used to track LDs in COS-7 cells during rotenone treatment (~5 μM). Scale bar, 5 μm.

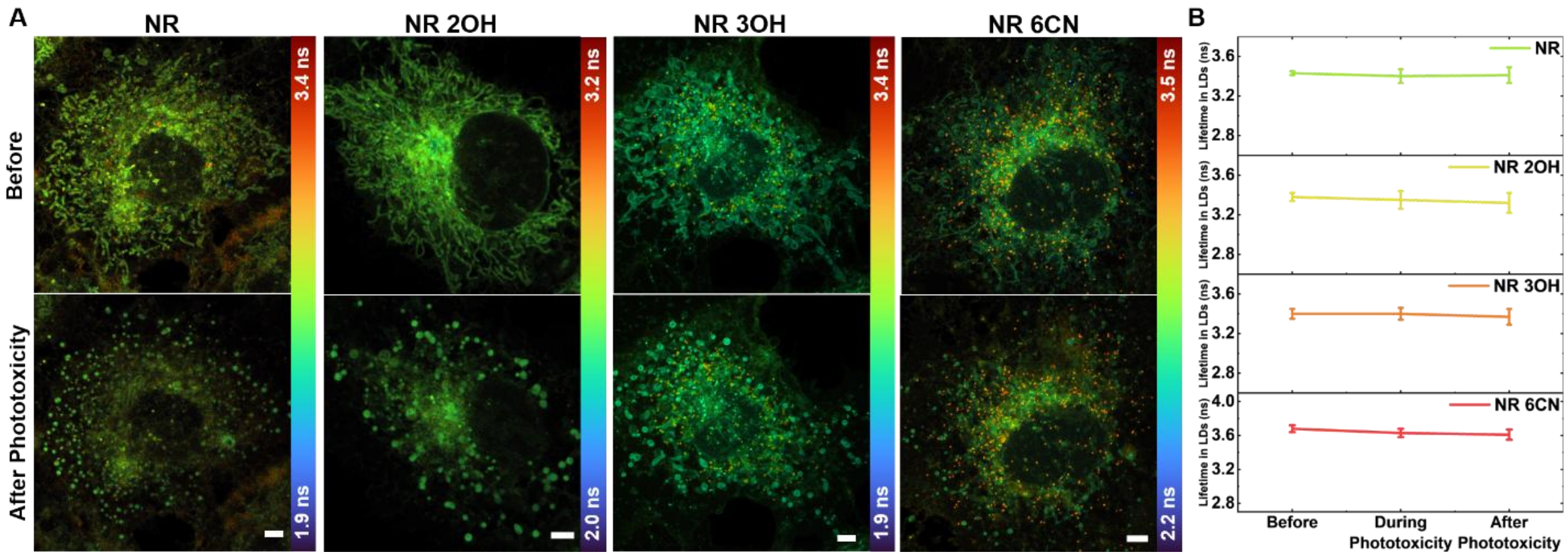

Fig. S39. Phototoxicity during continuous high-power STED acquisition. (A) Representative STED-FLIM images showing phototoxic effects in COS-7 cells stained with NR analogs after continuous high-power STED acquisition (average depletion power, ~5.25 mW; ~10 accumulated frames; frame interval, ~15 s; ~15 s per frame). Scale bars, 5 µm. (B) LD lifetime changes before, during and after phototoxic treatment. Data are mean ± s.d. (n ≥ 3 independent experiments per analog).

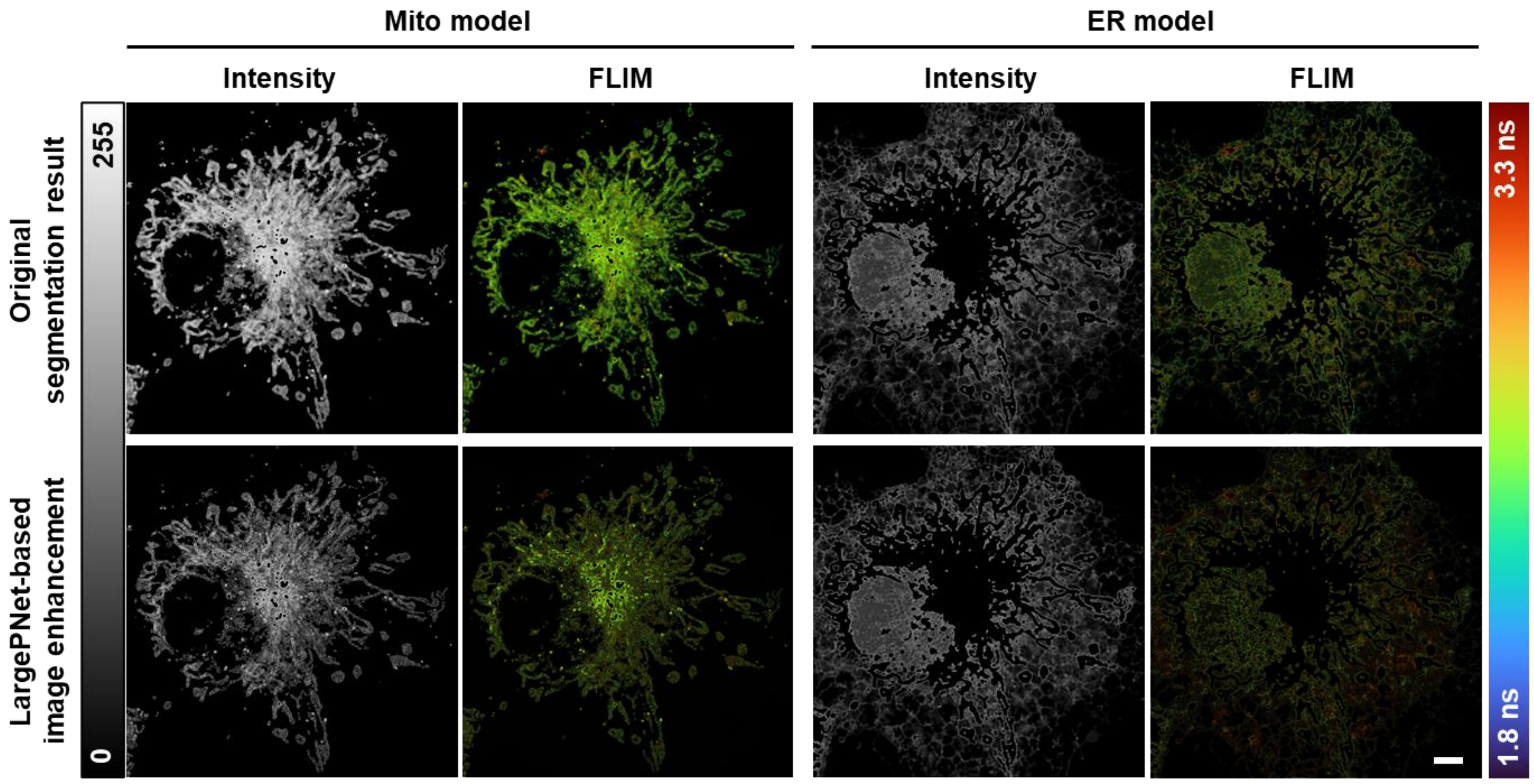

Fig. S40. LargePNet-based restoration improves post-segmentation image quality. Top row, original segmentation results obtained using the VGG16-UNet model (mitochondria and ER models). Bottom row, segmentation results after LargePNet-based image enhancement, showing improved delineation of mitochondrial and ER structures. Scale bar, 5 µm.

Table S1. Fluorescence excitation and emission of NR analogs in different solvents.

Solvent	NR		NR 2OH		NR 3OH		NR 6CN	
	λ_{max}^{ex} (nm)	λ_{max}^{em} (nm)	λ_{max}^{ex} (nm)	λ_{max}^{em} (nm)	λ_{max}^{ex} (nm)	λ_{max}^{em} (nm)	λ_{max}^{ex} (nm)	λ_{max}^{em} (nm)
Et_2O	514	576	500	574	513	575	565	615
PhMe	524	579	507	575	522	579	570	614
1,4-Dioxane	521	586	514	586	522	591	565	617
EA	524	594	515	588	521	598	573	634
THF	529	599	520	593	526	597	572	633
ACN	534	621	530	615	532	622	585	643
DMSO	552	633	546	629	547	636	596	659

Table S2. The fluorescence lifetime of NR analogs in toluene (PhMe), tetrahydrofuran (THF), acetonitrile (ACN), and DMSO.

Solvent	NR: τ (ns)	NR 2OH: τ (ns)	NR 3OH: τ (ns)	NR 6CN: τ (ns)
PhMe	3.96	3.83	3.80	4.65
ACN	4.66	4.66	4.67	4.13
THF	4.32	4.22	4.24	4.82
DMSO	4.20	4.27	4.40	3.35

Table S3. The pixel dwell time and total frame time correspond to the setting on LAS X software of Leica Microsystems.

Settings		Pixel dwell time (μs)	Total frame time (s)
Line Repetitions	Speed		
2	200	3.85	10.28
2	400	1.41	5.14
3	200	3.85	15.42
3	400	1.41	7.71

Table S4. Measured average laser power at the sample plane corresponding to the nominal settings in Leica LAS X software.

Settings (%)	Average Power (mW)		
	λ_{ex} : 550 nm	λ_{ex} : 580 nm	λ_{STED} : 775 nm
1	0.002	0.001	-
2	0.002	0.002	-
5	0.002	0.002	-
50	-	-	4.19
55	-	-	4.68

Movie S1. Time-lapse STED imaging of live-cell stained with NR 3OH revealed organelle interactions.

Movie S2. Time-lapse STED imaging of live-cell stained with NR 3OH revealed mitochondrial fusion.

Movie S3. Time-lapse STED-FLIM imaging of live-cell stained with NR 3OH under normal condition.

Movie S4. Time-lapse STED-FLIM imaging of live-cell stained with NR 3OH after treated with erastin (5 µM, 3 h).

Movie S5. Time-lapse STED-FLIM imaging of live-cell stained with NR 3OH under stimulation with rotenone (5 µM).
